# Management of diseases in a ruminant livestock production system: a participatory appraisal of the performance of veterinary services delivery, and utilization in Ghana

**DOI:** 10.1186/s12917-023-03793-z

**Published:** 2023-11-15

**Authors:** Francis Sena Nuvey, Gloria Ivy Mensah, Jakob Zinsstag, Jan Hattendorf, Günther Fink, Bassirou Bonfoh, Kennedy Kwasi Addo

**Affiliations:** 1 Swiss Tropical and Public Health Institute, Kreuzstrasse 2, Allschwil, 4123 Switzerland; 2 Faculty of Medicine, University of Basel, Klingelbergstrasse 61, Basel, 4056 Switzerland; 3 Department of Bacteriology, Noguchi Memorial Institute for Medical Research, University of Ghana, P.O. Box LG 581, Accra, Ghana; 4 Faculty of Science, University of Basel, Klingelbergstrasse 50, Basel, 4056 Switzerland; 5 Centre Suisse de Recherches Scientifiques en Côte d’Ivoire, Abidjan, BP 1303 Côte d’Ivoire

**Keywords:** Diseases management, Antimicrobials, Performance of veterinary services, Ruminant livestock, Livestock diseases, One Health

## Abstract

**Introduction:**

Sustainable livestock production remains crucial for attainment of food security globally and for safeguarding the livelihoods of many households in low- and –middle income countries. However, the high prevalence of infectious livestock diseases, coupled with inadequate provision and adoption of effective control measures, leads to reduced livestock productivity, increased animal mortalities, and emergence of antimicrobial resistant pathogens. This study sought to assess the management strategies employed by farmers for priority diseases affecting their animals and the utilization and performance of veterinary services.

**Methods:**

We conducted the study in three districts, namely, Mion, Pru East, and Kwahu Afram Plains South Districts, which represent the main livestock production belts in Ghana. We used questionnaires in surveys, to collect pertinent data from 350 ruminant livestock farmers and 13 professional veterinary officers (VOs) in the study districts. Additionally, we conducted seven focus group discussions (FGDs) with 65 livestock farmers in the study districts. The survey data was analyzed, and we describe the distribution of the priority livestock diseases, the disease management strategies employed, and the performance of veterinary services in Ghana. We also analyzed the raw FGD transcript texts deductively based on the study objectives. To validate findings across the different datasets, we used triangulation.

**Results:**

Almost all the farmers (98%) reared small ruminants, with about 25% also rearing cattle. The main priority livestock diseases identified includes pestes-des-petits-ruminants and mange infection in sheep and goats, as well as contagious bovine pleuropneumonia and foot-and-mouth-disease in cattle. We found that majority (82%) of the farmers relied on treatment, while only 20% opted for vaccination services. Additionally, the veterinary system in Ghana did not adequately regulate the antimicrobial medications employed by farmers to manage diseases. Thus, in most of the cases, the medicines applied by farmers were not useful for the target diseases. Although our findings show the farmers perceived VOs to perform highly compared to informal providers on most of the attributes evaluated including medicine availability and quality, treatment effectiveness, advisory services, service affordability, and competence, only 33% utilized VOs services. The majority of the farmers (51%) used the services of informal providers, who were better in proximity and popularity with farmers.

**Conclusions:**

The livestock sector in Ghana faces a substantial challenge due primarily to vaccine-preventable diseases. Even though VOs demonstrated superior performance on key veterinary service performance indicators, their services are underutilized by livestock farmers. Additionally, the absence of regulatory oversight by the veterinary system over antimicrobials utilized in animal production contributes to their misapplication by livestock farmers, posing a considerable risk to both public health and food security. It is thus imperative to introduce new initiatives that enhance the uptake of animal vaccines and better antimicrobial stewardship to ensure sustainable livestock production.

**Supplementary Information:**

The online version contains supplementary material available at 10.1186/s12917-023-03793-z.

## Introduction

In spite of the strides made in the last decade towards attaining the Sustainable Development Goals (SDGs) the risks for severe food insecurity and extreme poverty have increased in recent years. The main drivers of the recent bottlenecks to food security have been climate change, COVID-19 pandemic, conflicts, global economic crisis and increasing supply chain constraints [1]. In light of these difficulties, urgent strategies are required to improve country-level productivity in the agricultural sector to address the food system challenges [1].

Although more than 50% of people in sub-Saharan Africa (SSA) are employed in the agricultural sector [2], the region’s productivity in agriculture is comparatively low globally [3]. The agricultural sector in many countries in SSA is primarily dominated by crop production, which accounts for more than two-thirds of the production levels in the sector [4]. Livestock production, despite comprising less than a third of total agricultural output, plays a crucial role in the lives of people in SSA. Livestock serve as essential assets, contributing to various aspects of people's lives. They serve as a source of livelihood, protein, and wealth storage against uncertainties. In addition, they also serve as companion animals for many farmers [5–8]. The livestock production system is largely extensive and dominated by small-scale farmers. The animals’ productivity thus is affected greatly by weather changes, availability of grazing resources, livestock diseases, security and conflict, and access to veterinary services [9, 10].

Our previous study in Ghana showed that farmers experience significant livestock mortalities primarily due to infectious animal diseases, theft, pasture shortages and conflicts. These factors collectively resulted in an average annual herd loss of 15%, and affecting approximately 80% of livestock farmers [11]. The negative impacts of these adverse events are further exacerbated by inadequate provision of veterinary services, which could enable farmers to better cope with the challenges, due to limited government investment in the veterinary sector [12].

The veterinary system plays a crucial role in providing both preventive and curative services to livestock farmers. However, a recent review of the performance appraisal reports on veterinary services across SSA countries revealed that 80% of countries in the region face significant limitations, or in some cases, a complete lack of administrative control over the registration, import and production, distribution and use of veterinary medicines and biologicals [13]. Consequently, the usage rates of antimicrobials in the region are considerably high, varying from approximately 80% to 100% of all farms. The commonly used antibiotics are tetracyclines, aminoglycosides, and penicillin groups [14, 15]. This high usage of antimicrobial drugs in the absence of formal controls can lead to the persistence of drug residues in livestock products, and promote the development of antimicrobial resistant pathogens.

There is currently no reliable overview on how Ghanaian farmers prevent and manage livestock diseases affecting their herds and how they interact with their veterinary service providers. The previous research on disease management practices in Ghana were narrow in scope; dealing specifically with control of only particular diseases [16, 17], or management practices employed by one veterinary provider [18] or farmers in one agro-ecological zone [19, 20]. To be able to address the food safety concerns with sustainable policies in Ghana, it is essential to identify the current disease management strategies employed by both farmers and their veterinary service providers, particularly for priority disease conditions. Our study sought to address the identified gaps. We assessed the most important adversities affecting livestock production including main diseases from both the farmers and veterinarians’ perspective, and assessed the utilization rate of professional veterinary services and the factors predicting it, as well as evaluated farmers’ perception of the performance of their veterinary service providers.

## Materials and methods

### Description of study area

**Fig. 1:**
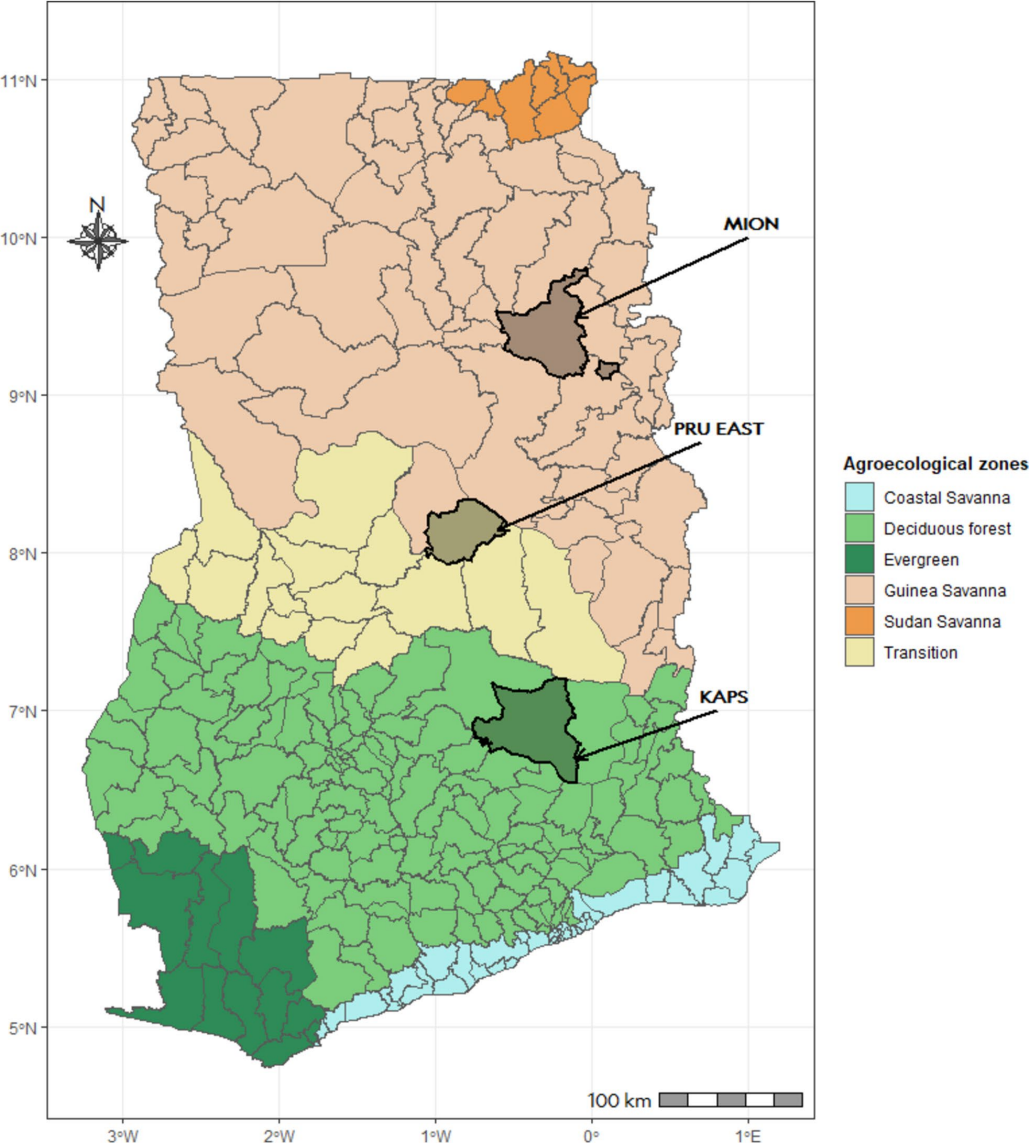
Administrative map of Ghana showing the agro-ecological zones and study districts (The figure shows the district-level administrative and ago-ecological map of Ghana. It presents the distinct locations of the study districts (shaded areas to which arrows point) within the main agro-ecological zones. MION, PRU EAST, and KAPS denote the Mion, Pru East and Kwahu Afram Plains South Districts respectively. The map was created by the authors)

This study was conducted in the Mion, Pru East and Kwahu Afram Plains South (KAPS) districts, which represent the northern, middle, and southern farming belts in Ghana. The districts lie in the Guinea Savannah, Transition and Deciduous forest Vegetation zones, which are the main livestock production zones in Ghana (Fig. 1). The selection of districts was done purposively in collaboration with the regional directors of veterinary services, utilizing a sampling frame of farming districts within the corresponding vegetation zones. The districts were selected based on their strategic location and suitability for conducting field studies. These districts are primarily rural and agrarian. Cattle, sheep, and goats are the predominant ruminant livestock species reared in these districts [21–23]. We obtained district maps from which we extracted a sampling frame of villages to obtain a random sample for data collection.

### Study design

This was a cross-sectional study employing a convergent parallel mixed-method approach. This design enables the simultaneous integration of quantitative and qualitative elements of a research project within the same phase of the research process. Although the analysis is performed separately for each method, the results carry equal significance and are jointly interpreted [24]. We conducted two cross-sectional surveys involving 350 livestock farmers and 13 professional veterinary officers (VOs), and seven focus group discussions (FGDs) involving 65 livestock farmers purposively selected within the districts. The FGD participants were selected based on their knowledge and experience with livestock farming in the districts, as well as their willingness and ability to travel to a designated venue on scheduled dates.

### Study population

In the livestock farmers’ survey, we firstly created a sampling frame of villages within the study districts. Based on published data from the last census (2010 population and housing census) conducted before the study, we randomly drew 15 villages in the KAPS District, and 10 villages each in the Pru East and Mion Districts, proportional to the number of livestock farming households per study district [21–23]. From the selected villages, at least two persons were approached per village to participate in FGDs organized after the surveys in each district. Seven FGDs; three in KAPS District and two each in the Pru East and Mion Districts were conducted involving 65 participants. All the VOs in the study area also participated in a survey to identify key challenges facing livestock production, veterinary service delivery and treatment strategies used for key diseases in the study area. Based on the census data available prior to the study, there was about 29,890, 11,250, 8740 veterinary livestock units (VLUs) in the KAPS, Mion and Pru East districts respectively. VLUs depict the workload per veterinary officer calculated by dividing the standardized total number of animal heads in tropical livestock units (TLUs) by the number of VOs [12]. In Ghana, the VOs providing veterinary services in rural areas, where livestock are usually reared, are mainly veterinary paraprofessionals with a diploma degree in animal health as minimum qualification. The VOs work under the supervision of district or regional veterinarians (with a doctor of veterinary medicine qualification) [12]. All the VOs assigned in the study districts were veterinary paraprofessionals.

### Sample size and sampling technique

We determined the sample size using Epi Info Companion version 5.5.9 [25] with the following assumptions:

Expected utilization rate of veterinary services was 60% based on a previous study in the Northern region of Ghana, which found in a survey that 57% of livestock farmers used government veterinary services [26]. The acceptable margin of error was 5%-points, at a 95% confidence level. This yielded a minimum sample size of 370 livestock farmers. We recruited 350 livestock farmers from 38 villages using segmentation, with a response rate of 95%. The 5% non-response was mainly due to low number of ruminant livestock keeping households in some of the randomly assigned study villages during enumeration. Within the selected segments of the study villages, all households engaged in ruminant livestock farming were eligible for selection to participate in the survey. Households that provided consent were recruited to partake in the survey. For the VOs survey, all personnel were eligible to participate once informed consent was given. All 13 VOs assigned within the districts were recruited. For the FGDs, we used a purposive sampling approach to recruit farmers during the survey in each district. Overall, 65 farmers consented to participate in FGDs in the three districts.

### Data collection and data management

Between November 2021 and January 2022, the enumeration team visited the households keeping ruminant livestock in their homes to administer the questionnaires. The respondents were interviewed in one of four Ghanaian languages (English, Akan, Dagbani or Ewe) face-to-face using tablets equipped with Open Data Kit (ODK) application. The survey instruments were designed to collect data on priority diseases affecting herds, management strategies employed, farmers’ perception of the performance of veterinary service providers and other socio-demographic characteristics. The livestock farmers’ appraisal of the performance of professional and informal veterinary service providers was assessed using a questionnaire adapted from Admassu et al. [27]. We also assessed the utilization of professional veterinary services by farmers, and factors predicting the utilization (Additional file 1).

The VOs survey were conducted during the same period at the workplaces of the veterinary personnel using ODK. In their survey, VOs evaluated their own performance on several key functions of veterinary services including the availability of border posts for monitoring animal movements, slaughter places for ensuring meat safety, motor vehicles for delivery of veterinary services, designated laboratories for confirming suspected pathogens, and protocols for regulating the sale of medicines within their respective operational areas. We assessed the availability of communication pathways between the VOs and public health personnel regarding the control of zoonotic diseases in the operational areas. We collected data on the priority diseases or conditions and specific drugs used to treat or manage the affected animals (Additional file 2).

The FGDs were conducted concurrently with the other field studies at designated venues in the study districts using a paper-based interview guide, and the sessions recorded using an audiotape. The farmers discussed in the FGDs the main constraints of livestock production, disease management strategies applied and factors influencing their choices. They also reported the distribution in a farming year, the priority or most common diseases affecting both small and large ruminants in their herds using the proportional piling approach. Specifically, the farmers distribute for each disease that affects their herd, the proportion on average of 10 round counters presumed as their total herd, that get infected during a farming year for each disease.

The survey data was downloaded as Microsoft Excel files from ODK and imported into R version 4.1 [28] for analyses. The interview audio recordings from the FGDs were transcribed verbatim, and the transcripts imported into NVivo software version 12 [29] for analysis.

### Data analyses

We performed descriptive analyses of both livestock farmers and VOs surveys, comparing the distribution of responses by study district. The farmers’ herd sizes were converted to tropical livestock units (TLU) to standardize livestock holdings as follows: 1 TLU = 0.75 cattle, or 0.2 pigs, or 0.1 small ruminants, or 0.01 poultry, or 0.02 doves [30]. We also compared livestock farmers’ perception of the severity of different adversities on herds with the perspectives of the VOs. We assessed the priority diseases affecting ruminant livestock, with farmers and VOs reporting the most recent disease(s) or condition(s) to cause deaths of the animals. We report the frequency of use of the medicines, and the usefulness of the medicine and disease or condition combinations based on the evidence in literature and authors’ experience. We also compared how farmers and VOs treat the common or priority diseases affecting livestock in the districts. The performance of the professional and informal veterinary service providers on each of the attributes or indicators were derived by transforming the Likert scale scores into Relative Importance Indices (RIIs) as follows:

$$\mathrm{Relative\ Importance\ Index}= \frac{\sum W}{A\times N}$$

Where W is the weight given to each attribute or indicator by the respondents (ranging from 1 to 5), A is the highest weight (i.e., 5 in this case), and N is the total number of respondents.

Using a pre-specified model, we evaluated the relationship between professional veterinary service utilization (any use in the past 12 months), and farmers’ sex, educational attainment, herd size, wealth status, resilience level, livestock rearing experience, distance to VOs, perception of disease severity at herd-level, and level of social support received, adjusting for village-level clustering, at the 95% confidence level in a binary logistic regression model. We determined the relative wealth of households using an index of a household’s ownership of selected assets, such as televisions, refrigerators and bicycles, presented as wealth quintiles [31]. Resilience was assessed using the Resilience scale (RS-14). The RS-14 consists of 14 items rated on a 7-point Likert-scale. The total scores are computed and higher scores indicate higher resilience [32]. The availability of social support to farmers was assessed using a 5-point Likert scale, which measured the level of support farmers received from various facets of society including family, friends, law enforcement, credit institutions, community leaders and religious leaders, to aid them in livestock farming. Herd size and social support level were categorized into tertiles (three quantiles) to compare veterinary service utilization within homogenous levels. The choice of covariates for the model was pre-specified, and was informed mainly by literature and previous research on the determinants of veterinary services utilization [33–35]. Potential violations of the model assumptions were assessed by calculating Pregibon leverages, by visual inspection of residual versus fitted and QQ-plots, and by examination of variance inflation factors.

The analysis of FGD transcripts was conducted from a social constructivism perspective recognizing that agricultural (livestock) production is shaped by the social and cultural dynamics of those involved. We sought to find convergence on the social concerns regarding the challenges faced by farmers in rearing ruminant livestock, and strategies they employ to tackle or manage these challenges. We conducted a deductive thematic analysis of the transcripts, by generating codes and categories from the raw transcript texts, based on the study goals. The results are presented in a narrative form supported by verbatim quotes. Where necessary, clarification phrases are placed in square brackets to enhance the understanding of the quotes. We present also the frequency distribution of the reported proportions of large and small ruminant herds affected by the priority diseases. We used triangulation to validate the findings across the different datasets.

## Results

### Socio-demographic characteristics of study participants

Table 1 presents the socio-demographic characteristics of all study respondents stratified by study area. On average, the farmers completing the survey (*N* = 350) were 45 years old (SD = 14 years), with no significant differences in age between the study districts. Furthermore, the farmers reported an average of 9 years livestock rearing experience (IQR = 6 – 15 years), with farmers keeping an average of 2.9 TLUs of livestock (IQR = 1.4 – 7.8 TLUs); including cattle, goats, sheep, pigs, chicken, guinea fowls, ducks, and doves in their herds. Majority of the farmers (95%) own the animals reared. The livestock farming experience and herd sizes were not significantly different between the study districts. The farmers’ resilience similarly did not differ significantly between the study districts, with farmers having average resilience score of 80.5 out of 98 (IQR = 74 – 85). More than two-thirds (71%) of the farmers were male, with the proportion significantly different between districts. About half of the farmers had received no formal education (51%), with the level of educational attainment being significantly different between the study districts. The average number of individuals in farmer households was 8 (IQR = 6 – 11 individuals), and the average distance between the households and professional veterinary officers (VOs) was 8 km (IQR = 1.9 – 12.4 km). The household sizes and distance to VOs were significantly different between the districts. Households’ wealth index also differed significantly between study districts with Mion (59%) and Pru East (69%) Districts having the highest proportion of the poorest and least poor households respectively. Significant differences were observed in the availability of social support between the study districts, with more than half of farmers reportedly receiving low levels of social support. The primary sources of social support reported, in order of availability, were from family, friends, religious bodies, community leaders, credit associations, and law enforcement bodies.

**Table 1:** Socio-demographic characteristics of the study participants by study district

Characteristic	KAPS	MION	PRU EAST	Percent (%)	Statistical significance
**Farmer survey (*N* = 350)**	***n* = 149**	***n* = 98**	***n* = 103**		***p*-value**
**Age (years)**	46.0 (36.0, 56.0)	41.0 (34.0, 51.0)	46.0 (34.0, 57.0)		0.247
**Household size (persons)**	7 (5, 10)	10 (7, 15)	8 (6, 13)		0.021
**Livestock farming experience (years)**	9.0 (5.0, 16.0)	10.0 (6.0, 17.0)	9.0 (5.0, 15.0)		0.415
**Distance to veterinary service (km)**	12.0 (8.0, 14.4)	6.9 (1.6, 12.7)	1.9 (0.6, 5.6)		< 0.001
**Resilience level**	78.0 (73.0, 84.0)	82.5 (78.0, 87.0)	81.0 (75.0, 86.0)		0.431
**Sex**					0.001
Female	57	16	29	29.1	
Male	92	82	74	70.9	
**Educational attainment**					< 0.001
No formal education	41	85	52	50.9	
Up to 12 years education	72	6	29	30.5	
Higher education	36	7	22	18.6	
**Wealth status**					< 0.001
Poorest	21	41	8	20.0	
Below average	41	25	8	21.1	
Average	36	14	16	18.9	
Above average	37	10	23	20.0	
Least poor	14	8	48	20.0	
**Social support availability**					0.012
Low	77	44	59	51.4	
Medium	43	30	13	24.6	
High	29	24	31	24.0	
**Herd size (TLU)**					0.011
Small (1st tertile: 0.3 – 1.8 TLUs)	55	41	21	33.4	
Medium (2nd tertile: 1.9 – 5.48 TLUs)	51	28	38	33.4	
Large (3rd tertile: 5.5 – 182.3 TLUs)	43	29	44	33.2	
**Farmer FGD (*N* = 65)**	***n* = 30**	***n* = 15**	***n* = 20**		
**Age (years)**	46.0 (37.0, 54.0)	41.0 (36.0, 52.0)	49.0 (35.5, 56.0)		0.763
**Herd size (TLU)**					0.297
Small (1st tertile: 0.9 – 2.1 TLUs)	9	7	6	33.8	
Medium (2nd tertile: 2.2 – 11.6 TLUs)	10	5	7	33.8	
Large (3rd tertile: 11.7 – 157.2 TLUs)	11	3	7	32.4	
**Sex**					0.300¥
Female	7	1	2	15.4	
Male	23	14	18	84.6	
**Educational attainment**					0.025¥
No formal education	16	3	7	40.0	
Up to 12 years education	5	10	7	33.8	
Higher education	9	2	6	26.2	
**Veterinary officer survey (*N* = 13)**	***n* = 3**	***n* = 5**	***n* = 5**		
**Age (years)**	32.0 (32.0, 43.0)	33.0 (32.0, 41.0)	30.0 (29.0, 38.0)		0.454
**Veterinary training (years)**	3.0 (3.0, 5.0)	3.0 (3.0, 4.0)	3.0 (3.0, 4.0)		0.467
**Work experience (years)**	2.0 (2.0, 15.0)	2.0 (2.0, 9.0)	2.0 (2.0, 7.0)		0.440
**Sex**					1.000¥
Female	0	1	1	15.4	
Male	3	4	4	84.6	

Numbers (n) of participants, including farmers and veterinary personnel by data collection approach, falling into each study district; KAPS denotes participants from the Kwahu Afram Plains South District, MION denotes participants from the Mion District and PRU EAST denotes participants from the Pru East District in the Southern, Northern and Middle farming Belts of Ghana respectively. Percent (%) denotes the proportion of study participants within each characteristic explored. TLUs denotes farmers’ herd sizes standardized in tropical livestock units (1 TLU = 1 cattle, or 3 pigs, or 5 small ruminants, or 25 poultry, or 50 doves). For continuous variables, the median value with corresponding lower and upper quartile values reported in parentheses are presented. *P*-values from Kruskal–Wallis equality-of-populations rank test for continuous variables, and *p*-values from Chi-square tests for categorical variables are presented. ¥ denotes Fisher’s exact test probabilities for expected observations less than 5 persons in at least one of the cells

For farmers participating in the focus group discussions (FGDs) (*N* = 65), average age was 45.5 years (SD = 13.0 years). On average, the farmers keep 3.6 TLUs in their herds (IQR = 1.7 – 25.5 TLUs). The majority of the participants were male (85%). There were no significance differences observed in the age, herd size and sex distributions of farmers participating in the FGDs between the study districts. On average, majority of the farmers (60%) participating in the FGDs had at least some basic formal education, with educational attainment levels being significantly different between the study districts.

The VOs in the study districts were 36.1 years old (SD = 8.6 years) on average, with a majority (85%) being males. They have undergone an average of 3 years (IQR = 3 – 4 years) of veterinary training and worked on average for 2 years (IQR = 2 – 9 years) in the veterinary services. The age, sex, years of training and work experience did not differ significantly between the personnel by study district.

### Severity of adverse events affecting livestock farming

Table 2 presents the top five ranked adverse events based on severity of impact on herds by farmers and their VOs stratified by study district. Overall, the adverse event ranked to have the most severe effect on livestock production by the majority of the participants (all livestock farmers and most of the VOs) across all the study districts was animal diseases. The severity ranking of the other adverse events were mainly district dependent. Pasture shortages was also ranked highly in all the districts although in the more arid Mion District than the other districts. Bush fires were ranked third by farmers in the Mion District, but is less of a challenge in the other two districts. While theft of animals was ranked second by farmers in the Pru East District, conflicts with other land users was ranked second by the farmers in the KAPS District.

**Table 2:** Most important adversities affecting livestock production based on reported severity of impact on herd by study district

**Farmer Survey (*N*** **= 350)**	**KAPS (*n*** **= 149)**	**MION (*n*** **= 98)**	**PRU EAST (*n*** **= 103)**
**Adverse Events**	**Ranking**		
Animal diseases	1st	1st	1st
Pasture shortages	3rd	2nd	3rd
Conflict with other land users	2nd	-	= 5th
Theft of animals	4th	-	2nd
Bush fires	-	3rd	= 5th
Water shortages	-	5th	
Swallowing of foreign bodies	5th	4th	4th
**Veterinary Officer Survey (*N* = 13)**	**KAPS (*n* = 3)**	**MION (*n* = 5)**	**PRU EAST (*n* = 5)**
**Adverse Events**	**Ranking**		
Animal diseases	1st	= 1st	= 4th
Pasture shortages	= 4th	= 1st	= 4th
Conflict with other land users	= 2nd	= 2nd	= 2nd
Theft of animals	= 4th	= 2nd	1st
Bush fires	= 4th	-	-
Water shortages	-	-	-
Swallowing of foreign bodies	= 2nd	= 3rd	= 2nd

Numbers (n) of participants, including farmers and veterinary personnel included in surveys, falling into each study district; KAPS denotes participants from the Kwahu Afram Plains South District, MION denotes participants from the Mion District and PRU EAST denotes participants from the Pru East District in the Southern, Northern and Middle farming Belts of Ghana respectively. The adverse events included in the table are events for which ranked first to fifth per study district. Rankings range from 1st = Most severe impact on herds to 5th = Least severe impact on herds. Rankings are derived as the sum of the products of the number of participants (n) and the reported severity level (no effect = 1, moderate effect = 2, and severe effect = 3) for each adverse event, standardized by sample size per study district. Rankings with “ = ” before the rank denote tie rank scores for adverse events within each study district. We omitted rankings below the 5^th^ rank within each study district. The other adverse events include poor market for livestock, flooding and ectoparasite infestations

The VOs perception of adverse events’ effect on livestock production was mainly district dependent. While the personnel in the KAPS and Mion Districts ranked animal diseases to have the most severe effect on livestock production, VOs in the Pru East ranked it fourth. The highly ranked challenge in the Pru East District by VOs was theft of animals. A majority of the personnel across the districts perceived livestock farmers’ conflicts with other land users, and ingestion of foreign objects like polythene rubbers by animals to have moderate to severe effects on livestock production. The perceptions of the severity of the adverse events generally was different from the farmers’ and the veterinary personnel’s perspective in the study district, except for the impact of animal diseases on herds.

In the FGDs, the farmers reported animal diseases as the main challenge facing the livestock farmers. The reported challenges were mainly animal-health related, with major concerns about the effects of the diseases on herd productivity and livelihoods, lack of animal health infrastructure, low access to, and inadequacy and high cost of animal health service provision.

*“For us we have a big problem with diseases in our animals. When it comes to cattle, there is a disease called ‘suffer’ [Foot-and-Mouth Disease (FMD)], it worries us a lot. There are also other diseases but the ‘lung disease’ [Contagious Bovine Pleuropneumonia (CBPP)] is very serious. When they are infected, it brings out all other diseases that are hidden in the cattle. …, as for it when it enters the cattle kraal, hmm masa, unless you solve it, you cannot have peace of mind, …, you will weep before it goes. … When they contract the lung disease [CBPP], the pregnant cows do have premature births. Also, the milk production goes down drastically, [long pause], you can’t even get some of the milk. It will not be enough for the calves before you think of the farmer” ****(Male farmer, 46 years old, Mion District)***

*“Formerly, it would have been after 2 to 3 years before you inject your cattle once, but now within 1 month you could treat one cattle about 3 times for diseases” ****(Male farmer, 49 years old, KAPS District)***

Other challenges reported were in relation to pasture and water shortages, housing challenges for the herds, and the cost of resolving conflicts occasioned by animals’ destruction of farms.

*“The diseases are the single major problem for all of us. I think NH [referring to another FGD participant] also said something about feeding. When it gets to the dry season, there is bush burning everywhere. The pasture that cattle and goats will feed on, all of them, off [burnt]. When that happens, the animals begin to lose weight. So when something small [disease] infects them, then they begin to die. .... Hmm, also, the issue of housing, like our sister said; where the animals will sleep [is a problem]” ****(Female farmer, 46 years old, KAPS District)***

*“One major problem for us livestock farmers in this community especially the cattle and goat farmers is that, during the dry season, we [livestock farmers] do not get access to feed for the animals. As a result, it leads to a fight between livestock farmers and the crop farmers especially yam growers. Because the animals sometimes end up destroying the [crop] farms” ****(Male farmer, 53 years old, Pru East District)***

To identify the most common or priority livestock diseases affecting ruminant livestock production, we inquired about the most recent disease cause of death in the past 12 months for farmers reporting a mortality of their ruminant livestock within the study period. About 82% (282/344) of the farmers who rear small ruminants reported animal mortalities compared to 78% (68/87) for the farmers rearing cattle. Specifically for the ruminant livestock species kept (cattle, sheep, and goats), the farmers reported an average of 10% mortality of their herds to diseases (IQR = 23%) in past 12 months. On the most likely disease to have caused the death, 40% of the small ruminant farmers reported Peste-Des-Petits-Ruminants (PPR), while 5% reported mange. Among farmers rearing cattle, 31% reported the recent mortality to be due to Contagious Bovine Pleuropneumonia (CBPP) with about 5% due to Foot-and-Mouth Disease (FMD) infection. In the local languages, farmers refer to PPR, Mange, CBPP and FMD as “ayamtuo yareɛ”, “krusakrusa”, “akoma yareɛ” and “suffer” respectively. The other non-specific factors reported to lead to mortalities were birth-related including abortions and birth complications during parturition, causing about 1% of reported deaths in small ruminants and 4% of reported deaths in cattle.

The VOs similarly provided the disease(s) likely to have caused the most recent reports of ruminant livestock mortality they received from the livestock farmers in their respective operational areas. In small ruminants, PPR was the most likely cause of death in small ruminants, reported by farmers to a majority of the VOs (69%). While in cattle, CBPP was most likely the cause of death reported to the VOs (31%).

**Fig. 2:**
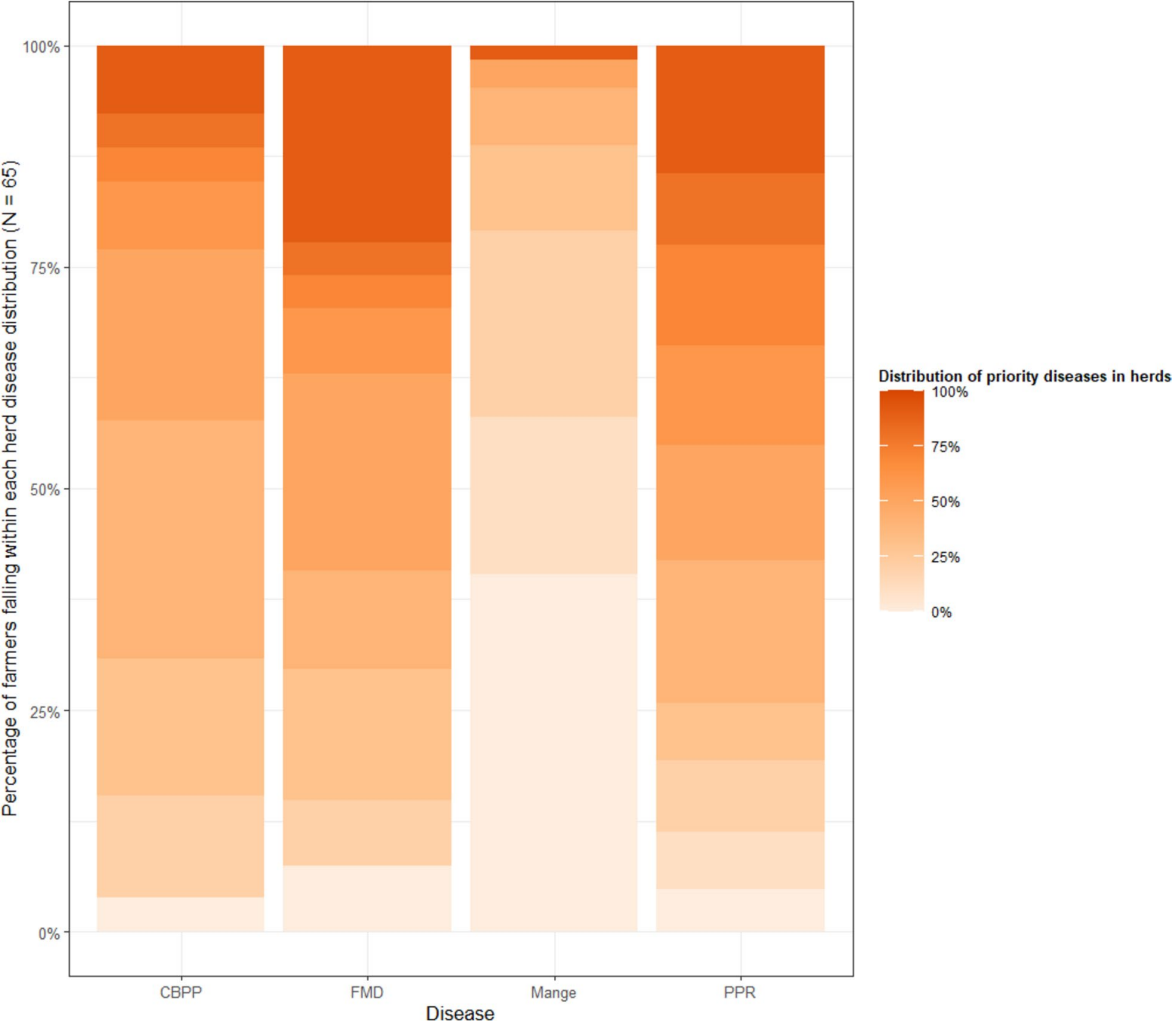
Distribution of common infectious diseases in ruminant livestock (The figure shows the typical distribution of key infectious diseases in a farming year in farmers’ herds based on proportional piling by 65 experienced farmers purposively selected to participate in focus group discussions. The gradient of color shows the reported distribution of the diseases on a percentage scale (from 0 to 100%) with light coloration depicting low prevalence and deep coloration depicting high prevalence. The y-axis shows the proportion of the farmers reporting a specified prevalence level of each disease condition in their herds. Each column bar on the x-axis depicts the two most common diseases for large and small ruminant farmers respectively)

In FGDs, farmers identified the most common diseases affecting their herds to include CBPP and FMD in cattle and, PPR and mange in goats and sheep. Based on this, the farmers reported the average distribution of each disease in their herds in a farming year, using proportional piling. Overall, the reported FMD prevalence was 50% on average (IQR = 50%), while CBPP prevalence was 40% (IQR = 20%) on average in cattle herds in a farming year. For the small ruminants, an average PPR prevalence of 50% (IQR = 40%) and average mange prevalence of 10% (IQR = 20%) among herds in a farming year were reported (Fig. 2).

### Management strategies for common livestock diseases

**Fig. 3:**
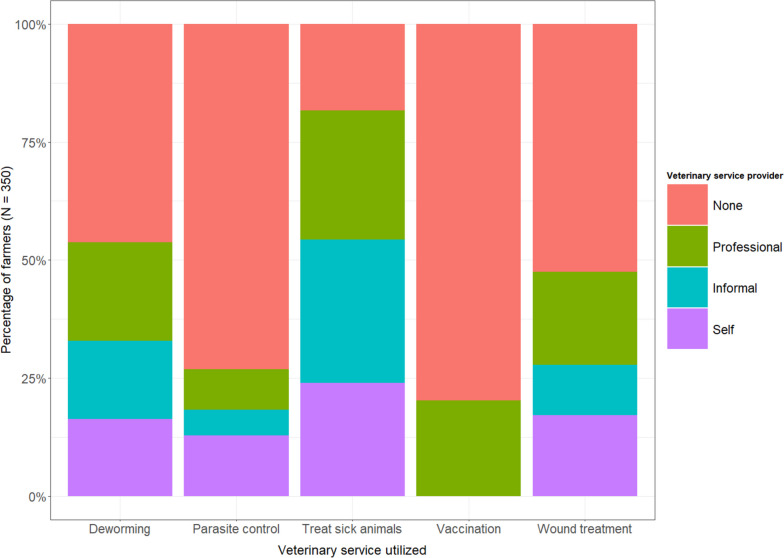
Veterinary services use preferences of ruminant livestock farmers in Ghana (Fig. 3 presents the distribution of most recent usage of key veterinary services requiring the application of medicines by farmers and the service providers used. The y-axis shows the proportion of farmers utilizing the services by provider type. The x-axis presents the services evaluated. The divisions and colors in the stacked bars depict the proportion of each service use accounted for by a service provider)

We found treatment for sick animals (82%), deworming (54%) and treatment of wounds (47%) as the most common disease management strategies the farmers utilized. Only 20% of farmers reported vaccinating their herds in the study year (Fig. 3). We assessed for each disease management strategy utilized by the farmers, the most recent veterinary service provider that rendered the service. Among farmers who used any of these services, the treatment services for sick animals were done almost evenly by informal providers 37% (106/286), professional veterinary officers 35% (96/286) and farmers themselves 29% (84/286). The VOs 39% (73/188) mostly did deworming of the animals, while the farmers and informal providers do about 30% each of the deworming of livestock. Similarly, the treatment of wounds was done mainly by the VOs 42% (69/166); 36% (60/166) of farmers reported treating wounds themselves, with informal providers delivering 22% of wound treatment services for farmers.

**Fig. 4:**
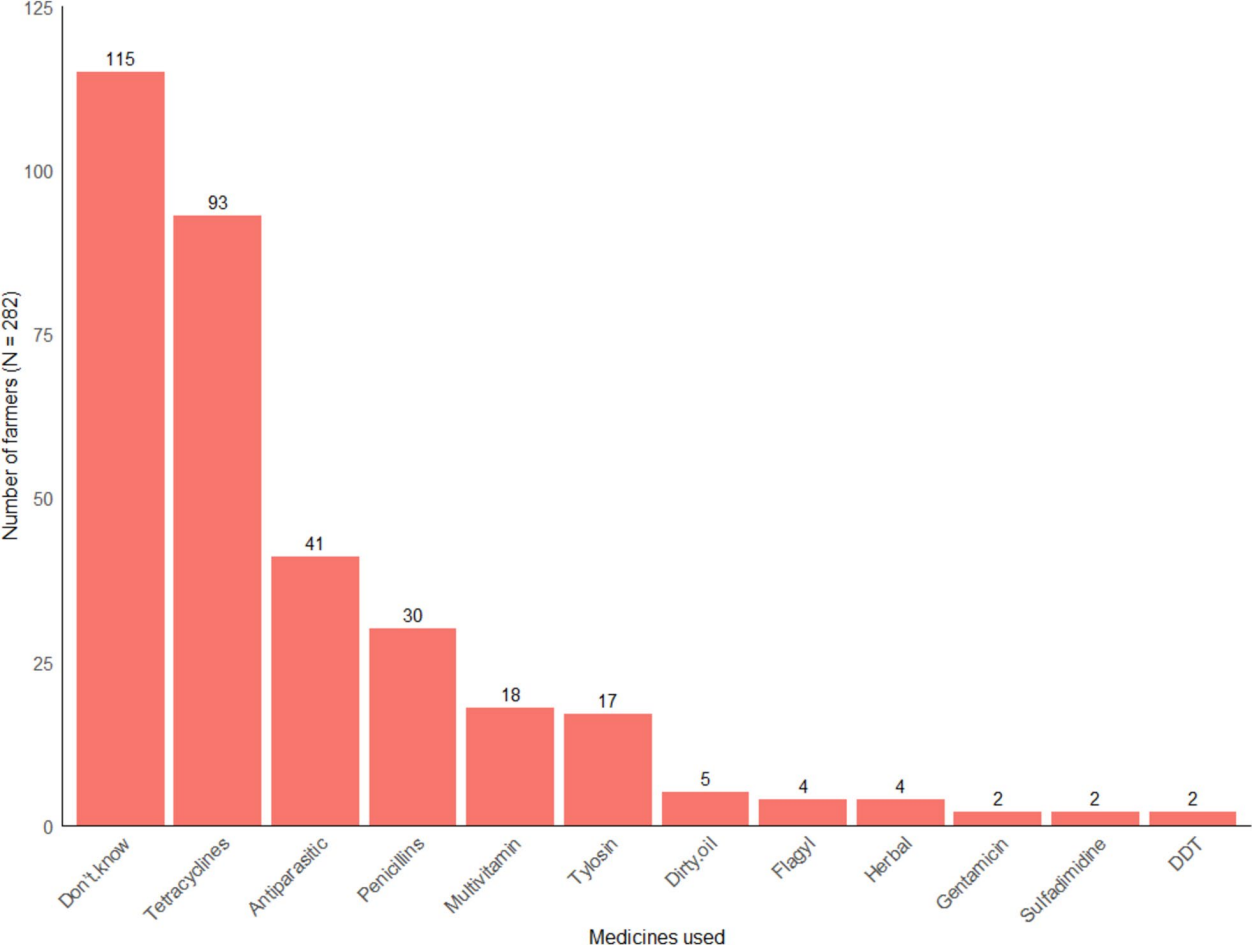
Medicines commonly used by livestock farmers for managing common diseases (The y-axis shows for each medicine used, the number of farmers (*N* = 282) who reported self-treating a disease or condition in their herds during the study year. The x-axis presents the medicines the farmers reported using or have medicine sachets, vials or bottles available during the survey to be captured. Farmers that used medicines but could not recall the medicine names nor provide the medicine sachets, vials or bottles are depicted as “Don’t know” on the column bar. The reported and captured medicines were grouped in medicine classes if possible, and usage frequencies presented in the column bars)

**Fig. 5:**
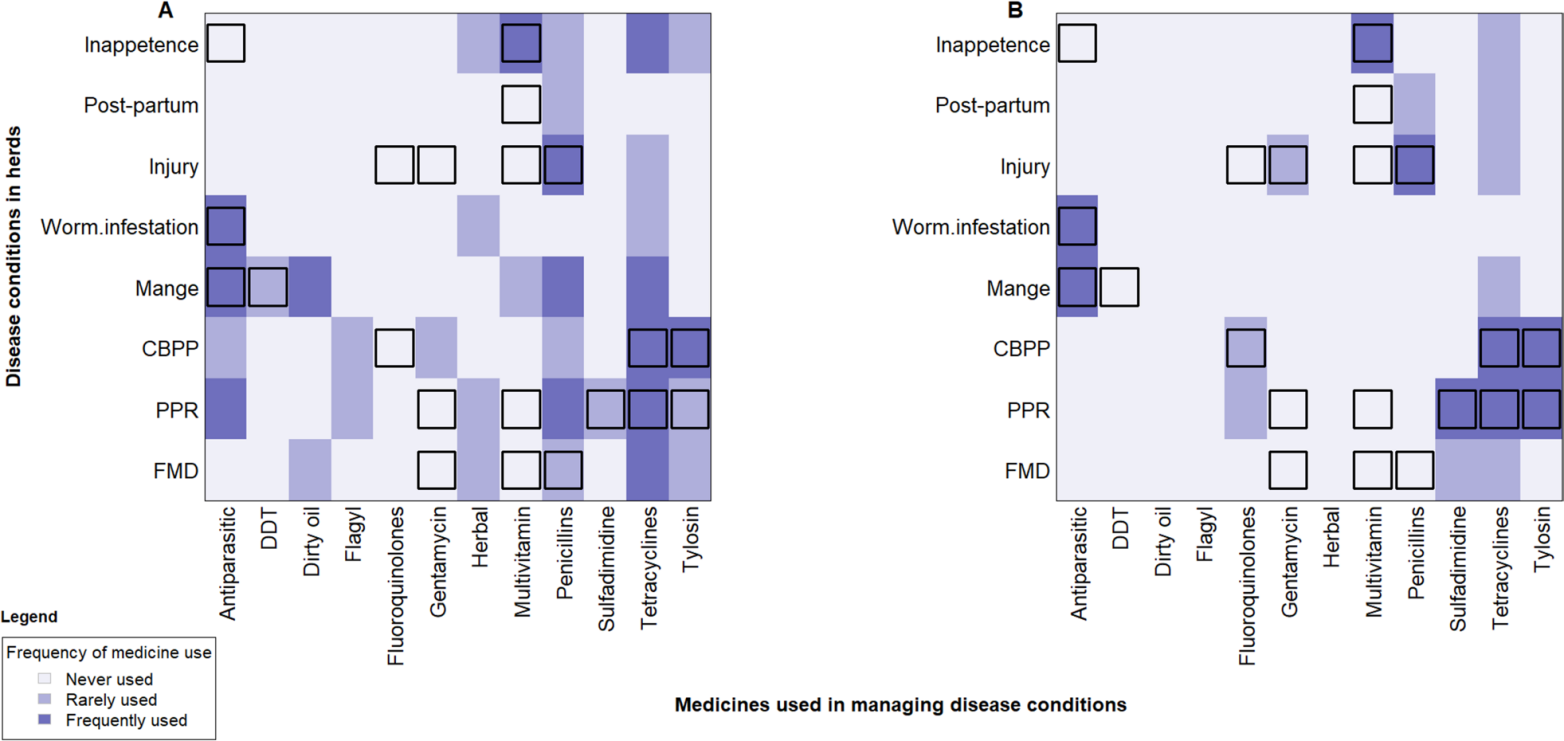
Frequency and utility of use of medicines by livestock farmers and veterinary officers for managing common diseases and conditions in ruminant livestock (Fig. 5 compares the medicines and the reported frequency of use in managing each disease or condition by livestock farmers and professional veterinary officers. Panel A and Panel B depicts farmers’ and veterinary officers’ application of the medicines respectively. Square shapes () in cells depict a useful medicine and disease or condition combination. The color gradient shows the frequency of use of each of the medicines by the study participants, ranging from no use (light color) to frequent use (deep color) respectively. The y-axis presents the disease or conditions treated while the x-axis presents the medicines applied)

As shown in Fig. 4, among livestock farmers who used antimicrobials, the most frequently applied compounds are tetracyclines, penicillins and antiparasitic medicines to manage the diseases or conditions of their animals. The tetracyclines commonly used by the farmers were Oxytetracycline injections, and Tetracycline Hydrochloride capsules. Antiparasitic medicines used commonly were Ivermectin injections, and Albendazole suspensions. With respect to penicillins, farmers commonly used Procaine Penicillin or Procaine Penicillin with Dihydrostreptomycin (PenStrep) injections, and Amoxicillin Trihydrate capsules (See Additional File 3). However, after disaggregation of the application of the medicines, we found that although most farmers use tetracyclines and penicillins for most of the common conditions, the majority of the reported applications of these medicines were not useful (Fig. 5 Panel A). On the other hand, whereas the VOs also use tetracyclines and antiparasitic medicines quite frequently, the reported applications of the medicines were mostly useful for the conditions (Fig. 5 Panel B).

In FGDs, we found that the diseases are treated also with traditional medicines including ethanol, herbal preparations and used automobile engine oils. The orthodox medicines for treatment are accessed from veterinary drug stores, human drug stores, livestock markets or from medicine vendors who roam the communities. Non-orthodox medicines are usually self-made or accessed from livestock markets or community herbalists.

*“… there is not a specific drug, we use different drugs [in FMD treatment], …, everybody tries something. So if you are lucky and a particular treatment works, then you stick with it and it becomes a norm. I remember that a cattle disease [FMD] affected our cattle and someone advised me to buy some medicines to apply, …, The person told me to buy “battery water” [Sulphuric acid] and add a bit of salt to it, …” ****(Male farmer, 37 years old, KAPS District)***

*“…if it [FMD] is serious, we call the veterinary officers to come to assess the affected animal. But sometimes when it is less severe, we buy ‘DDT’ [Acaricide] and mix it with “tupaye” [Tetracycline] and we use the mixture on their affected hooves” ****(Female farmer, 30 years old, Pru East).***

*“…I use procaine penicillin for treating the diarrheal disease [PPR] in both sheep and goats. For the skin rashes [mange], I use ‘dirty oil’ [used automobile engine oil] for it” ****(Male farmer, 41 years old, Mion District)***

For the factors influencing the disease management strategies adopted by the livestock farmers, we found that for the most part, either informal providers administer the treatments or the farmers treat the diseases on their own, due to lack of easy access to professional veterinary officers or reduced severity of the diseases. Few farmers seek professional veterinary officers in the first instance of a disease problem in their herds. The choice of medicines for treating diseases, were greatly influenced by farmers’ past experiences with the use of specific medicines, peer to peer referrals for specific treatment strategies or medicine vendors in communities or livestock markets. The medicines are administered by injections, orally, in drinking water or directly into the mouths with syringes, and topical application on lesions and wounds.

*“Usually, I go to those who sell drugs in ‘gariki’ [livestock market in the district] and explain to them the symptoms the animal is showing. They are the ones that gave me a certain drug [to use for PPR infected small ruminants]. … Some of the drugs are pills, and some too are like milk in a gallon [anthelmintic] and you have to draw it with a syringe to administer it to the infected animals. Some of the drugs are also green in color [anthelmintic tablets], and we give these to them [orally] ****(Male farmer, 50 years old, Pru East District)***

*“For the diarrheal disease [PPR], we use our own mind [knowledge] to manage. Like my brother said, when the goats are having the diarrhea [PPR] and we don’t get the veterinary officer, we go and buy ‘Tupaye’ [Tetracycline],…, and then open a lot of them and put in water; and then open the mouth of the goat and give it to it to drink” ****(Male farmer, 43 years old, KAPS District)***

*“When there is the CBPP outbreak, I call the doctor [veterinary officer] to assess them, and he gave the animals some injections. Sometimes too, if the cattle are not feeding well, the herdsmen will let me know and then we get penicillin injections [procaine penicillin] to be given to the cattle [by the herdsmen]” ****(Female farmer, 46 years old, Mion District)***

The treatment measures employed have varied effectiveness for the conditions they are applied; sometimes they are effective but in most cases, the treatment effects are short-lived.

*“When we use the dirty oil for mange, we see immediate effect of the scaly skin peeling off and new hair regenerate in the affected area in a short while. But it doesn’t cure the disease in the animal’s system because it won’t be long enough and the animals [goat and sheep] would develop the mange again” ****(Male farmer, 41 years old, Mion District)***

*“For the animals that the disease is not advanced, when you administer them [drugs bought from livestock market], it seem to work… but it sometimes recurs. And for those that the disease is already advanced, it doesn’t cure them, and they end up dying ****(Male farmer, 60 years old, Pru East District)***

### Utilization of veterinary services

Overall, only 33% (116/350) of the surveyed farmers utilized professional veterinary services during past 12 months compared to 51% (177/350) utilizing the services of informal veterinary service providers. The proportion of farmers using professional veterinary services was significantly different between the study districts ranging from 54% (56/103) in the Pru East District to 21% (32/149) in the KAPS District (*p* < 0.001). We present the predictors of farmers’ utilization of professional veterinary services in Table 3. In our pre-specified model, we found the odds of farmers utilizing the services to be improved significantly by increasing years of experience with livestock rearing, increase in farmer resilience, increase in herd size, being male, educational attainment, increasing wealth status, increasing severity of perceived effect of diseases on herds and high levels of social support availability in the univariable analyses. While the odds significantly reduced with increase in distance between farmers and professional veterinary officers.

**Table 3:** Factors influencing livestock farmers’ utilization of professional veterinary services in Ghana

	**Unadjusted model**			**Adjusted model**	
**Variables**	**cOR (95% CI)**	***P*-value**		**aOR (95% CI)**	***p*-value**
**Livestock farming experience (years)**	1.04 (1.01, 1.07)	0.014		1.03 (0.98, 1.07)	0.248
**Distance to veterinary service (km)**	0.91 (0.87, 0.95)	< 0.001		0.91 (0.85, 0.98)	0.016
**Resilience level**	1.04 (1.01, 1.07)	0.008		1.03 (1.00, 1.07)	0.078
**Herd size (TLUs)**					
Small (1st tertile: 0.3 – 1.8 TLUs)	ref			ref	
Medium (2nd tertile: 1.9 – 5.48 TLUs)	1.74 (0.95, 3.19)	0.071		1.15 (0.58, 2.28)	0.691
Large (3rd tertile: 5.5 – 182.3 TLUs)	4.09 (2.28, 7.32)	< 0.001		2.11 (1.08, 4.11)	0.028
**Sex**					
Female	ref			ref	
Male	2.22 (1.30, 3.80)	0.004		1.67 (0.90, 3.09)	0.105
**Educational attainment**					
No formal education	ref			ref	
Up to 12 years education	1.70 (1.02, 2.83)	0.041		2.08 (1.07, 4.06)	0.031
Higher education	1.65 (0.90, 3.00)	0.103		1.42 (0.60, 3.37)	0.424
**Wealth status**					
Poorest	ref			ref	
Below average	0.77 (0.33, 1.81)	0.556		0.78 (0.29, 2.05)	0.612
Average	1.28 (0.57, 2.88)	0.551		1.38 (0.54, 3.51)	0.496
Above average	3.00 (1.41, 6.37)	0.004		2.62 (1.02, 6.73)	0.046
Least poor	6.77 (3.16, 14.5)	< 0.001		3.61 (1.27, 10.2)	0.016
**Perceived effect of diseases on herd**					
No effect	ref			ref	
Moderate effect	3.53 (1.36, 9.13)	0.009		3.64 (1.24, 10.7)	0.018
Severe effect	4.45 (1.81, 10.9)	0.001		4.00 (1.51, 10.6)	0.005
**Social support availability**					
Low	ref			ref	
Medium	0.73 (0.41, 1.32)	0.301		1.50 (0.77, 2.90)	0.233
High	2.07 (1.21, 3.52)	0.008		1.83 (0.85, 3.94)	0.123
**Multivariable model evaluation**			**𝒳**^**2**^	***d*ƒ**	***p*-value**
Wald test	*Pseudo R*^*2*^ = 0.22		88.76	16	< 0.001
**Goodness-of-fit test**					
Hosmer – Lemeshow (H–L)			7.75	10	0.463

Variables included as predictors of livestock farmers’ utilization of professional veterinary services in Ghana. Crude odds ratio (cOR) with 95% confidence intervals (CI) and associated *p*-values for the unadjusted model, and adjusted odds ratio (aOR) with 95% CI and associated *p*-values for the adjusted model, accounting for village-level clustering during sampling are reported. ‘ref’ denotes the reference category. 𝒳^2^ are chi-squared statistics of a Wald test comparing the full multivariable model versus the null model, and Hosmer – Lemeshow (H–L) goodness-of-fit test of the model fit with respective degrees of freedom (*d*ƒ) and *p*-values

After adjusting for the farmers’ livestock rearing experience, resilience level, sex, and social support availability, the utilization of professional veterinary services was significantly influenced by distance between farmers and their veterinary personnel, farmers’ herd size, educational attainment, wealth status, and perceived severity of disease effect on herds (Pseudo *R*^*2*^ = 0.22, *p* < 0.001). We evaluated and found that our model was more effective than the null model, and fit the data well (Hosmer – Lemeshow goodness-of-fit 𝒳^2^(10) = 7.75, *p* = 0.46). We did not find evidence that the model assumptions were violated in our post estimation analysis; the residuals scatter randomly, the Pregibon leverage was below the recommended threshold and the predictors were not strongly correlated.

After adjusting for the other predictors, the odds of farmers utilizing professional veterinary services decreased by a factor of 0.91 with each 1-km increase in distance from a service provider (aOR = 0.91, 95% CI = 0.85 – 0.98, *p* = 0.01).

The odds of farmers using professional veterinary services were 2.1 times higher if they had large herd size (5.5 – 182.3 TLUs) (aOR = 2.11, 95% CI = 1.08 – 4.11, *p* = 0.03) compared to if they had small herds (0.3 – 1.8 TLUs). Farmers with basic education were also twice more likely to use services compared to if they had no formal education (aOR = 2.08, 95% CI = 1.07 – 4.06, *p* = 0.03). There was also a three-fold increase in the odds of utilizing the services if the farmer’s wealth status was above average (aOR = 2.62, 95% CI = 1.02 – 6.73, *p* = 0.04) and 3.6 times higher for the least poor households (aOR = 3.61, 95% CI = 1.27 – 10.2, *p* = 0.02), compared to the poorest households. The odds of a farmer utilizing professional veterinary services increases with increasing perception of disease risk to their herd. The odds increased by a factor of 3.6 if farmers perceived diseases effect to be moderate (aOR = 3.64, 95% CI = 1.24 – 10.7, *p* = 0.02), and by a factor of 4.0 if the perceived effect of diseases on the herds was severe (aOR = 4.00, 95% CI = 1.51 – 10.6, *p* = 0.005), compared to when farmers perceive no diseases effect on their herds.

The discussions with farmers similarly showed that, utilization of professional veterinary services was influenced mostly by the affordability of the service, farmer proximity and/or personal access to the veterinary personnel or in cases of complications from diseases or other conditions of the livestock. The professional veterinarians usually are sought after to address the situations that the farmers or informal providers could not successfully handle.

*“When the veterinary officer comes to treat them, we are mostly unable to afford the cost. So, we usually buy the medicines ourselves. So, when we give the animals the injection [treatment] over time, and it gets to a point that it is difficult or beyond us, that is when we call him” ****(Male famer, 49 years old, KAPS District)***

*“My issue is, even when you have money to buy drugs, there is no veterinary post available where you can get the drugs needed. You see, rearing animals also requires a market, but we don’t have a slaughterhouse where you can send the animals to sell. And the veterinary officers here don’t even have any place to store the drugs, ... So, unless, you go and request, and he will give you maybe two weeks, when he would have gone to Accra [Capital of Ghana] and bought the drugs to come and administer. So, by the time he returns with the drugs for you, the sickness would have worsened, ..., you see. And the veterinary officers are also few ****(Male famer, 72 years old, KAPS District).***

*“…for my cattle, I don’t use any medicine [self-treatment]. I’m close to the veterinary officer in the community so whenever there’s any infection of my cattle, I quickly call him to come and treat them for me” ****(Male farmer, 41 years old, Mion District)***

### Appraisal of veterinary services performance

**Fig. 6:**
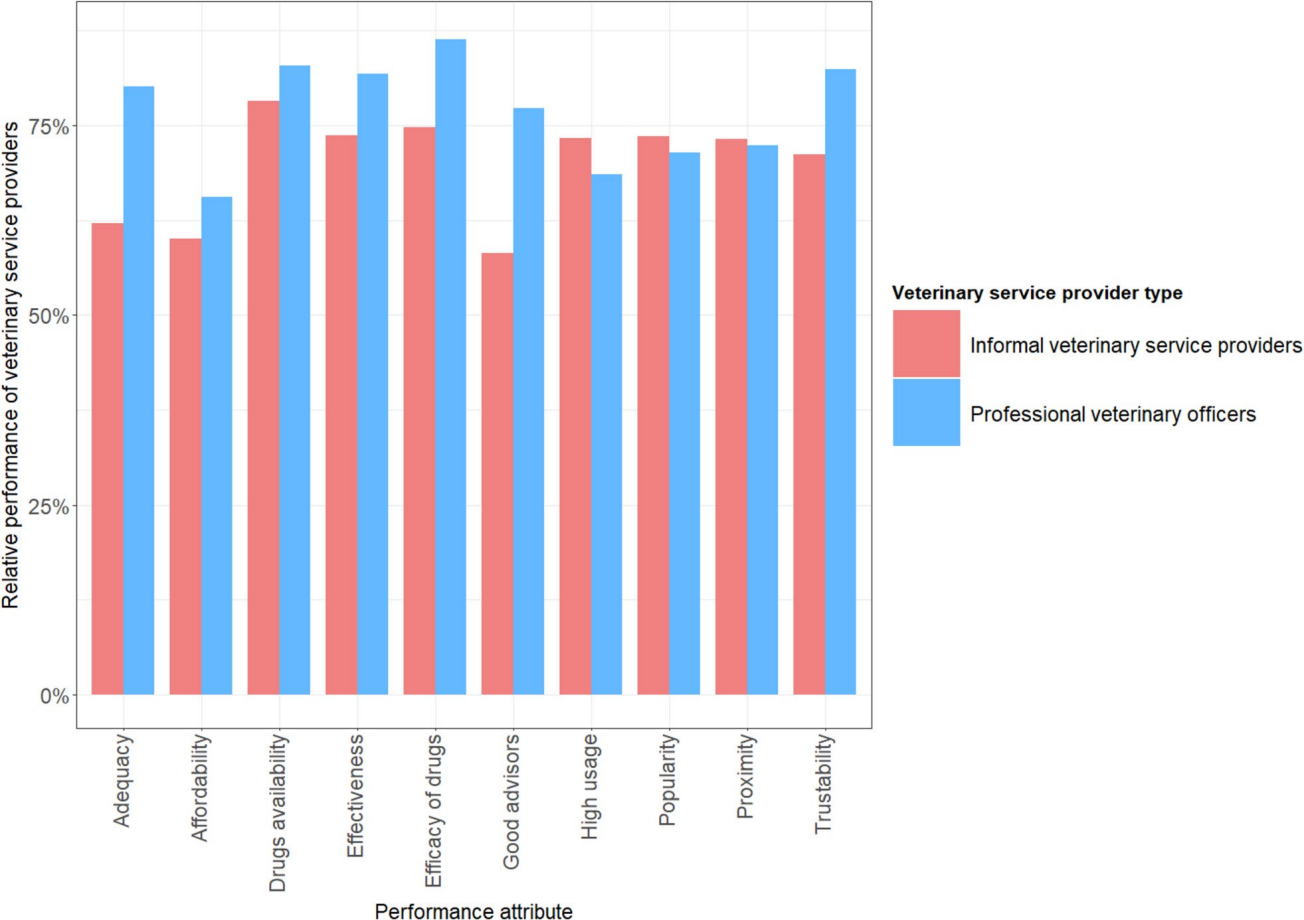
Farmers appraisal of the performance of their veterinary service providers (Fig. 6 presents the appraisal of farmers of the performance of the veterinary service providers that they ever used on 10-item 5-point Likert scale. The y-axis depicts the relative performance of the providers on each attribute. The Relative Importance Index (RII) of each attribute are depicted by the height of the bars, stratified by colors for each provider type)

We assessed the farmers’ general perceptions of the performance of the veterinary service providers they had ever used. Figure 6 compares the relative importance indices (RII) for the performance attributes of VOs and their informal provider counterparts from farmers’ perspective. The VOs performed best on the efficacy of the medicines administered (RII = 0.86) and performed worse on the affordability of services rendered (RII = 0.66). The informal providers performed best on the availability of drugs (RII = 0.78) and worse on the provision of education or advisory services to the farmers (RII = 0.58). Except for the proximity to the farmers, popularity, and high usage of informal providers’ services among the farmers, the VOs were rated highly on all the other attributes. Thus, comparatively, VOs performed better with respect to the availability of medicines when attending to farmers, quality of medicines administered, positive outcome of the treatments administered, provision of health education, affordability of the services rendered, and trust of competence to address the animal health issues in the communities. The informal providers on the other hand were viewed to be closer to the farmers and their services were popular, and highly used by most of the farmers.

The VOs evaluated themselves on the availability of key tools required to deliver their services effectively in a survey. Only 54% (7/13) of the VOs have personnel stationed at border posts to monitor animal movements in their operational areas. About 62% (8/13) reported having slaughter places in their operational areas and having official motorcycles for their work. None of the VOs reported having a designated laboratory to confirm suspected pathogens or any means to control the sale and use of medicines in the respective districts. Most of the VOs (54%) reported that they do not have any form of communication with public health personnel in the districts; 31% (4/13) had informal communications, while only 15% (2/13) had formal communications with public health personnel concerning diseases of zoonotic potential.

In the FGDs with the livestock farmers, we found farmers who had access to the professional veterinarians to be largely satisfied with the services provided to them. The veterinarians provide advice on ways to improve herd health, treat animals, and sometimes purchase medicines for farmers who provide them funds. However, there is generally a dissatisfaction regarding the timeliness of veterinarians’ response to the farmers call for help.

*“What I have noticed is that when he [veterinary officer] comes and once the animal is sick, he does everything possible to help us out. Even if the money to pay is not available at the time he came, he will still consider you. … He considers us a lot” ****(Female farmer, 46 years old, KAPS District)***

*“…the veterinary doctors that attend to us here are very few [3 veterinary officers in KAPS]. So, if you call him [veterinary officer] to come, maybe it is not only you who needs the doctor; another person also calls him today and tomorrow. So, if he goes from one farm to another, by the time he or she would come to you, it would be too late. … But because it is the work you are doing; you need to try to do something to ensure that the animals are healthy by making provision to inject the animal. If you fail to do something, by the time the veterinary doctor comes, the animal will die” ****(Male farmer, 66 years old, KAPS District)***

*“When we call upon the veterinary and they are not able to come on time, we go to town to where they sell the cattle medicines [veterinary drug store]. And when we get there, we describe to them the type of sickness affecting them [livestock], and they give medicines to come and inject the animals” ****(Female farmer, 30 years old, Pru East District)***

## Discussion

In this study, we aimed to identity the main challenges affecting ruminant livestock production in Ghana, evaluate the management strategies applied to deal with diseases and appraise the performance of veterinary services in meeting livestock farmers’ demands for their services. We adopted a convergent parallel mixed-method design to achieve this goal. Our results suggest that animal diseases are the main challenge to livestock production with pestes-des-petits-ruminants (PPR) and mange, and contagious bovine pleuropneumonia (CBPP) and foot-and-mouth disease (FMD), as the main diseases affecting farmers producing small and large ruminants respectively. Farmers mostly utilized treatment services, with the services provided mainly by informal veterinary service providers in the communities, public professional veterinary officers (VOs) or farmers themselves. The choice of management strategies was informed by farmers’ access to VOs, past experiences with the diseases, peer influence, severity of the disease in herd and suggestions by medicine vendors. The antimicrobials mostly used were tetracyclines, penicillins and antiparasitic medicines, although most of the reported applications of these medicines by the farmers were not useful for the conditions or diseases. Overall, farmers were satisfied with the veterinary services provided them, with VOs scoring highly on drugs availability and quality, effectiveness of treatments, education offered, services affordability and community’s trust of their competence to deliver the services compared to the informal providers.

These findings are intuitive, as previous research among sections of the target population similarly identified animal diseases as the primary impediment to the productivity and wellbeing of livestock farming households. Additionally, the previous studies showed that farmers self-treat diseases, and sell diseased animals as coping strategies in response to the lack of adequate veterinary services [11, 26, 36]. The discordance between the perceived effects of the adverse events on livestock production between the farmers and their VOs was logical, given the reported shortfalls in the veterinary workforce and service delivery to livestock farmers in Ghana [12]. Under the current global environment with heightened risks of disease spillovers particularly from animals to humans, the need for the effective control of infectious diseases in animals cannot be overemphasized. Previous reviews have shown vaccination to be both effective and profitable in controlling infectious diseases in animals [37, 38]. With a high prevalence of diseases such as CBPP, FMD and PPR among herds in spite of the availability of effective vaccines, there is a compelling need to identify reasons for the low uptake of vaccination by farmers. Additionally, further studies should also determine livestock farmers’ valuation and willingness to pay for vaccines to protect their livestock against the negative effects of diseases while ensuring sustainability of the disease control interventions.

There is extensive evidence in the literature on the links between increased antimicrobial use in agriculture and emergence of antimicrobial-resistant pathogens [14, 15]. Our results show that in addition to the high rate of antimicrobial use in livestock production in Ghana, including tetracyclines, penicillins and antiparasitic preparations like ivermectin, most application of antimicrobials by farmers were not useful for treating the target diseases. This finding is concerning given the limited capacity of the veterinary services to control the types and quality of antimicrobials that are sold and used in many African countries [13]. The results of our survey with VOs clearly corroborate this limitation in our setting, where the VOs reported the non-availability of mechanisms in the districts to regulate the sale and use of veterinary medicines, in addition to other limitations to control animal movements across operational area borders that facilitate the spread of infectious diseases in animals. In the light of these findings, the risks to public health because of ecosystem pollution with pharmacological preparations as well as the development and spread of antimicrobial-resistant pathogens, are enormous and require urgent strategies to deal with the animal diseases problem in the livestock sector.

Although the VOs were rated highly by farmers who have ever used their services on most of the attributes assessed, they performed poorly in proximity or accessibility to farmers in comparison with the community-based informal veterinary service providers. Thus, we found a high reliance of farmers on their peers and informal veterinary service providers for animal health services. These informal providers, however, operate outside the purview of the formal veterinary system. Moreover, there is a reluctance within the veterinary systems in many African countries to incorporate informal providers in the delivery of veterinary services [39]. Previous studies in other African settings have shown that the deployment of informal providers, known as community-based animal health workers (CAHWs), can complement professional veterinary service providers in delivering animal health services especially in rural areas [27, 40, 41]. Through appropriate training and increased supervision from professional veterinary officers, CAHWs have the potential to function as valuable resources to the veterinary system, working collaboratively with professional veterinary officers in resource-limited settings like Ghana, where there is a substantial shortfall in the veterinary workforce.

Furthermore, we found in our model that utilization of veterinary services was predicted mainly by the social circumstances and human capital of farmers as well as some health system factors. Strategies aimed at improving utilization of veterinary services must focus on these social, human capital, and health system aspects. There is a need therefore for more engagement between policy makers, like the Ministry of Agriculture and Veterinary Services Directorate, and communities towards the development of the veterinary workforce and the co-creation of solutions to address the challenges with animal health services delivery in Ghana. Such solutions must strive for better antimicrobial stewardship in animal production to tackle the emergence of antimicrobial resistant pathogens. Immediate actions to promote effective governance and increased funding for veterinary services are imperative. These measures are indispensable for advancing sustainable livestock production and better animal, human and ecosystem health.

Our study had some limitations. Despite efforts to obtain a representative sample of the different agro-ecological zones in Ghana, our study did not account for the two other minority agro-ecological zones, namely the Evergreen and Coastal Savannah zones. Even though these zones are not typical areas for livestock production in Ghana, it would have been interesting to observe the disease management strategies as well as veterinary services performance in these minority agro-ecological zones. In spite of this missing perspective, we do not expect the parameters evaluated to be markedly different in these agro-ecological zones. Additionally, we relied largely on reported information in our surveys and focus group discussions with study participants. Nevertheless, the triangulation of results from the different methods employed show some validity of our instruments. Our study thus has provided valuable information on the key challenges confronting livestock production, disease management strategies utilized by farmers and appraisal of veterinary services performance in Ghana. Additionally, interviewing the informal providers in the veterinary system would have provided a better understanding of their activities and role in the animal health service delivery. However, this was not covered within the scope of this study. Future studies should address the role of informal providers in the veterinary system from their perspective.

## Conclusion

Our study shows that animal diseases including PPR and Mange in small ruminants, and CBPP and FMD in cattle remain a key bottleneck to the productivity of livestock and wellbeing of livestock dependent populations in Ghana. Disease management strategies adopted by farmers are influenced mainly by accessibility to professional veterinarians, severity of diseases on herds, peer influence, experience with diseases and suggestions by drug vendors. The antimicrobials applied in the treatment for most of the animal diseases and conditions by the farmers are not useful. Although the farmers are largely satisfied with the performance of their professional veterinary service providers in terms of drugs availability and quality, effectiveness of treatments, health education, service affordability and competence to deliver veterinary services, informal veterinary service providers are widely used due to their proximity to farmers in the communities. Given that the main diseases reported have available effective vaccines for their control and vaccination utilization is low among the farmers, our findings underscore the urgent need to improve the adoption and use of vaccination services by farmers, as well as better antimicrobial stewardship and veterinary services governance to properly regulate the animal health service delivery in Ghana.

### Supplementary Information

**Additional file 1. **Survey instrument for livestock farmers' survey.

**Additional file 2. **Survey instrument for veterinary service providers' survey.

**Additional file 3. **Samples of different types of medicines used by livestock farmers in Ghana.

## Data Availability

All data generated or analyzed during this study are included in this published article [and its supplementary information files].
