# Supplementary material for: Management of diseases in a ruminant livestock production system: a participatory appraisal of the performance of veterinary services delivery, and utilization in Ghana
Source: BMC Vet Res. 2023 Nov 15;19:237. doi: 10.1186/s12917-023-03793-z (PMC10647120; doi:10.1186/s12917-023-03793-z)

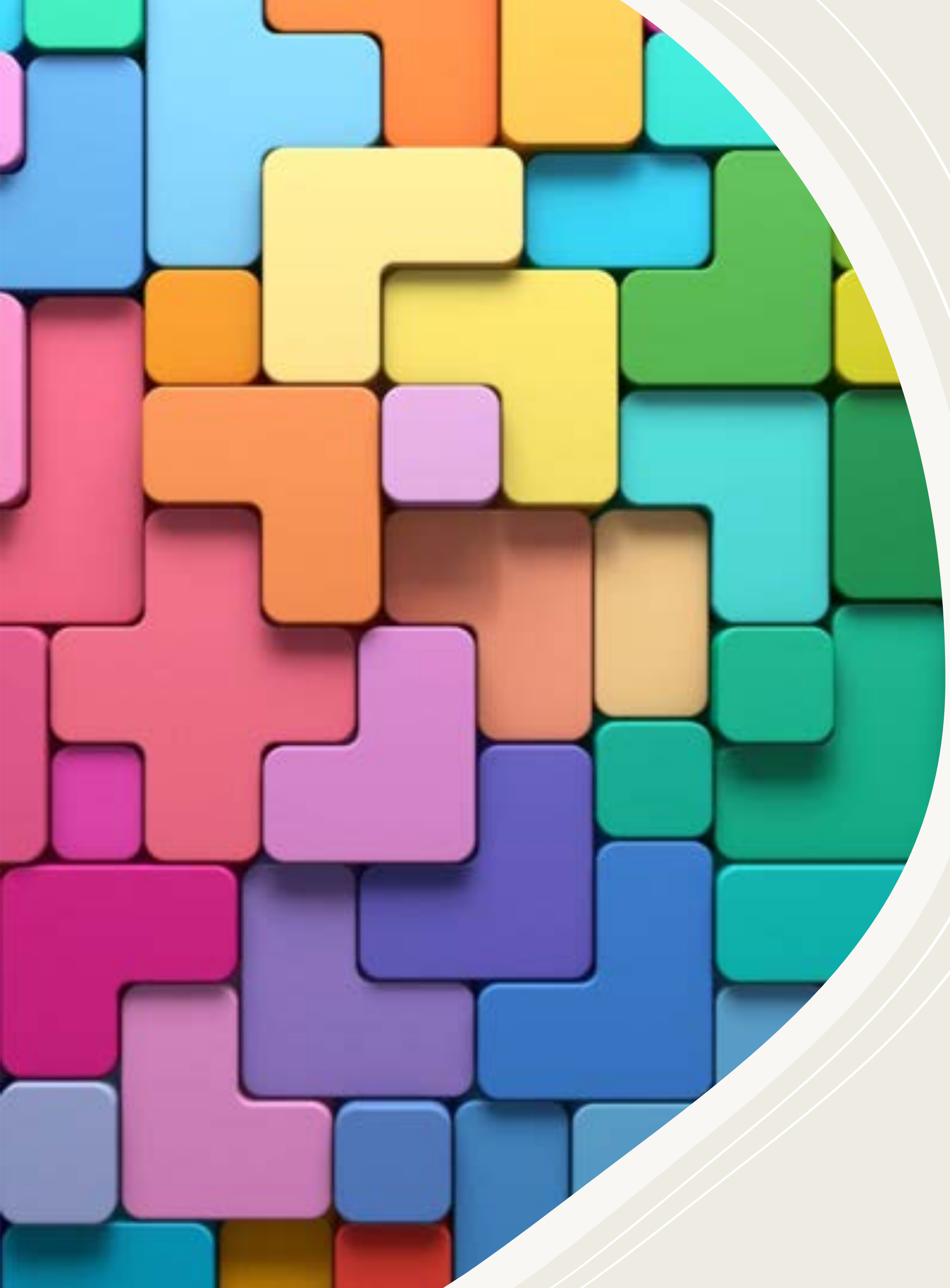

Additional file 3: Samples of different types of medicines used by livestock farmers in Ghana

Source: Field pictures captured by 1st author

TETRACYCLINES 1/6

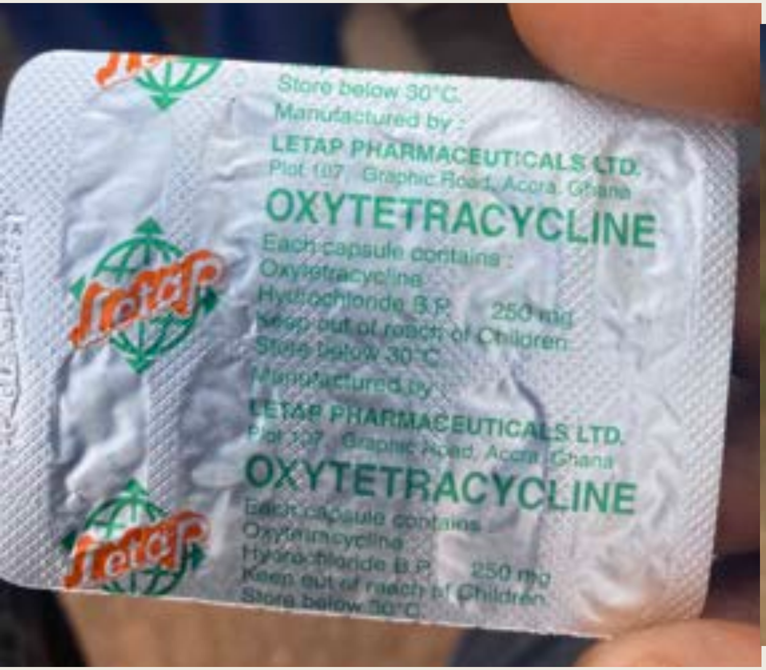

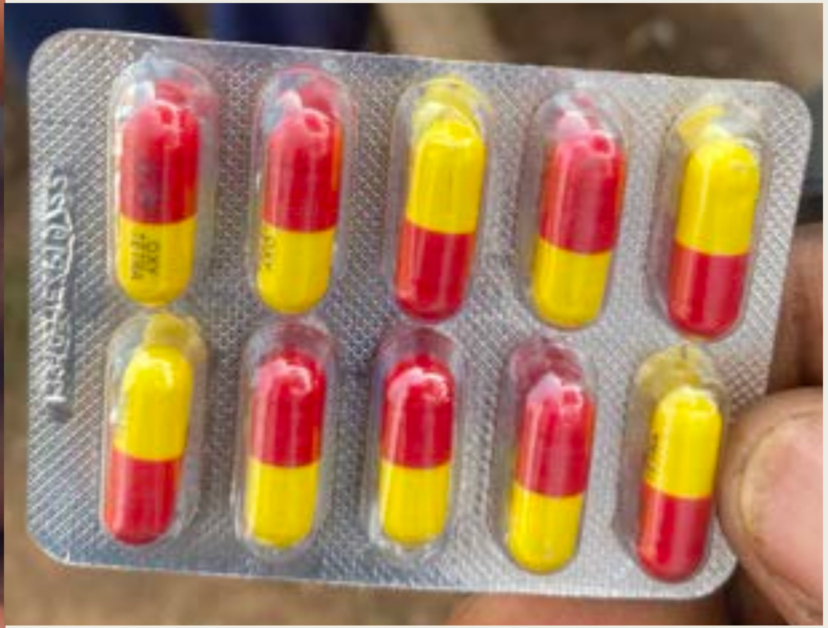

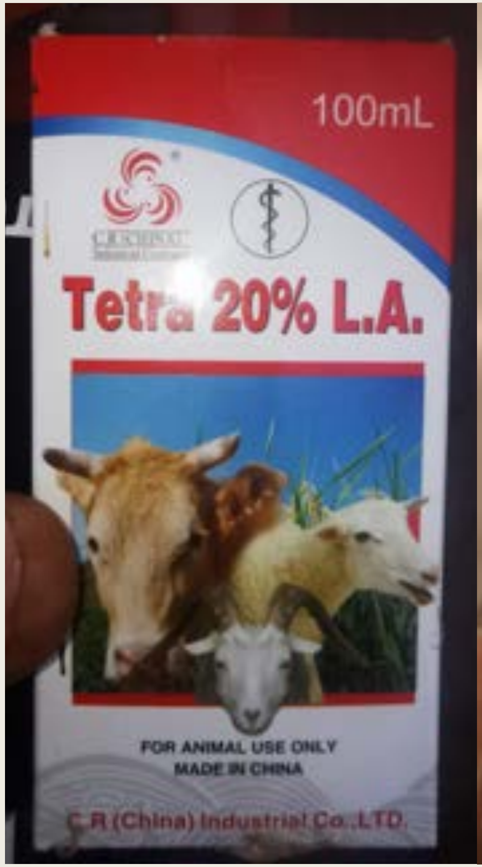

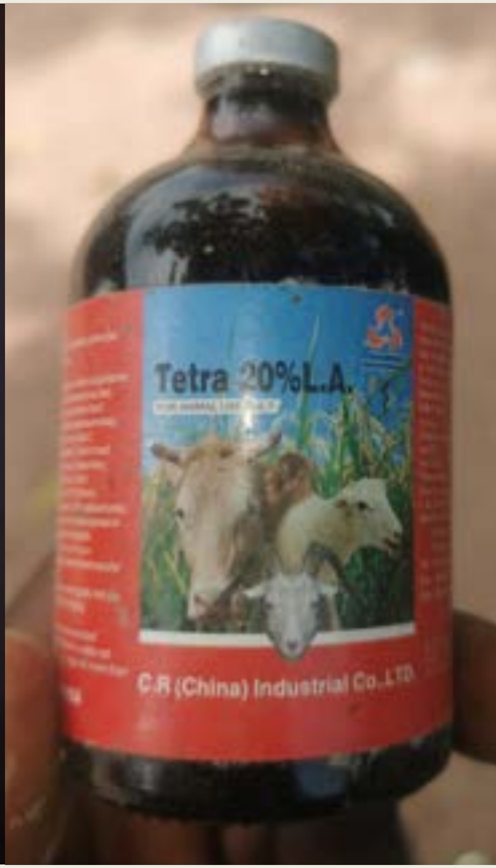

TETRACYCLINES 2/6

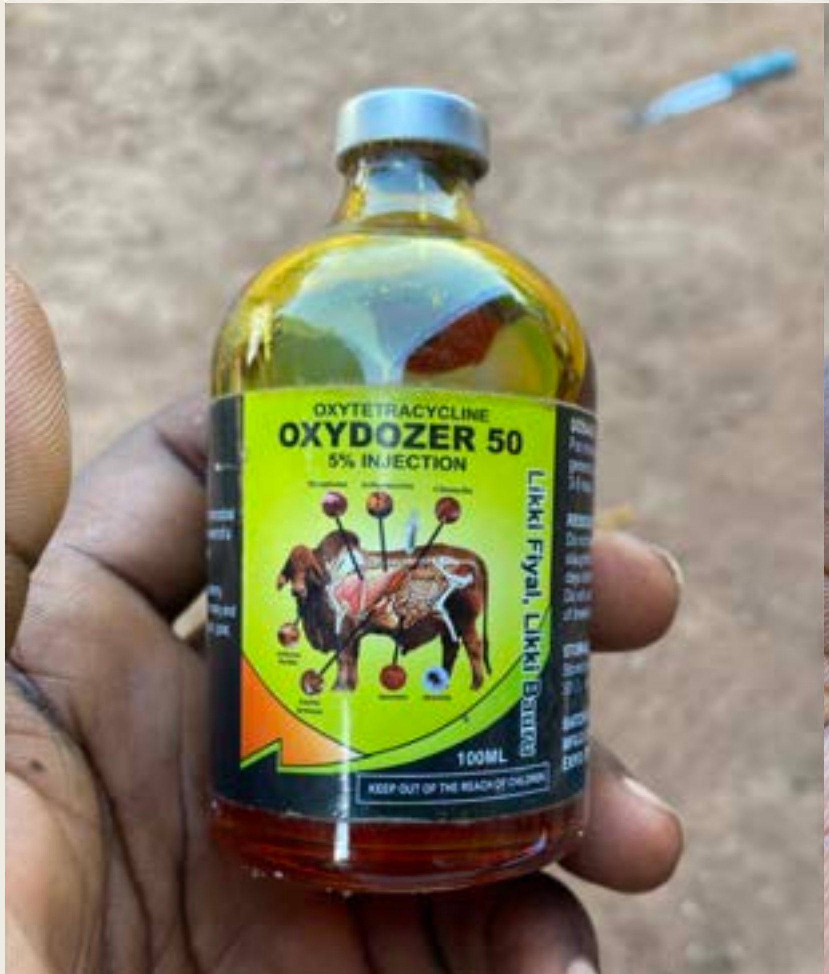

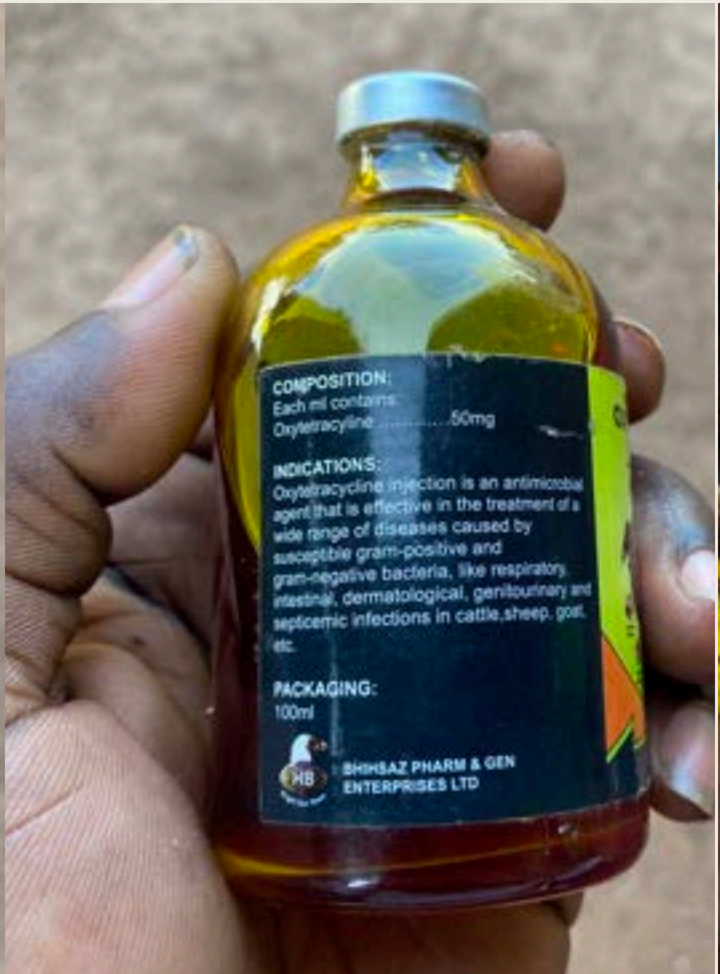

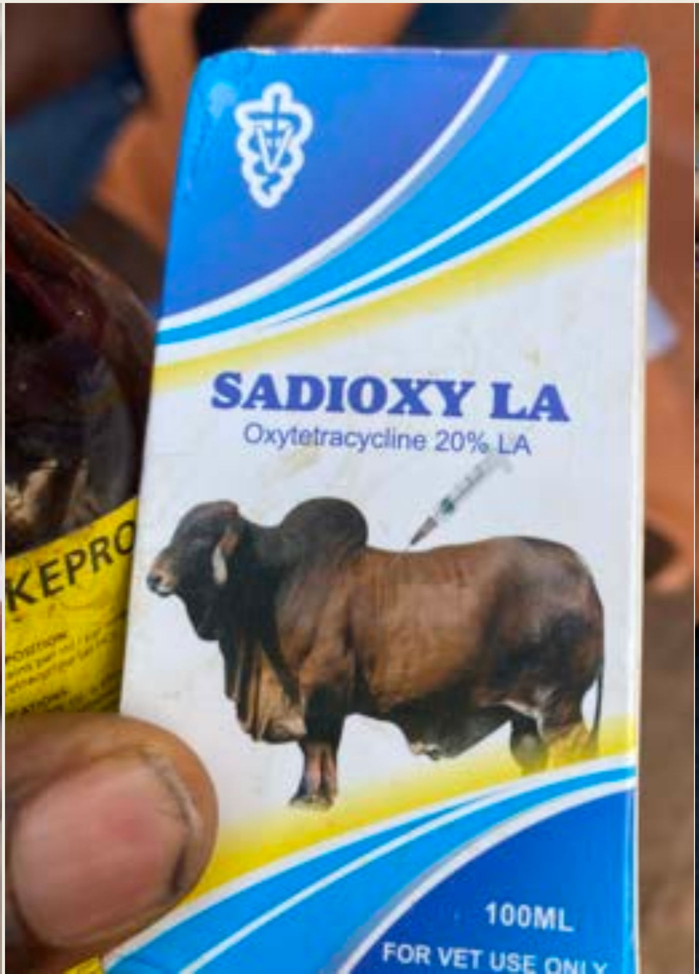

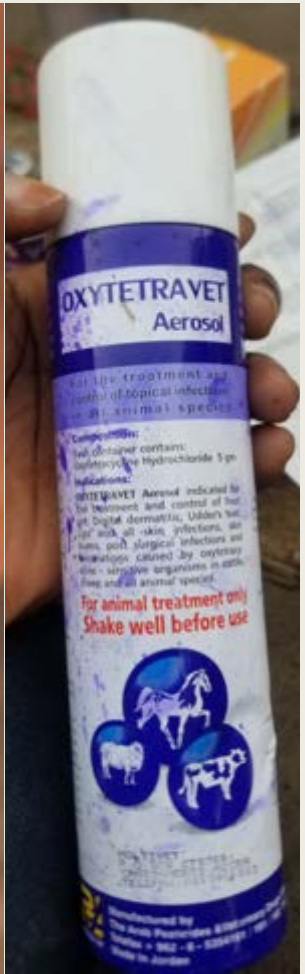

TETRACYCLINES 3/6

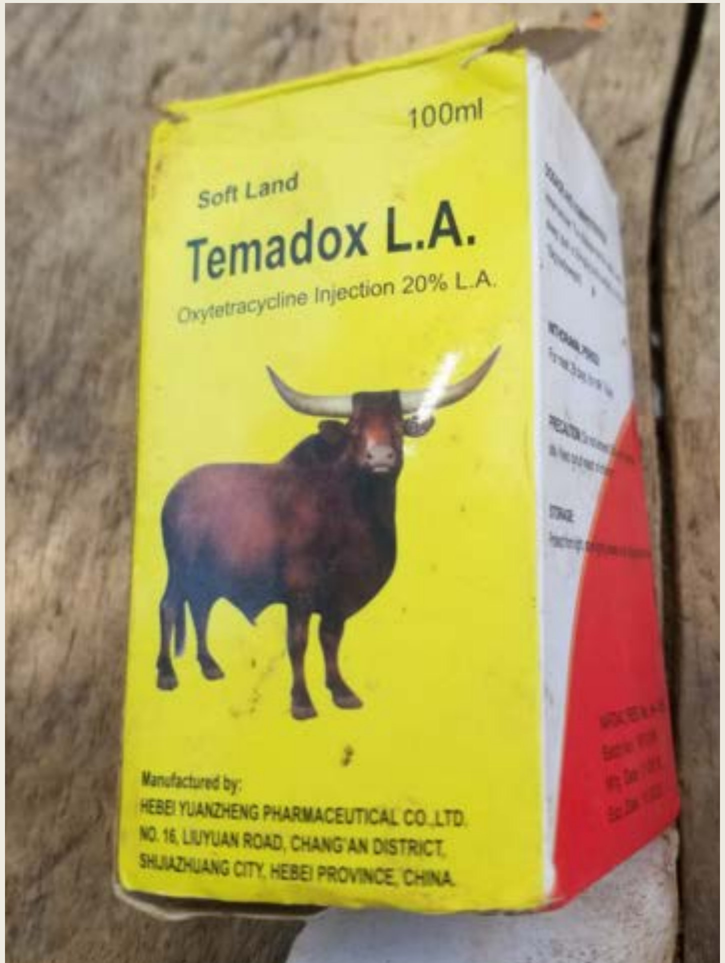

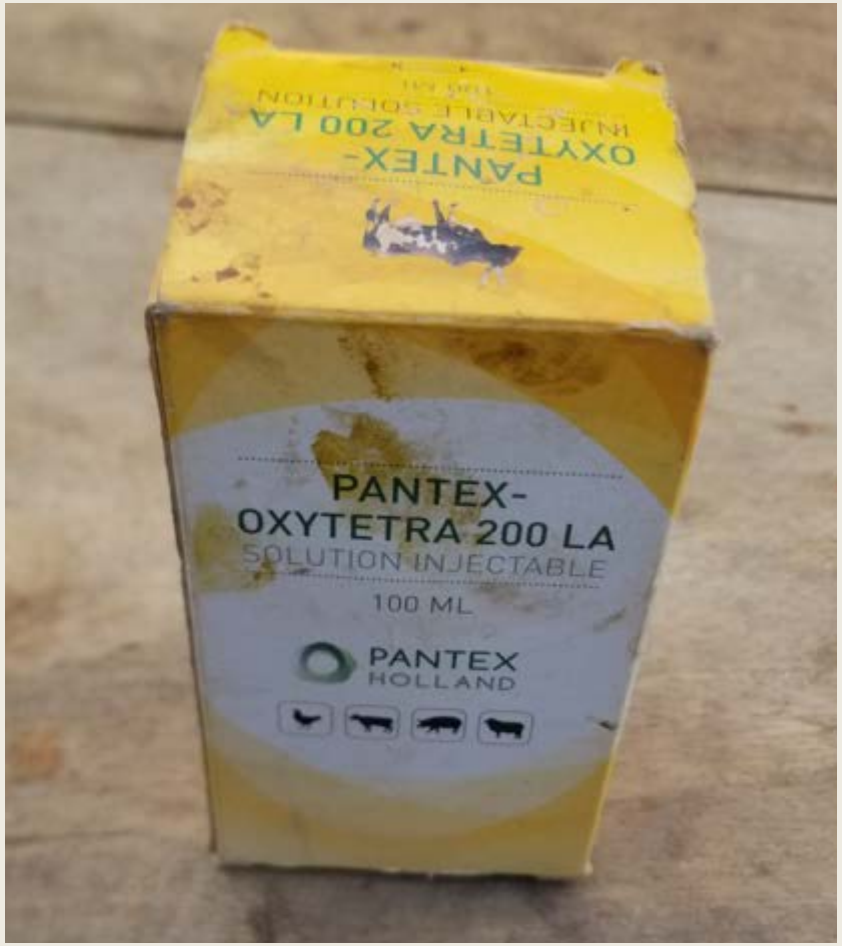

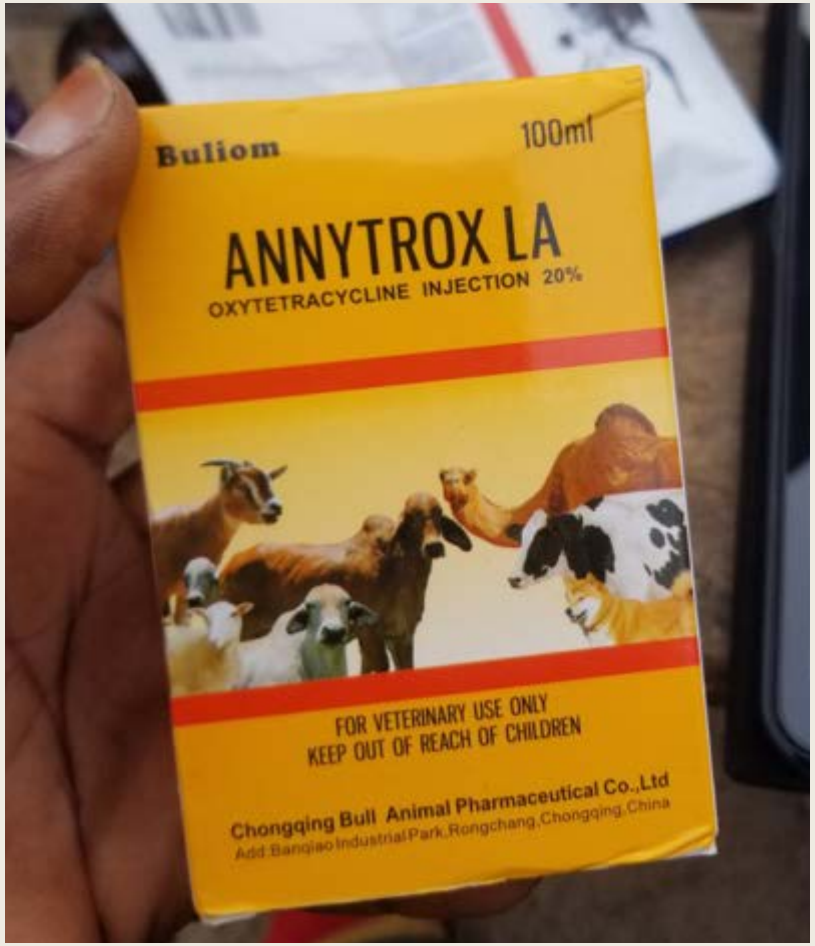

TETRACYCLINES 4/6

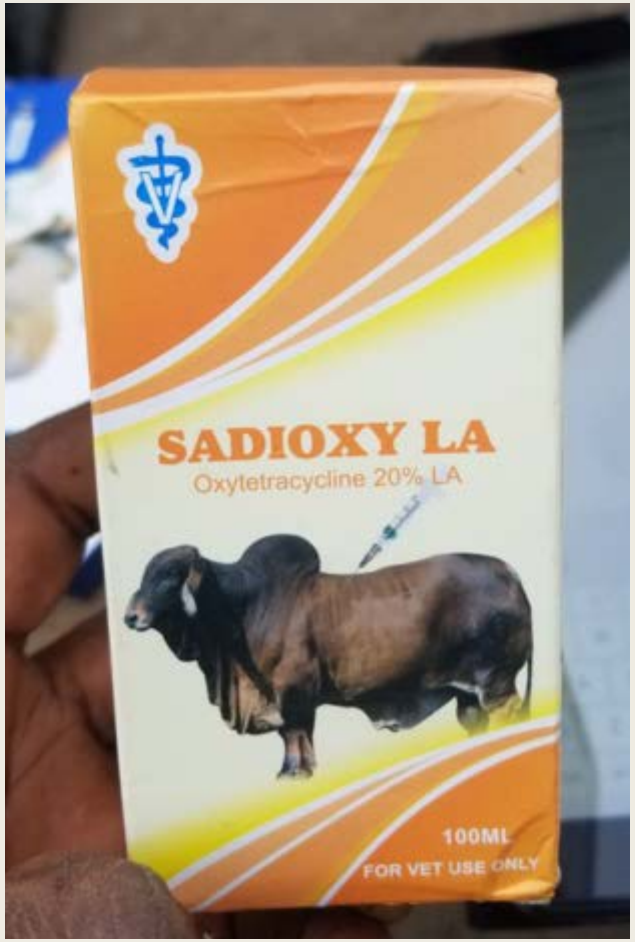

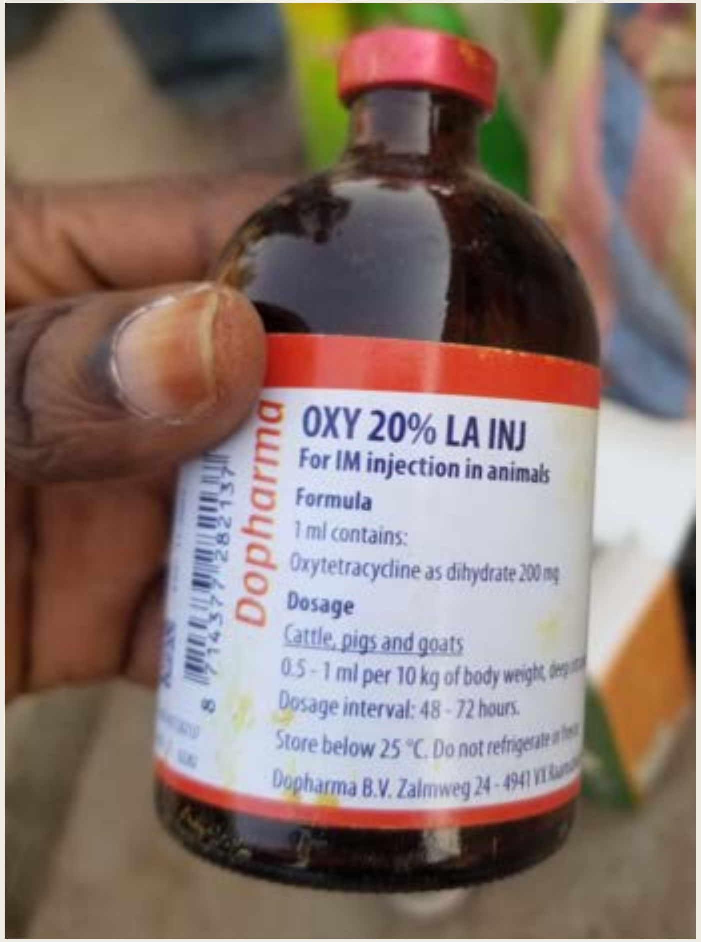

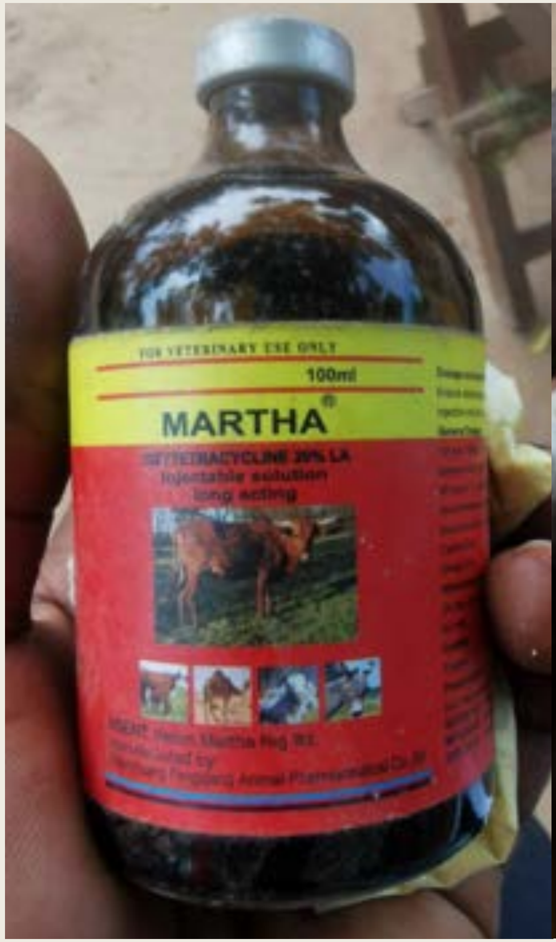

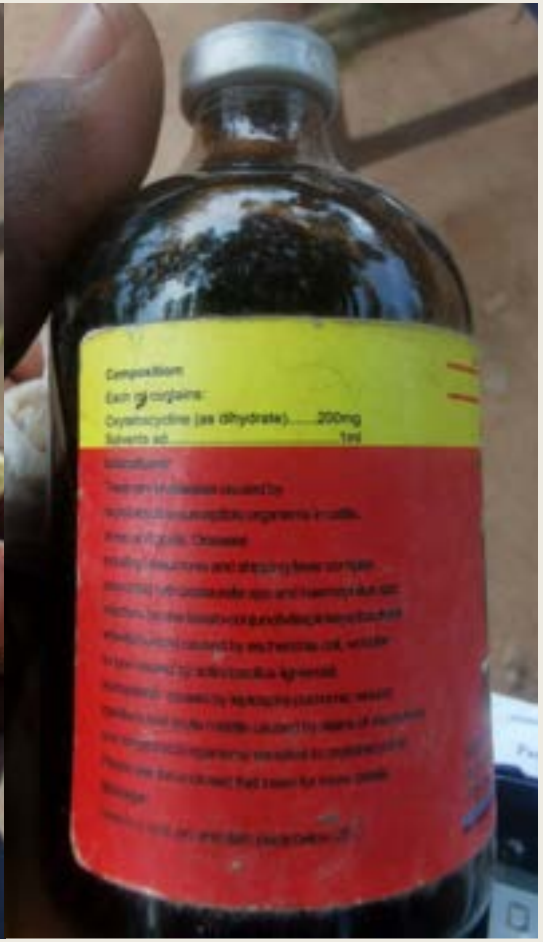

TETRACYCLINES 5/6

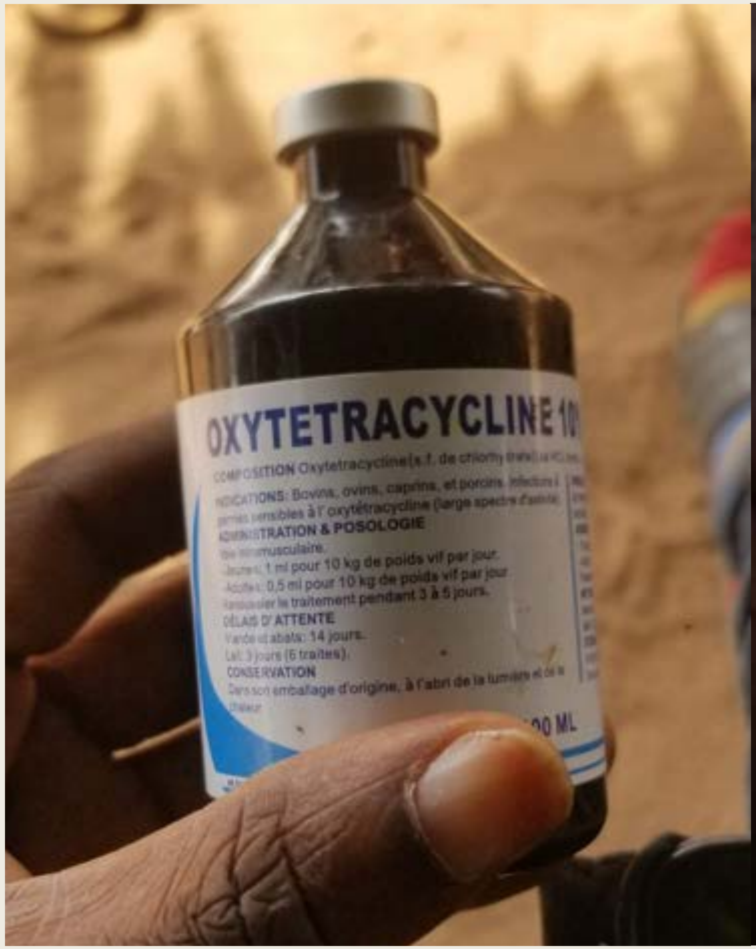

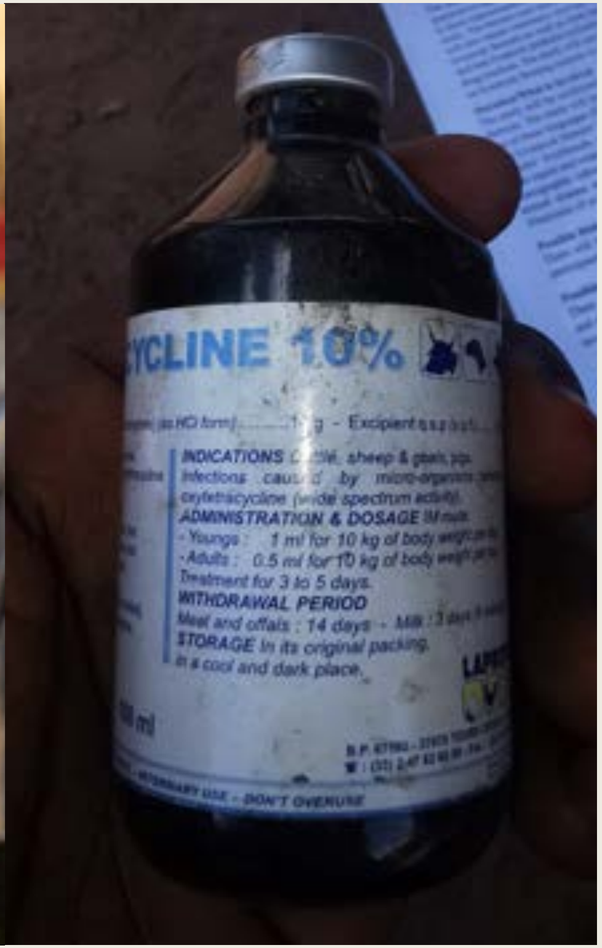

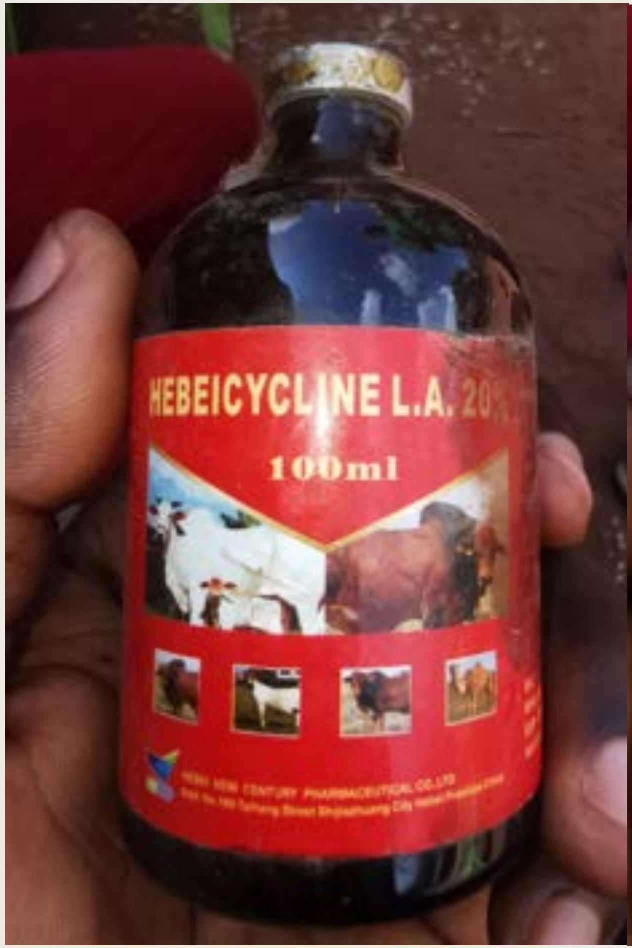

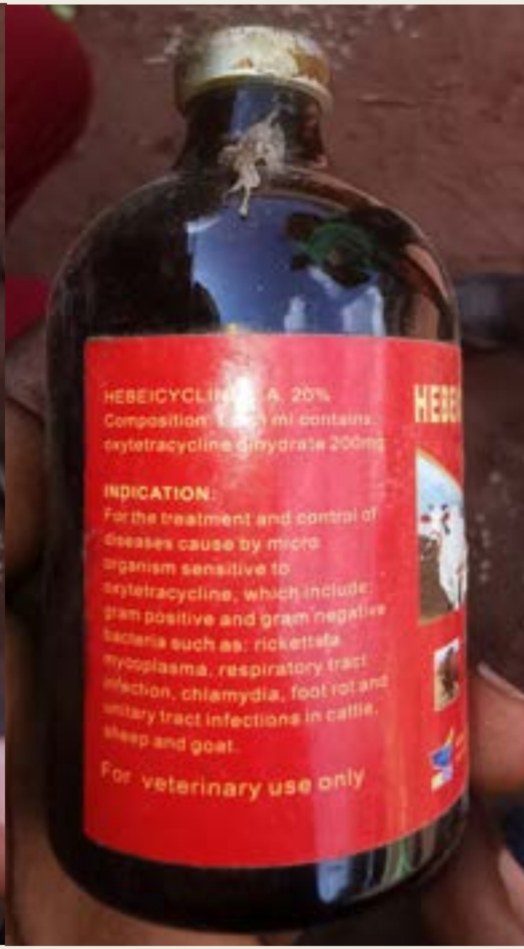

TETRACYCLINES 6/6

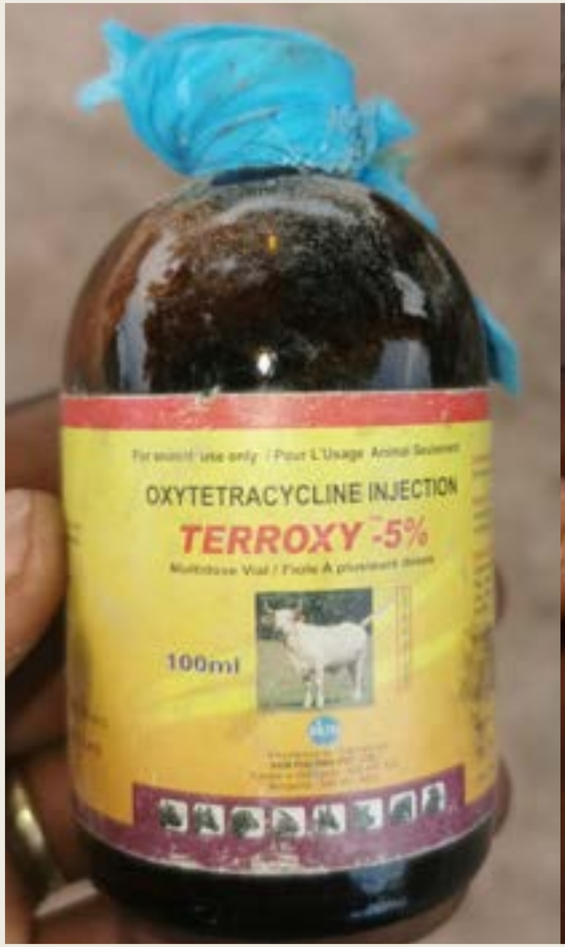

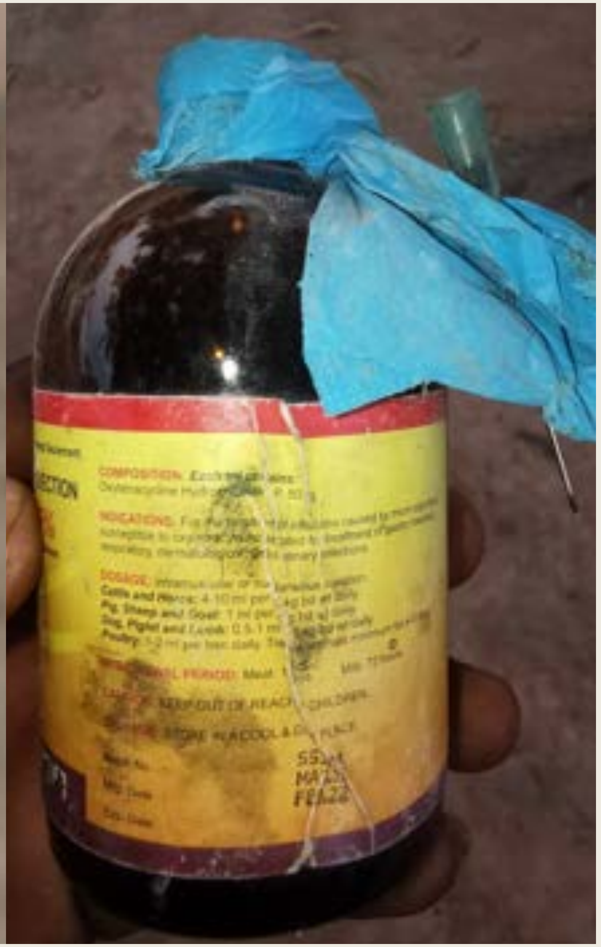

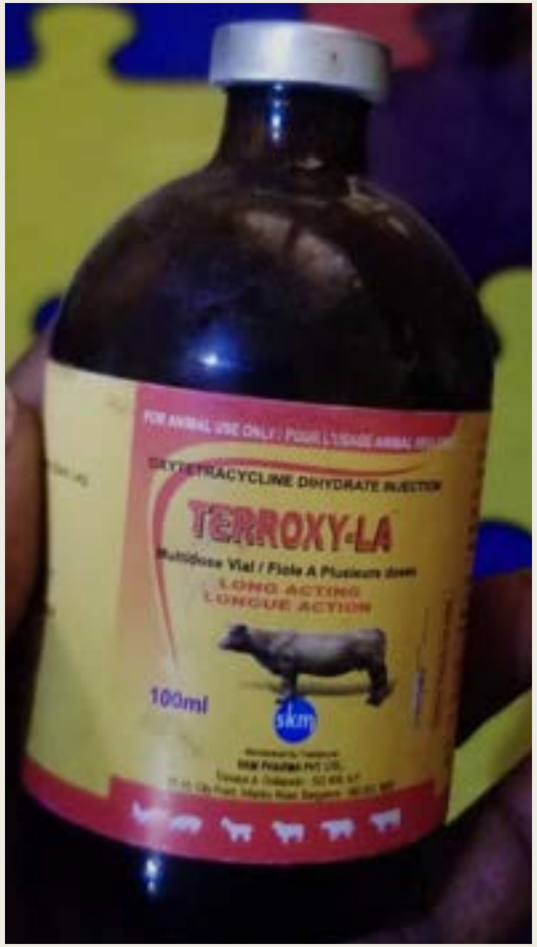

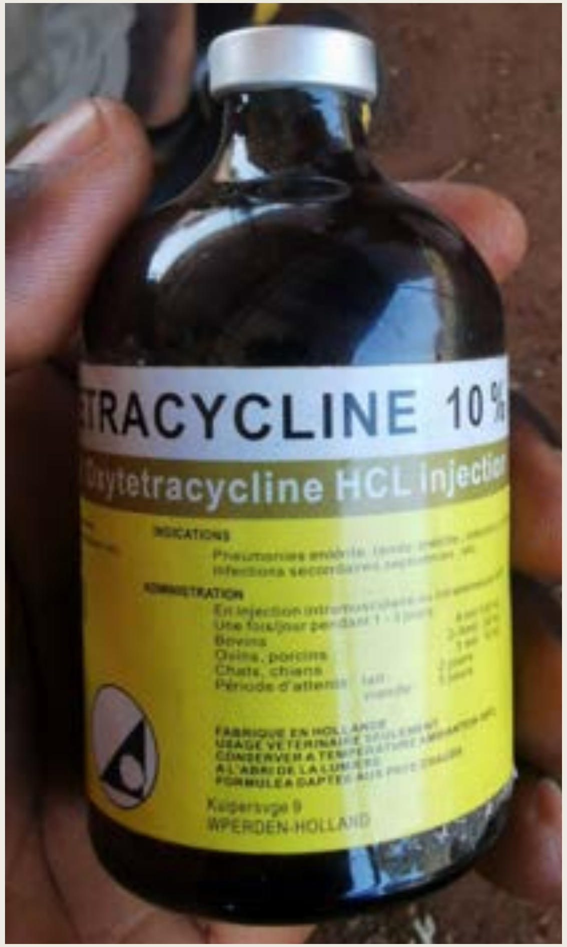

PENICILLINS 1/2

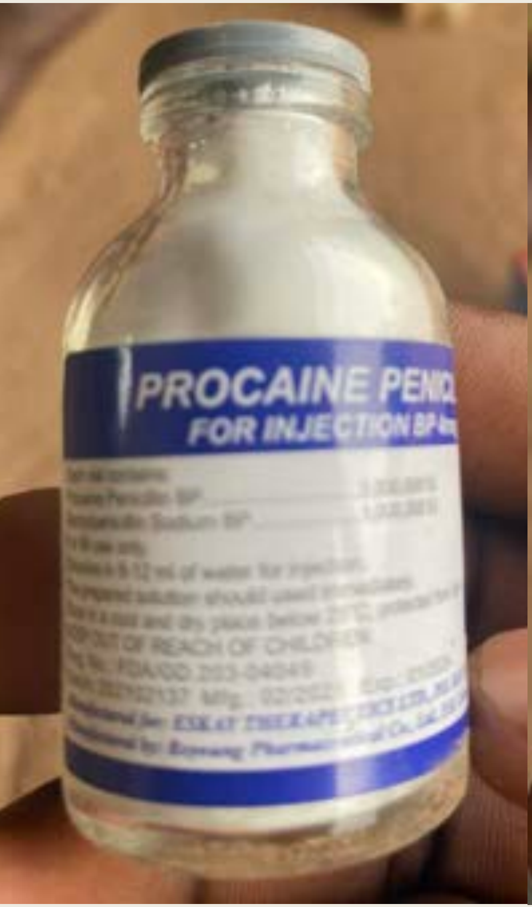

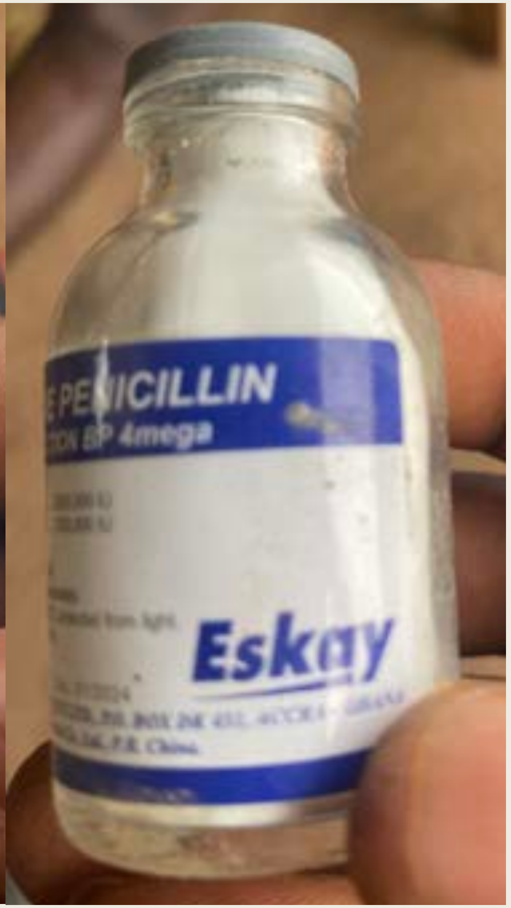

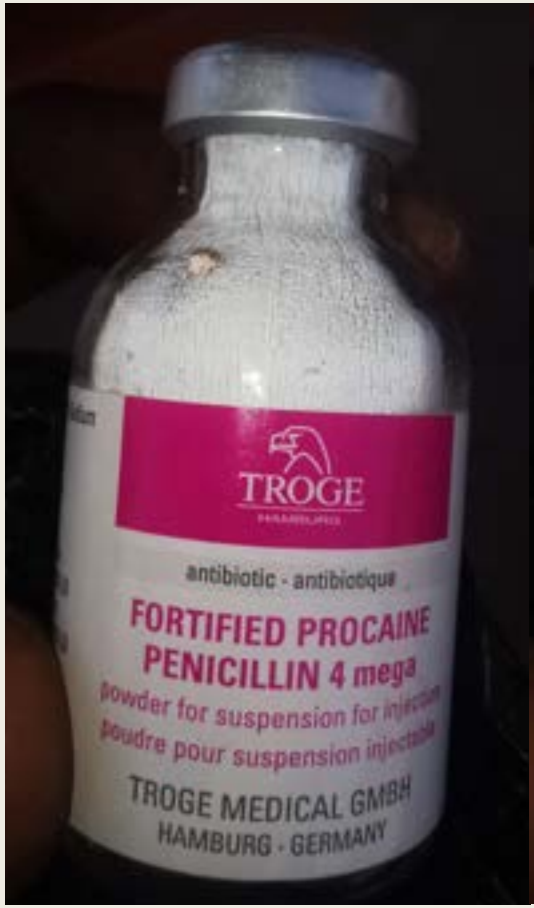

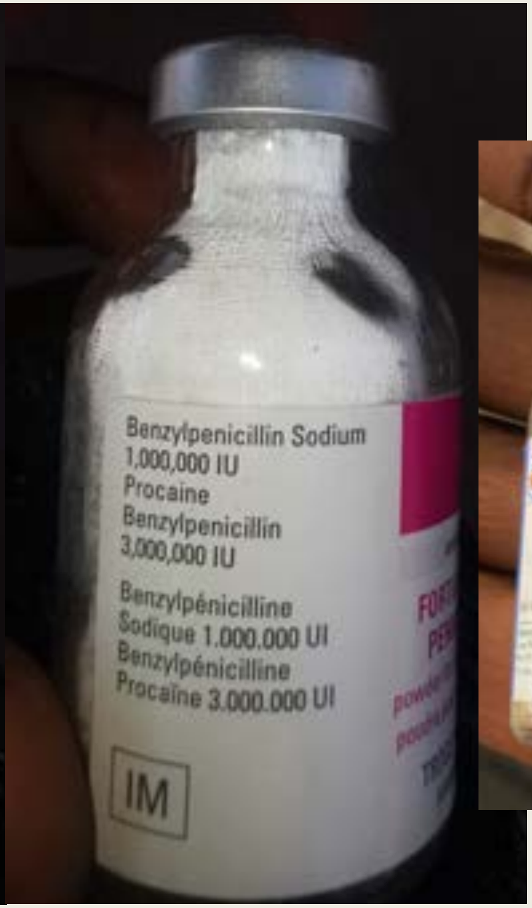

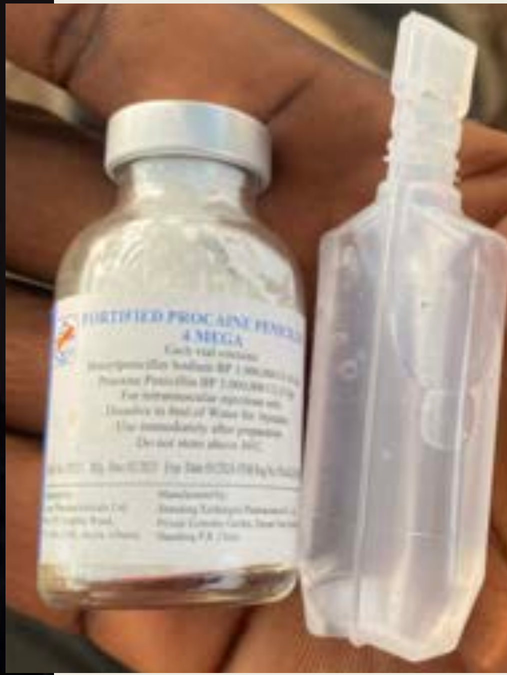

PENICILLINS 2/2

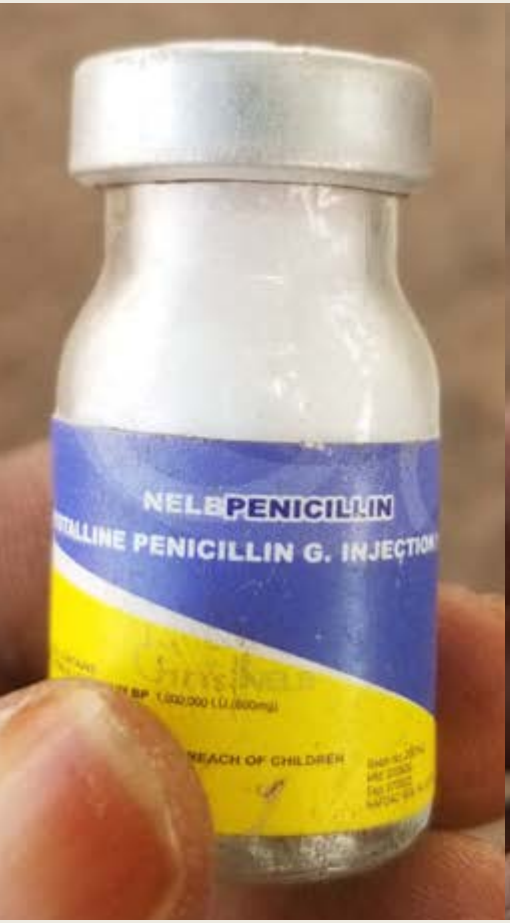

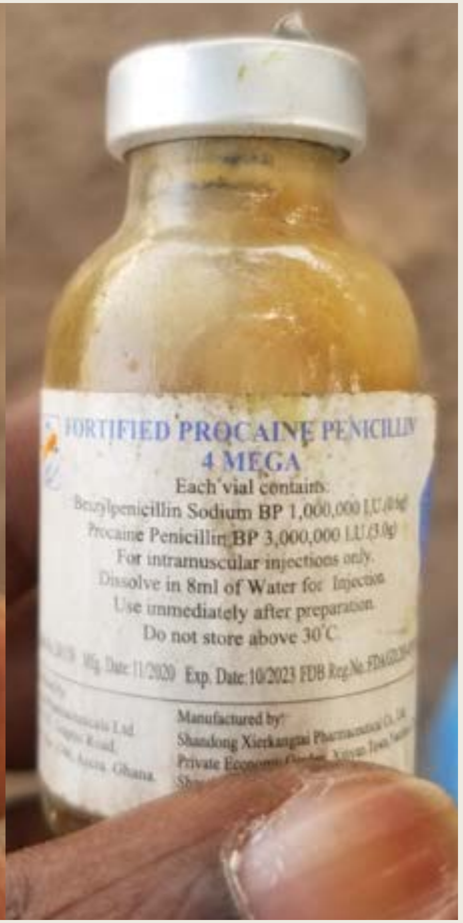

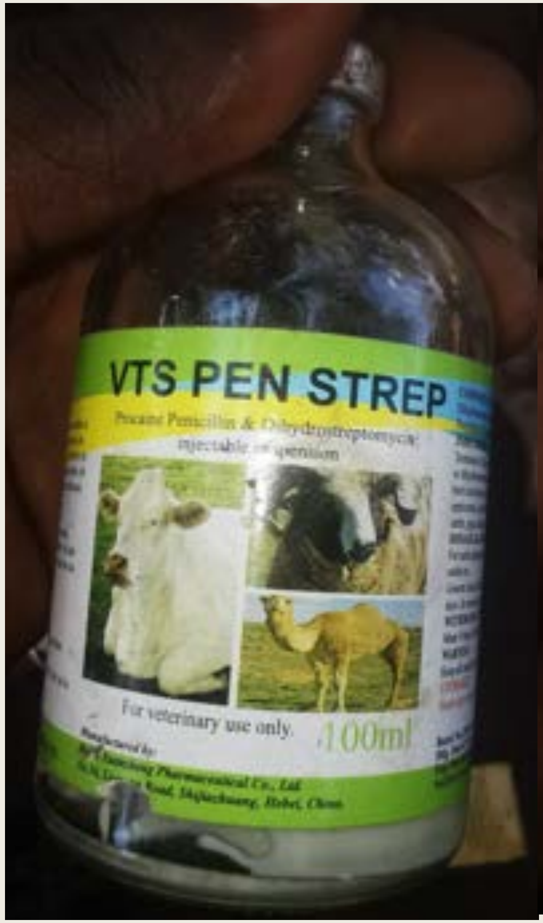

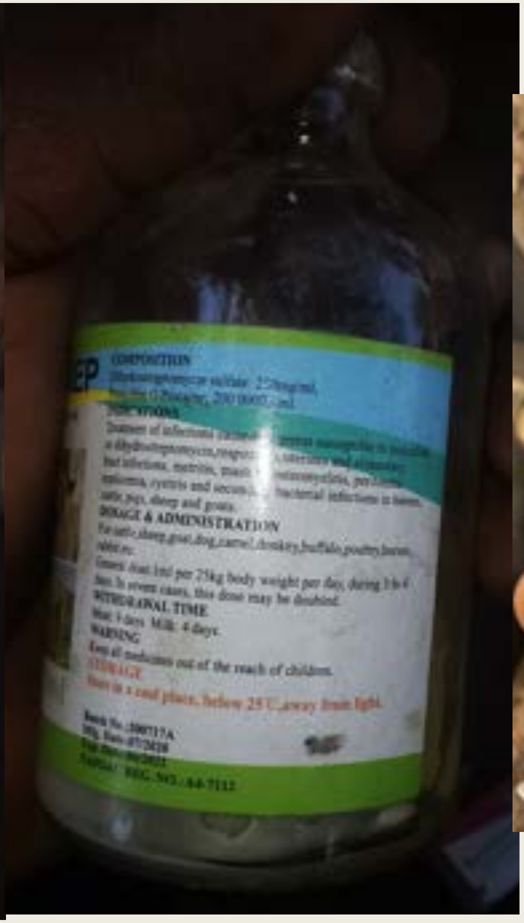

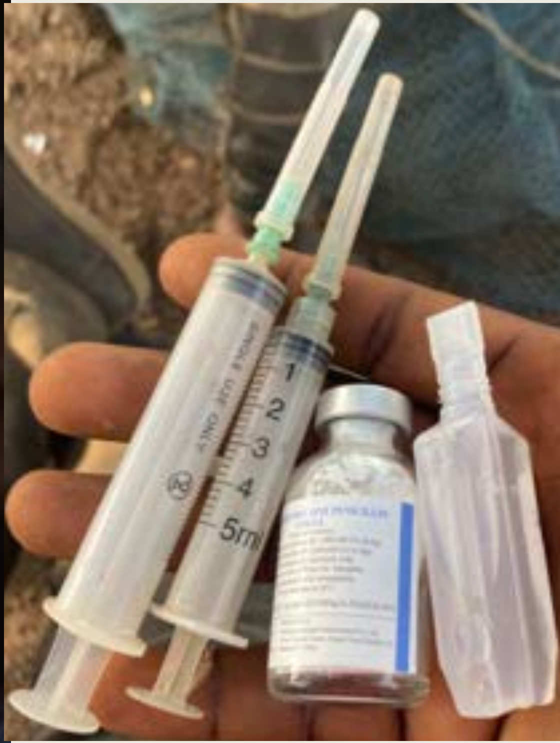

TYLOSIN 1/2

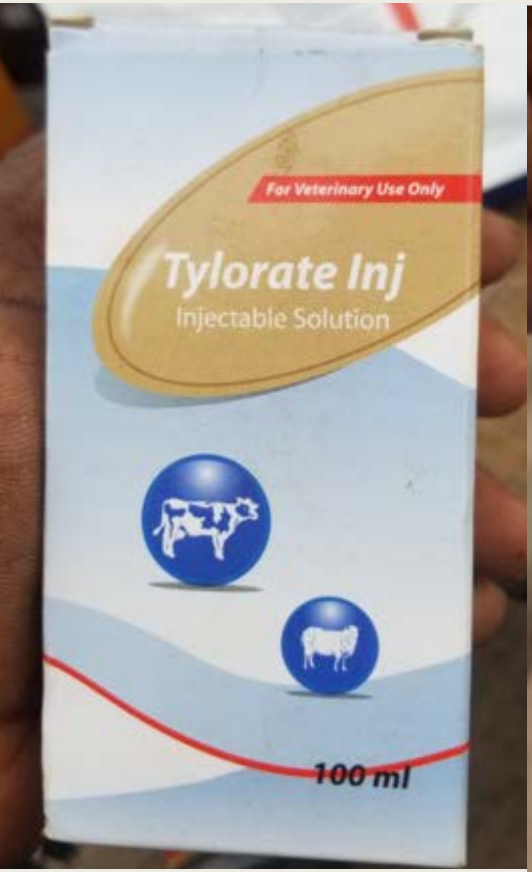
For Veterinary Use Only
Tylorate Inj
Injectable Solution
100 ml

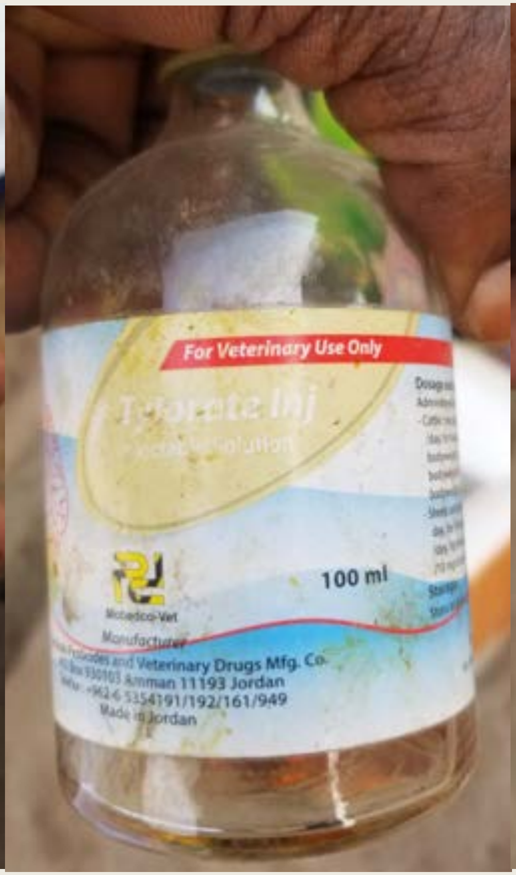
For Veterinary Use Only
100 ml
Manufacturer
Amman 11193 Jordan
Made in Jordan

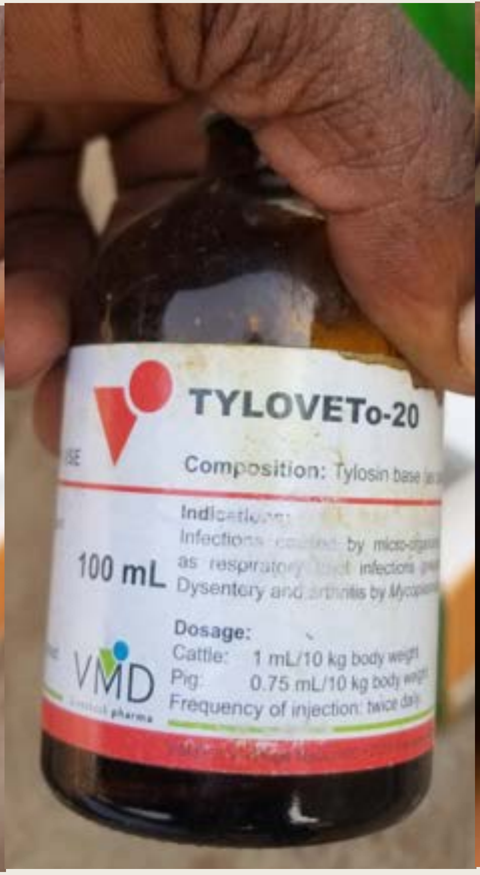
TYLOVETo-20
Composition: Tylosin base
100 mL
Dosage:
Cattle: 1 mL/10 kg body weight
Pig: 0.75 mL/10 kg body weight
VMD

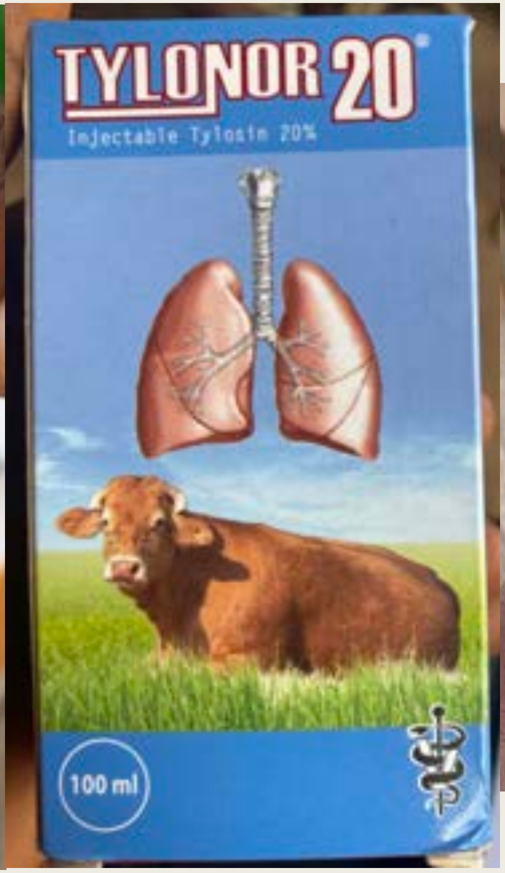
TYLONOR 20
Injectable Tylosin 20%
100 ml

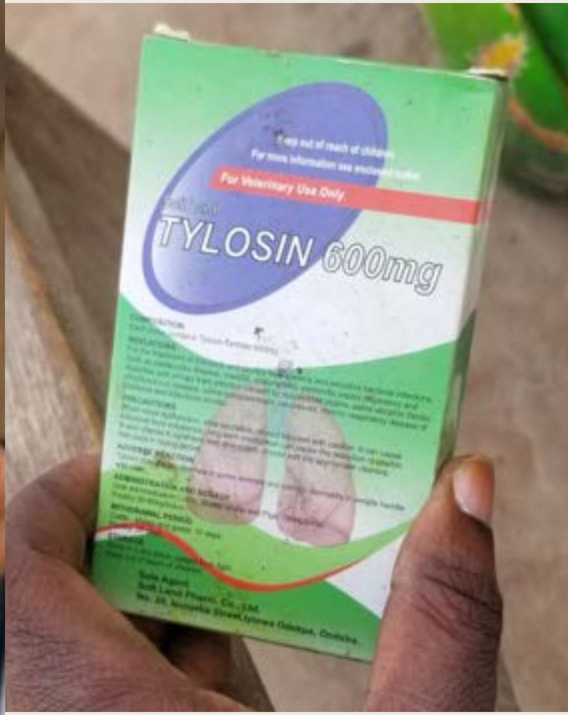
For Veterinary Use Only
TYLOSIN 600mg

TYLOSIN 2/2

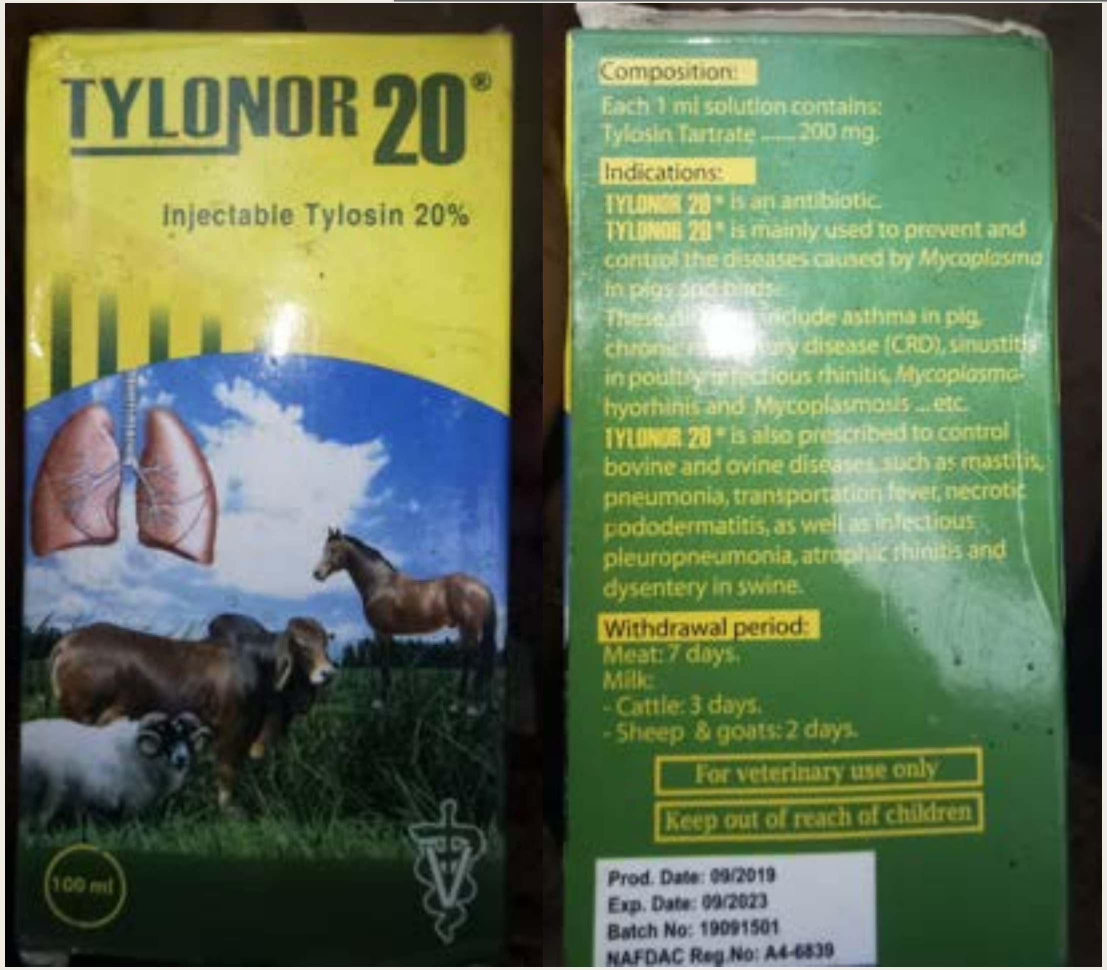
TYLONOR 20®
Injectable Tylosin 20%
100 ml
Composition:
Each 1 ml solution contains:
Tylosin Tartrate .... 200 mg.
Indications:
TYLONOR 20® is an antibiotic.
TYLONOR 20® is mainly used to prevent and control the diseases caused by Mycoplasma in pigs and birds.
These diseases include asthma in pig, chronic respiratory disease (CRD), sinusitis in poultry, infectious rhinitis, Mycoplasma hyorhinis and Mycoplasmosis ... etc.
TYLONOR 20® is also prescribed to control bovine and ovine diseases such as mastitis, pneumonia, transportation fever, necrotic pododermatitis, as well as infectious pleuropneumonia, atrophic rhinitis and dysentery in swine.
Withdrawal period:
Meat: 7 days.
Milk:
- Cattle: 3 days.
- Sheep & goats: 2 days.
For veterinary use only
Keep out of reach of children
Prod. Date: 09/2019
Exp. Date: 09/2023
Batch No: 19091501
NAFDAC Reg.No: A4-6839

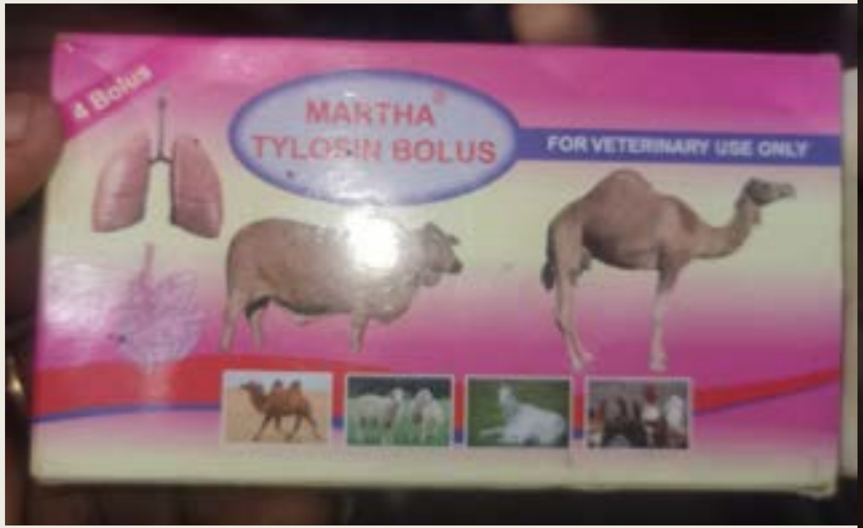
4 Bolus
MARTHA®
TYLOSIN BOLUS
FOR VETERINARY USE ONLY

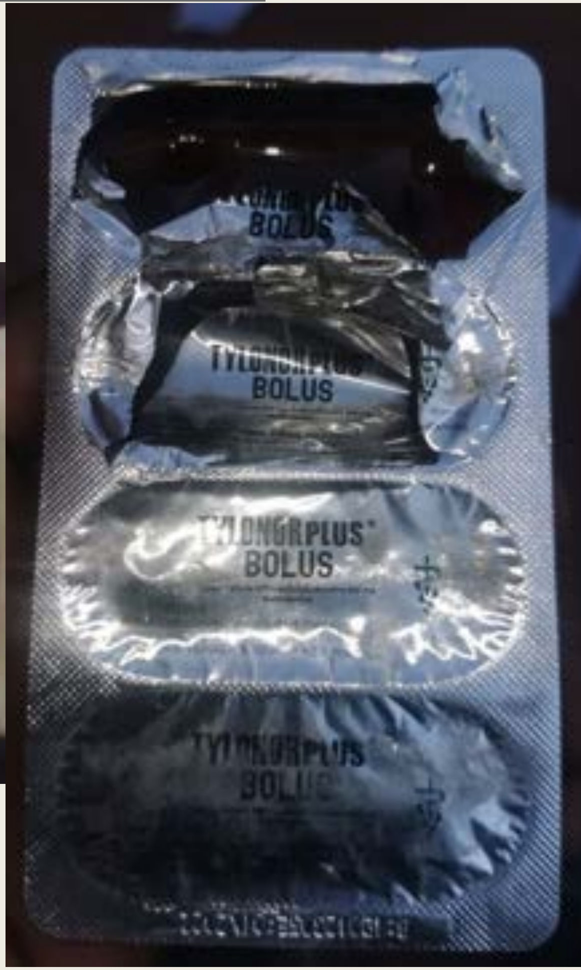
TYLONORPLUS®
BOLUS

OTHER ANTIBIOTICS

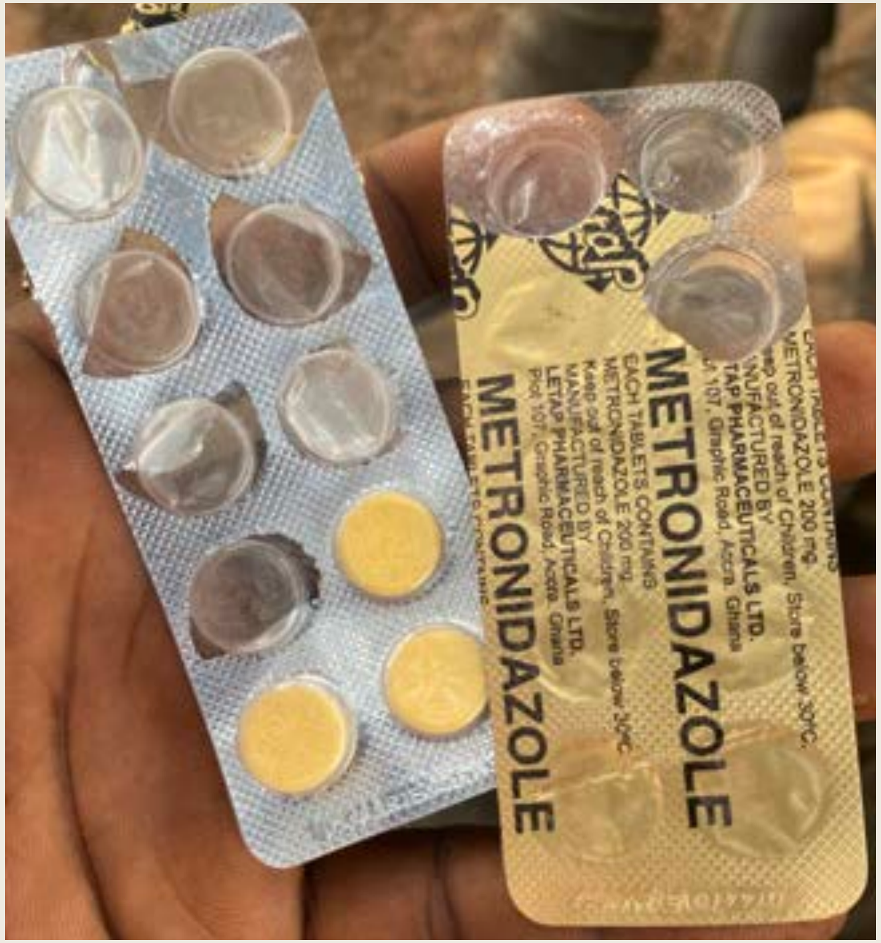

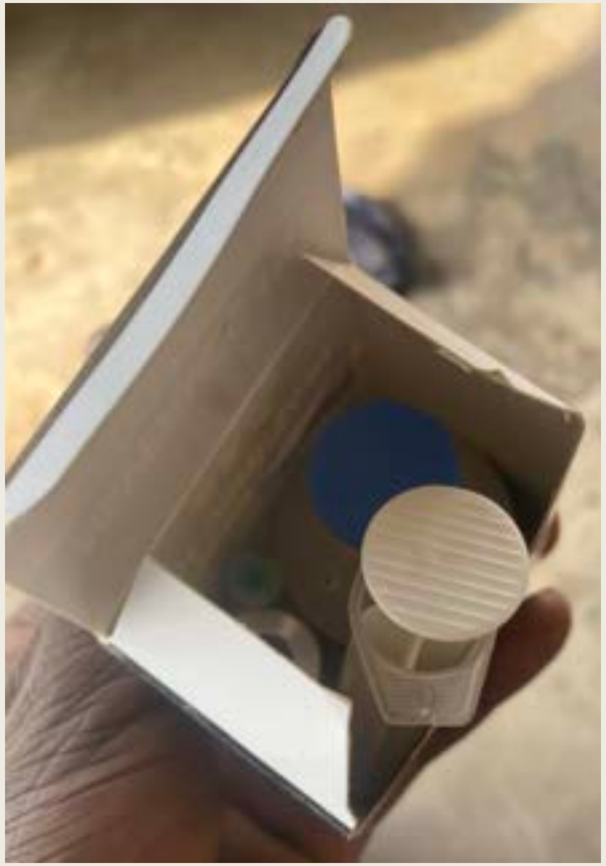

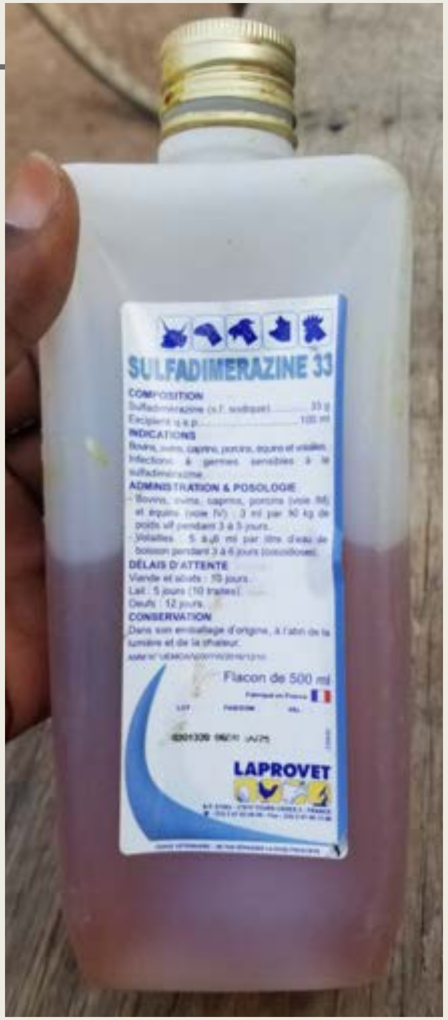

ANTIPARASITIC MEDICINES 1/6

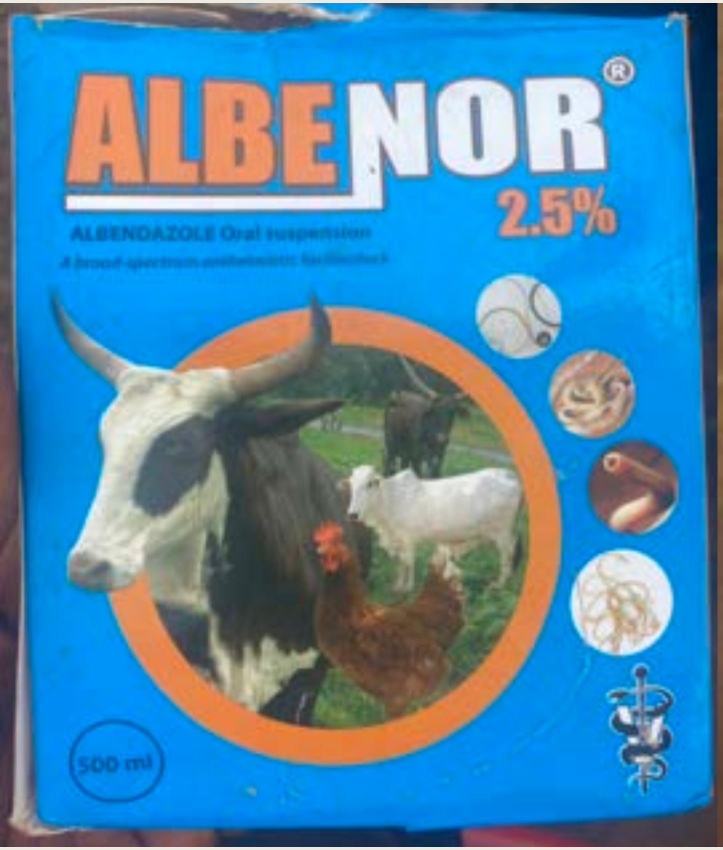

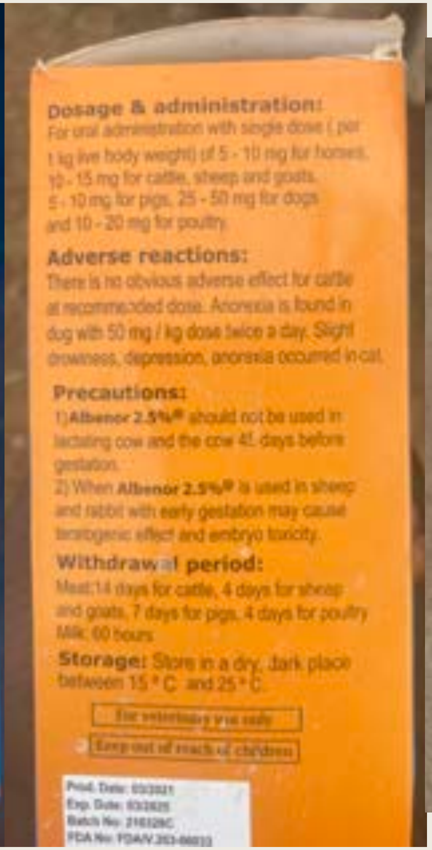

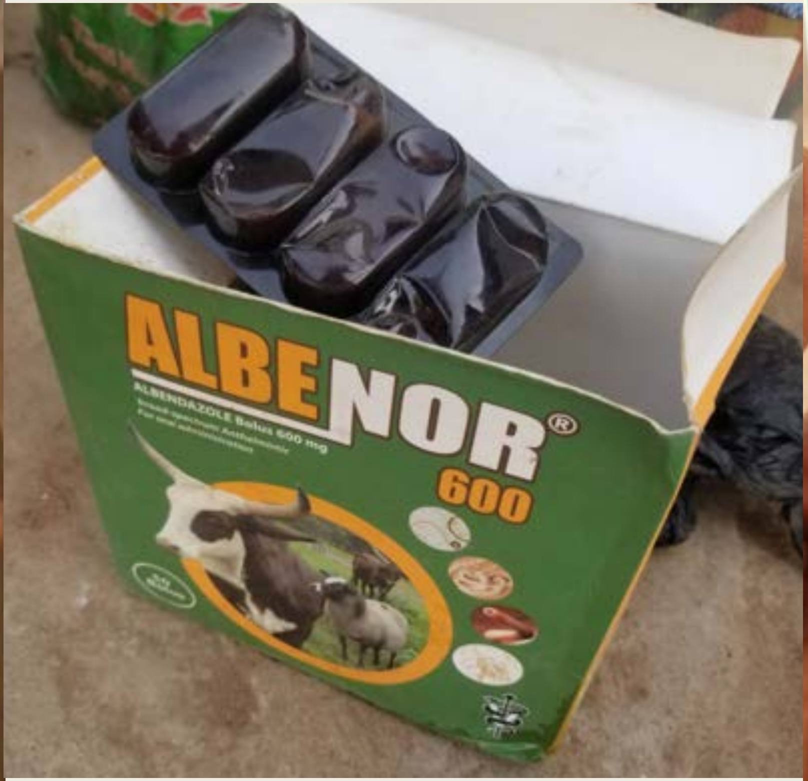

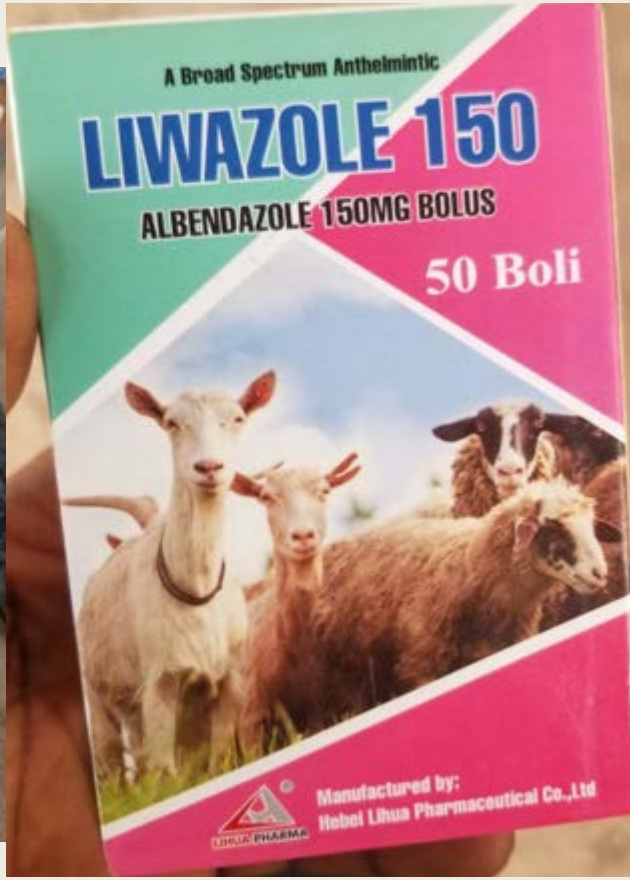

ANTIPARASITIC MEDICINES 2/6

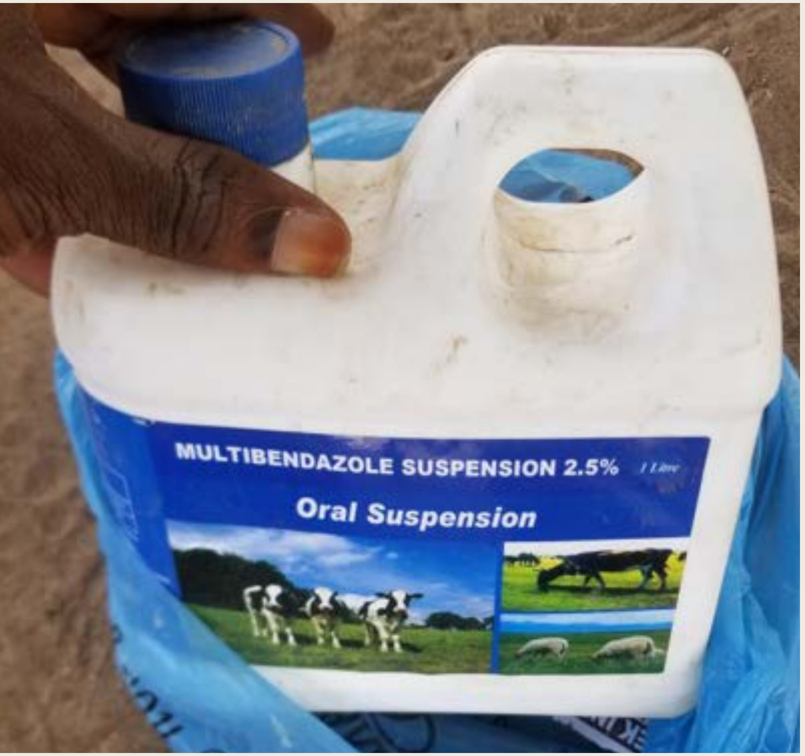

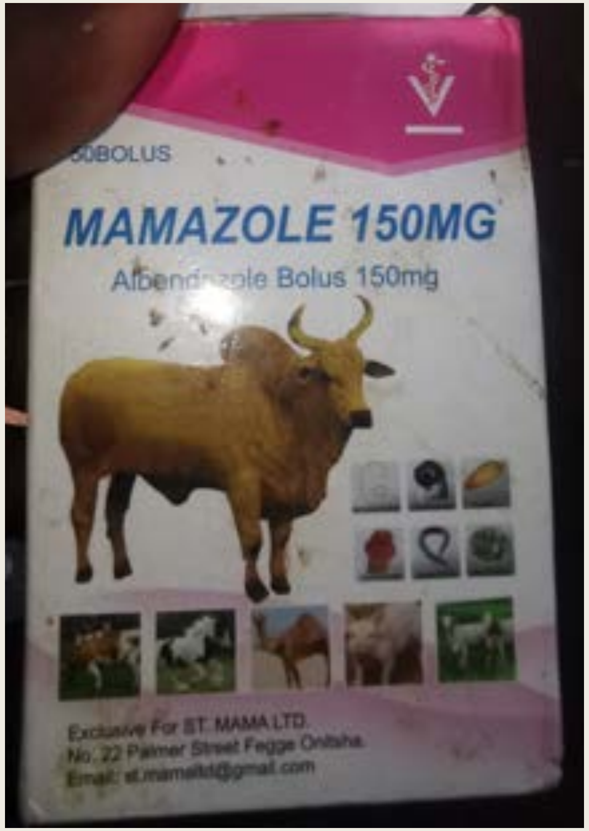

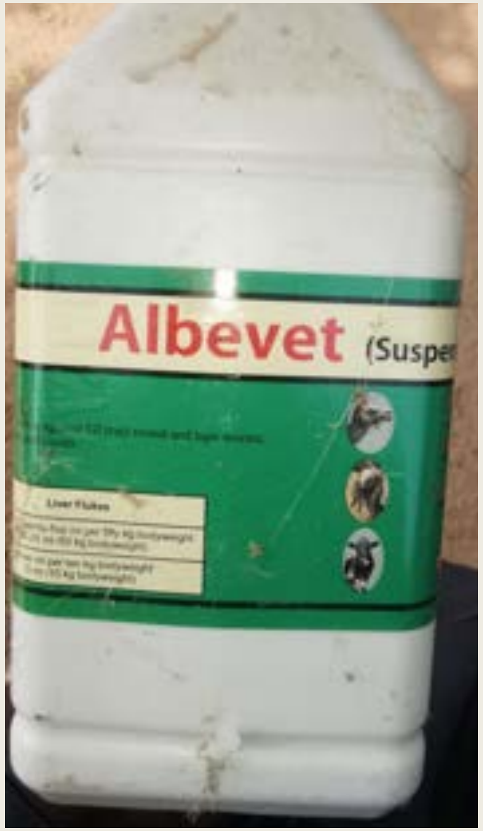

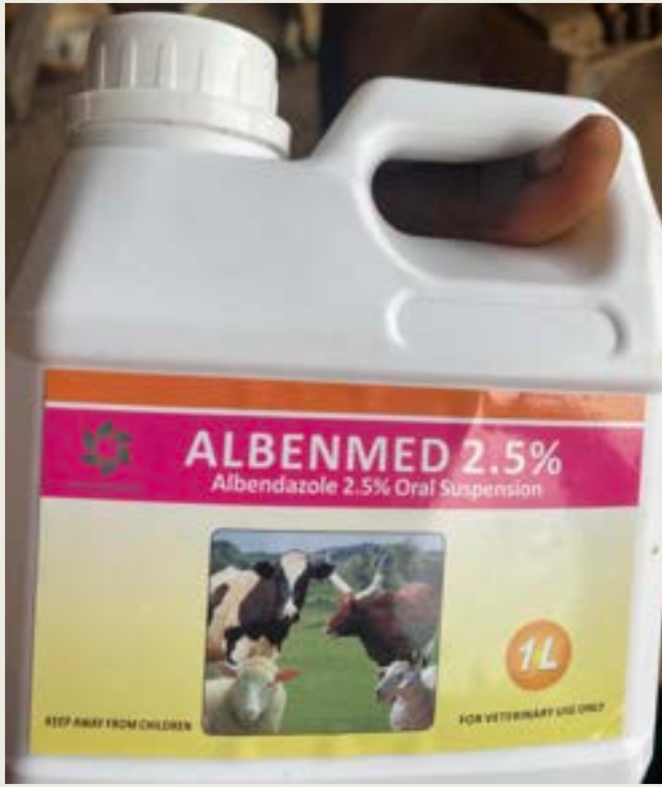

ANTIPARASITIC MEDICINES 3/6

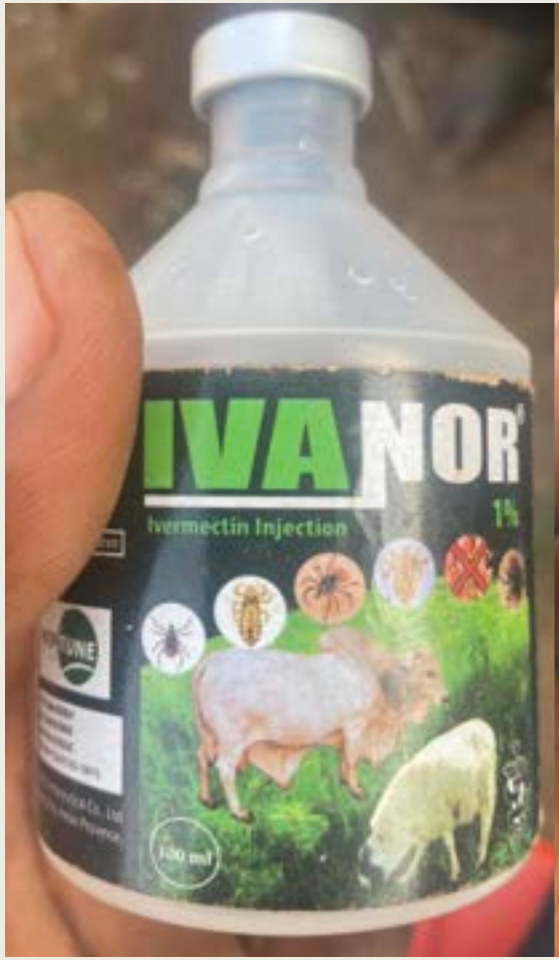

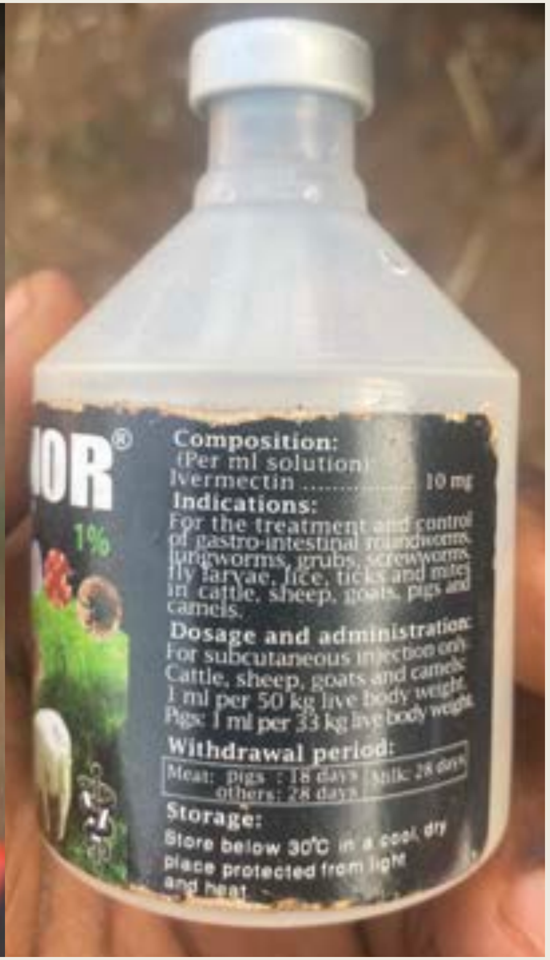

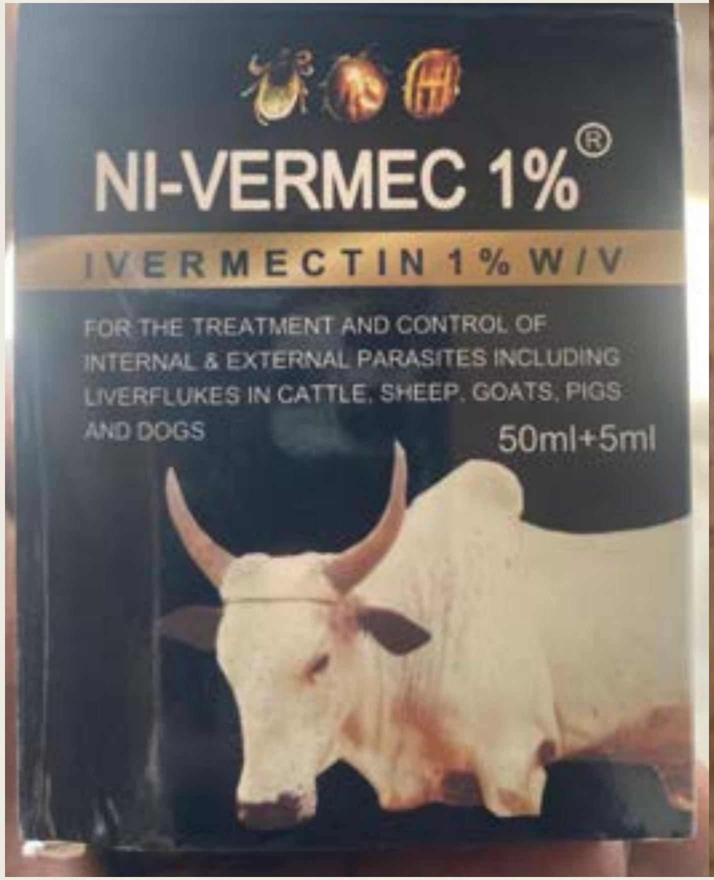

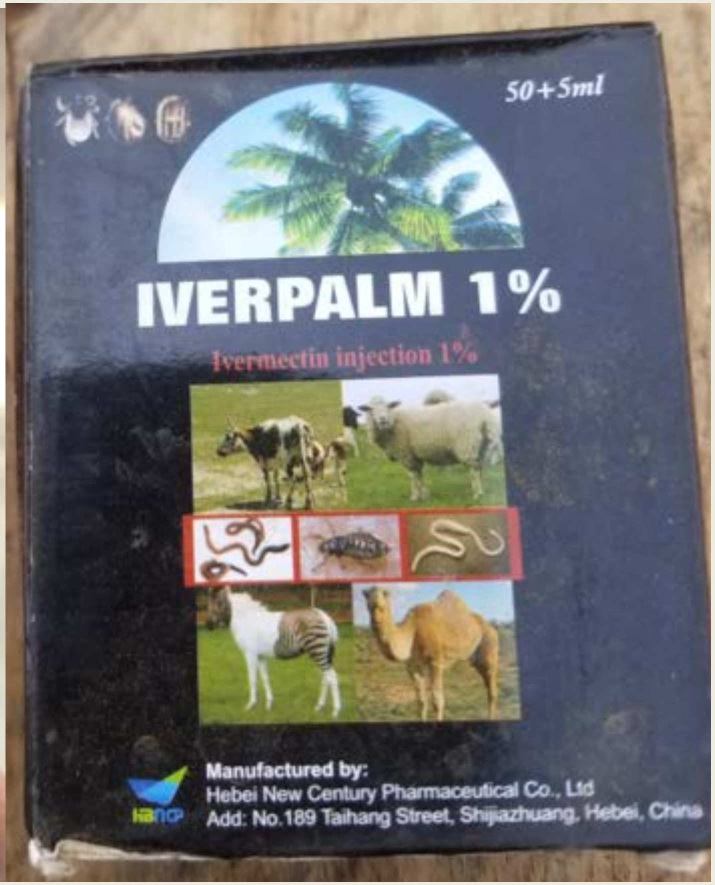

ANTIPARASITIC MEDICINES 4/6

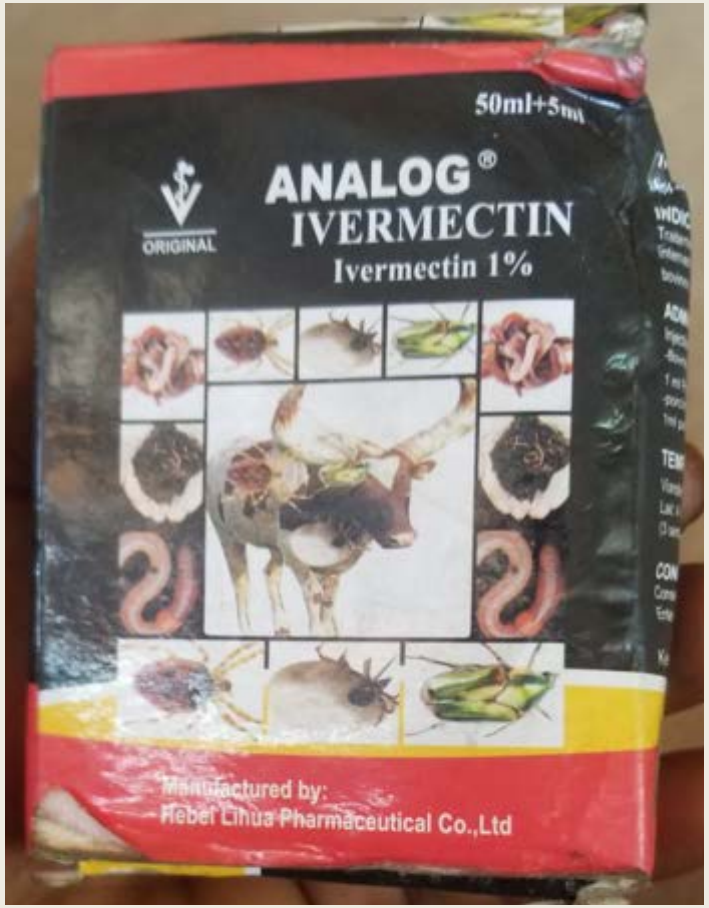

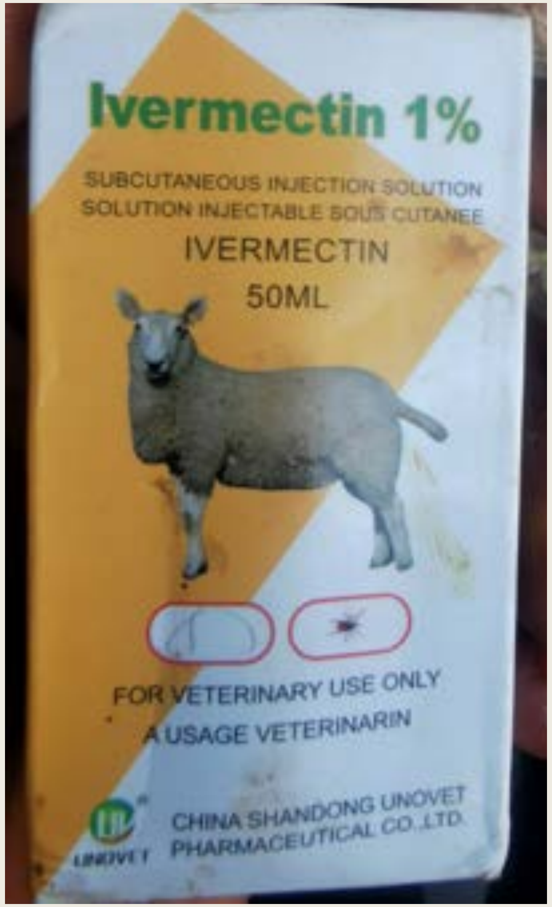

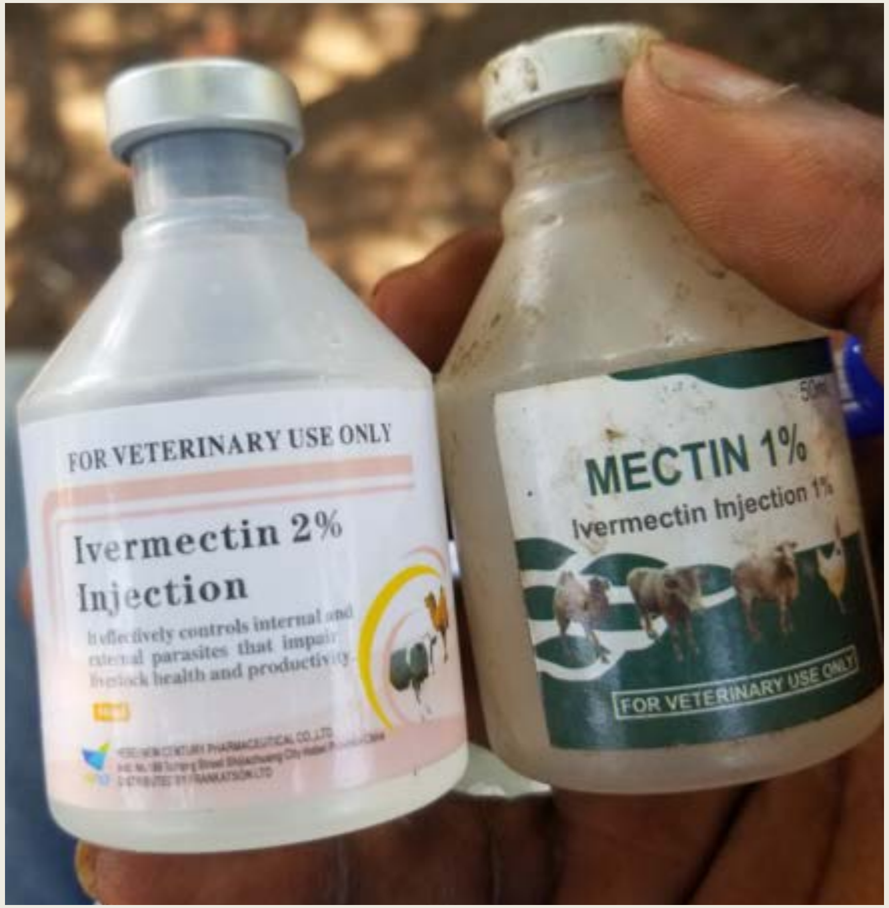

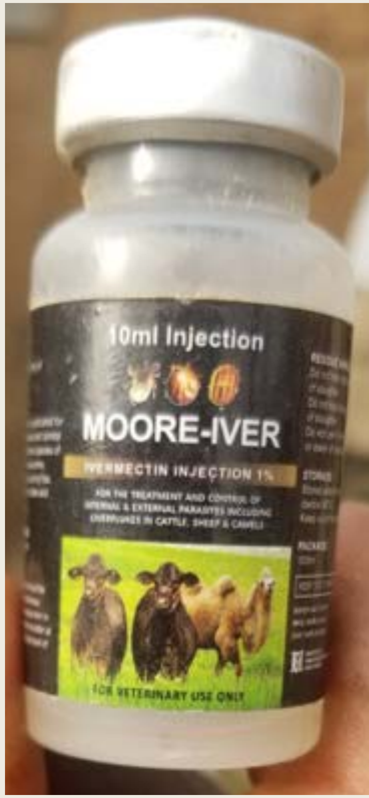

ANTIPARASITIC MEDICINES 5/6

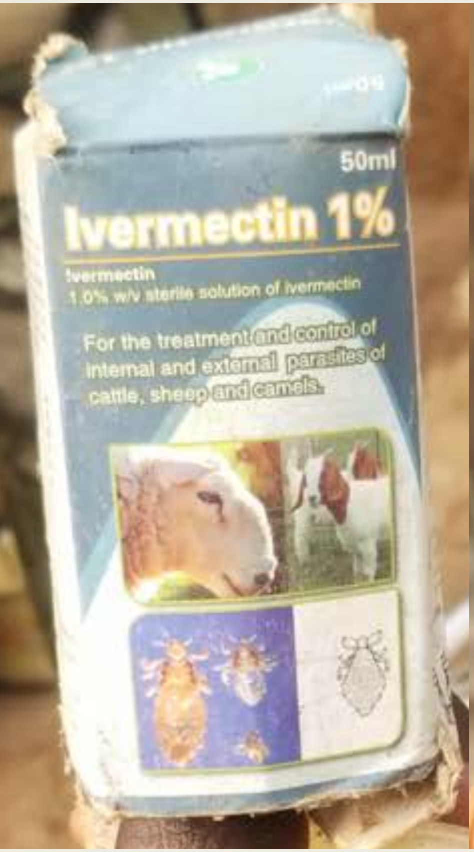

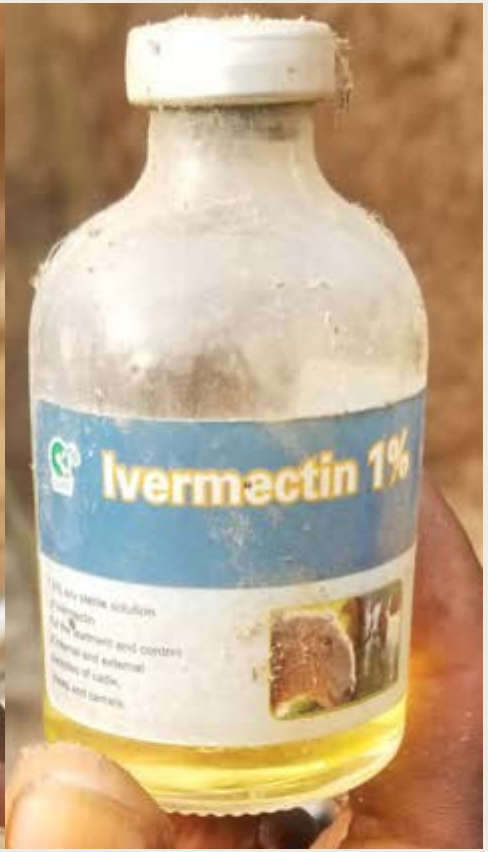

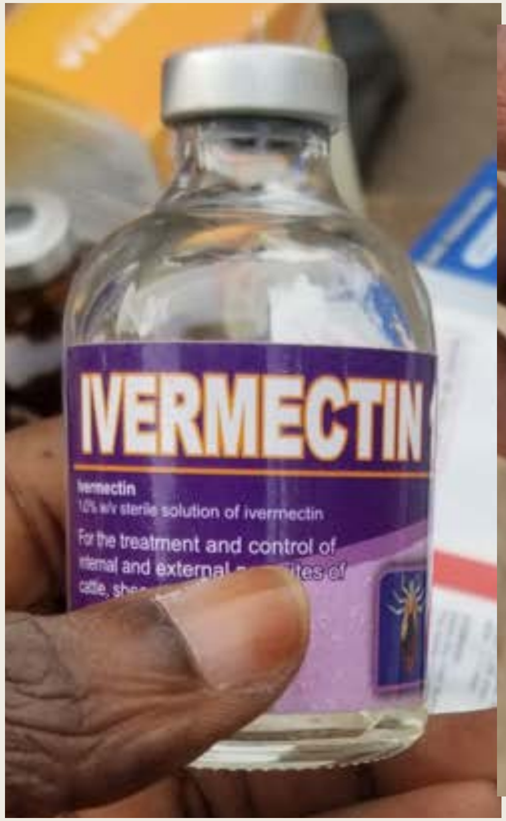

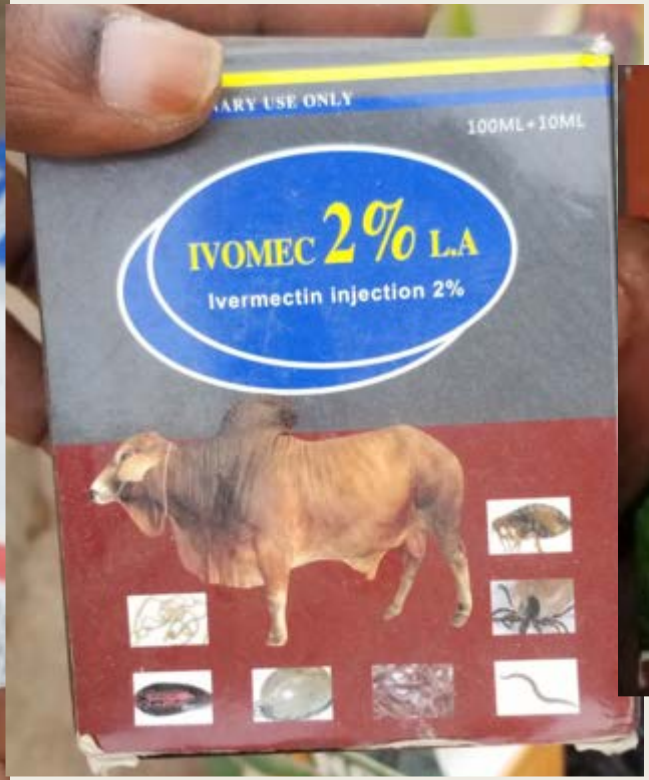

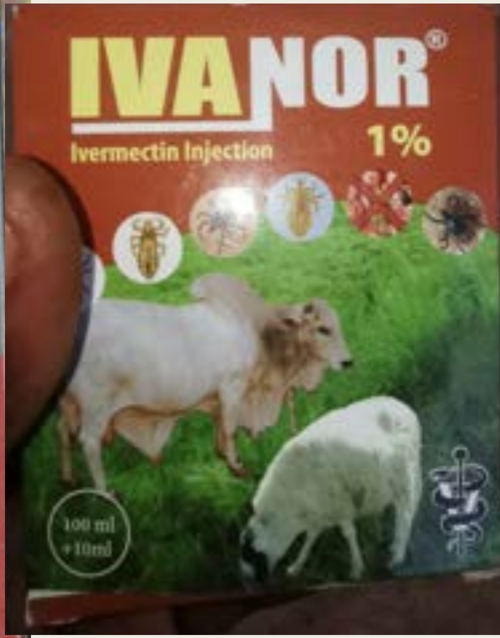

ANTIPARASITIC MEDICINES 6/6

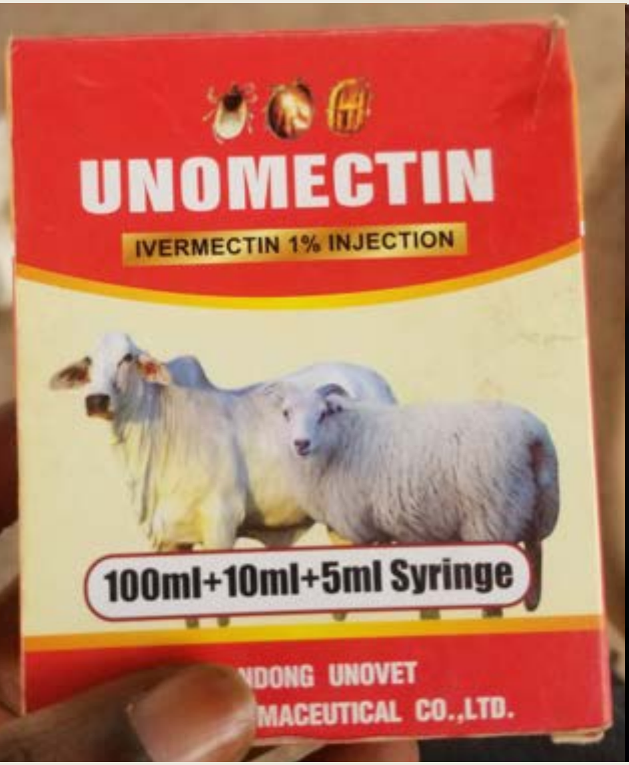

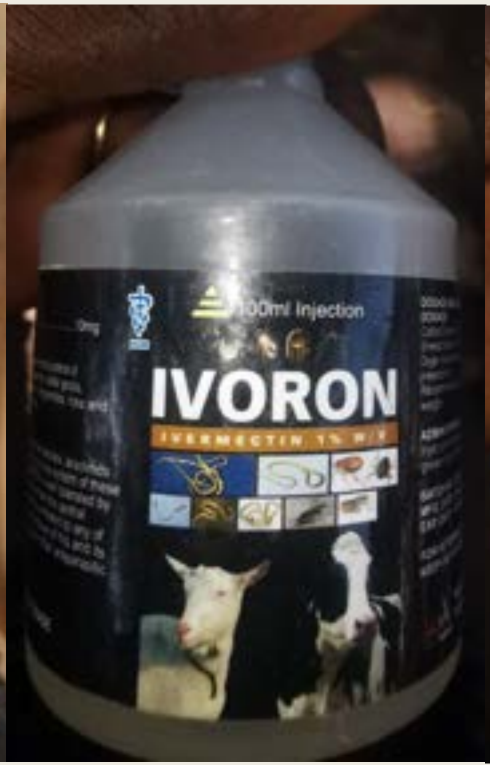

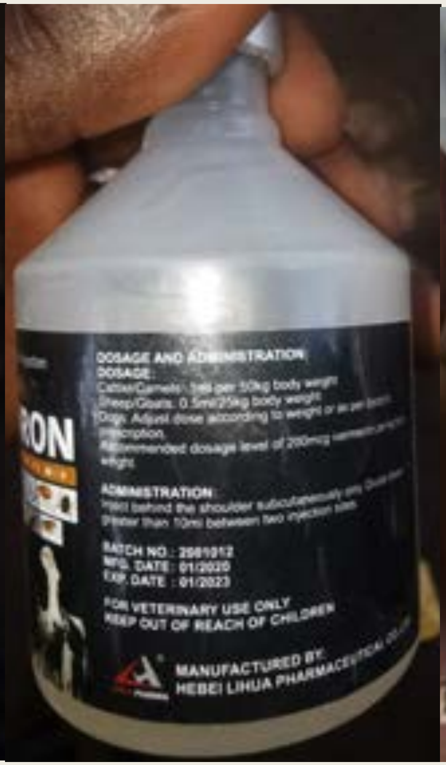

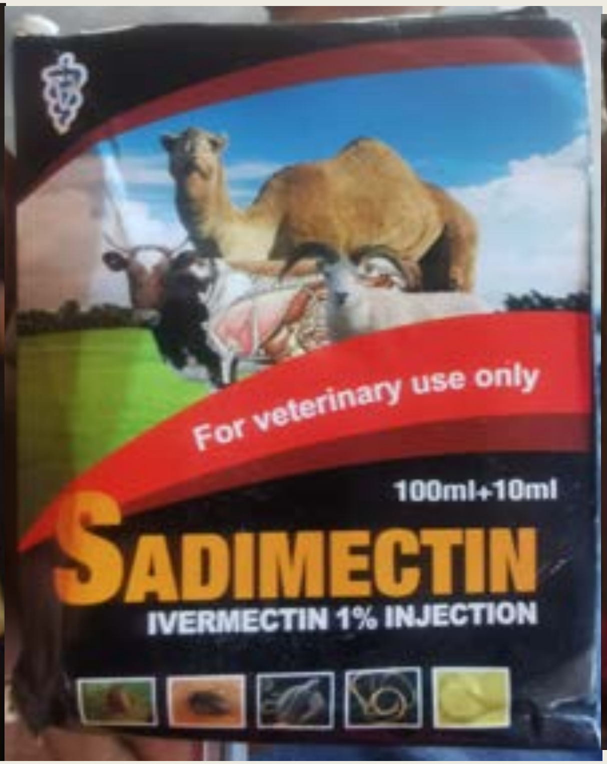

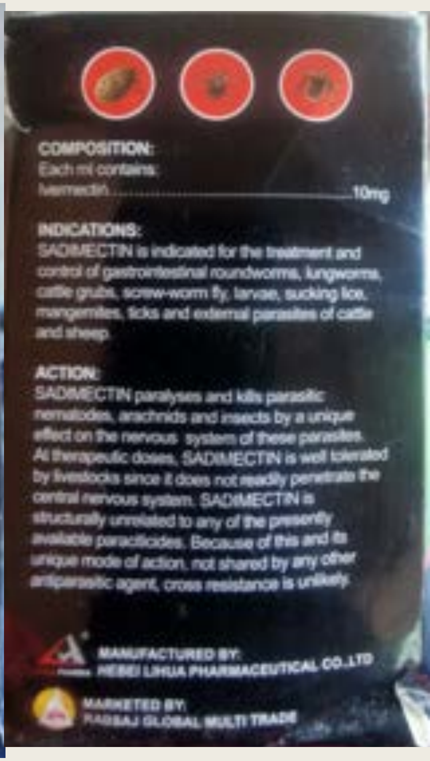

MULTIVITAMINS 1/2

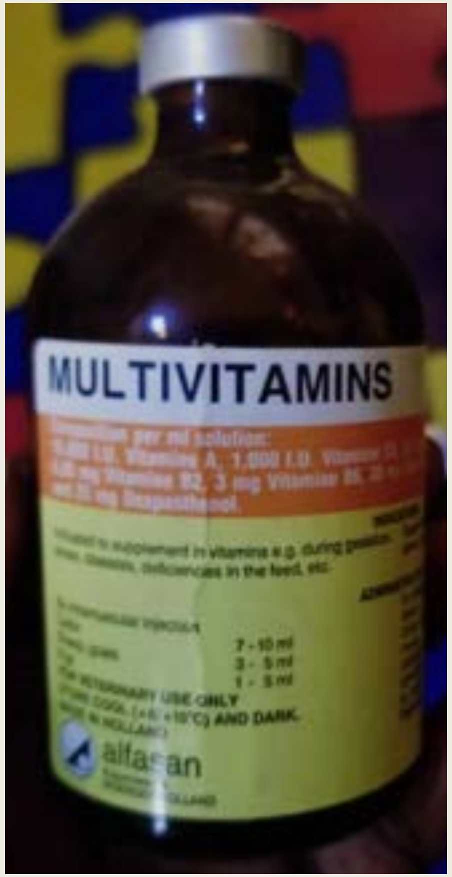

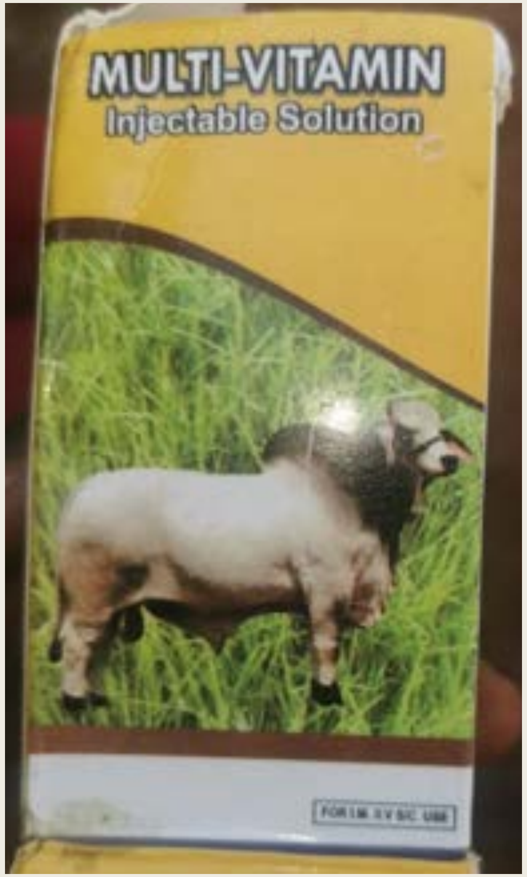

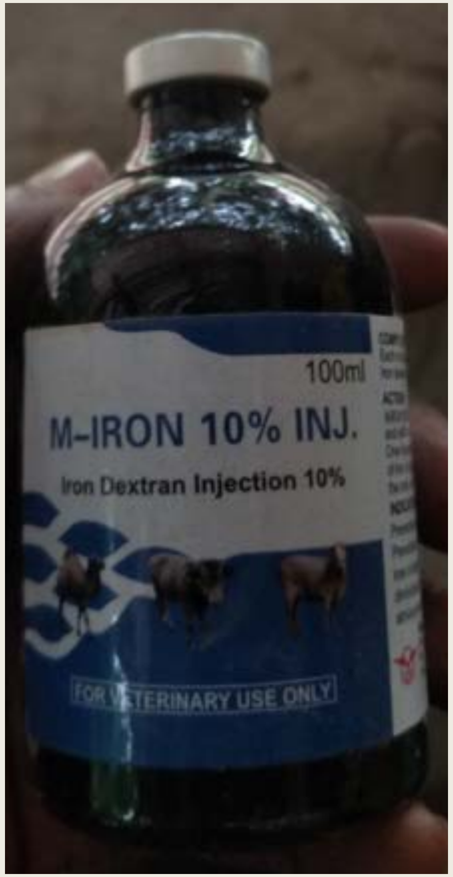

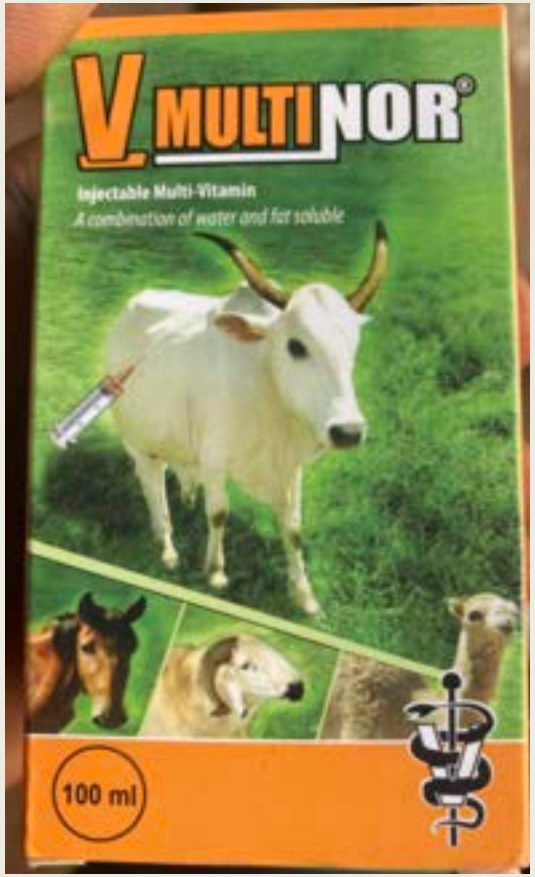

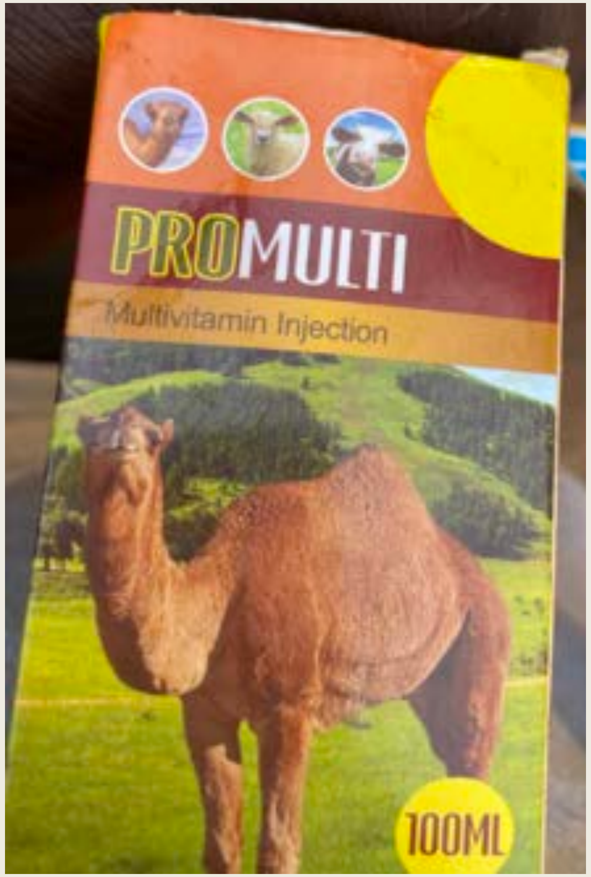

MULTIVITAMINS 2/2

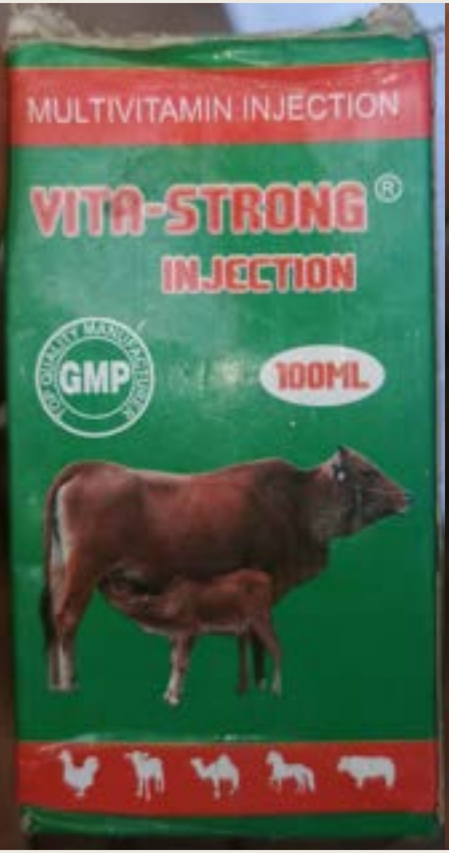

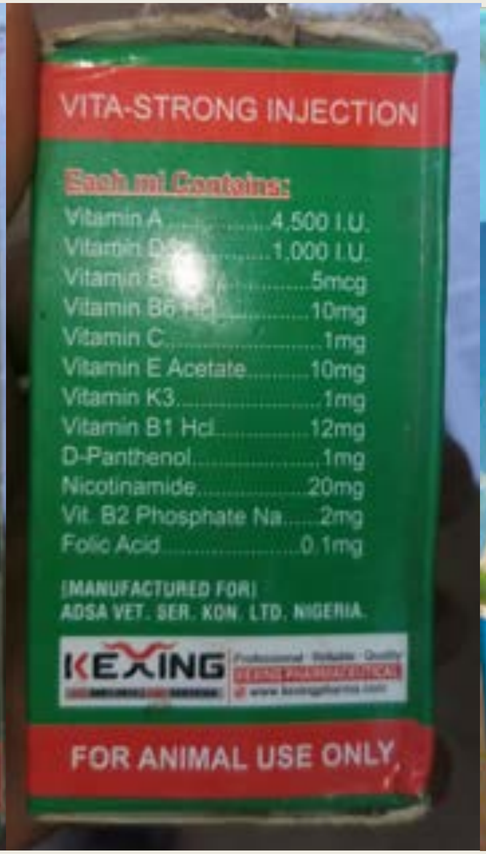

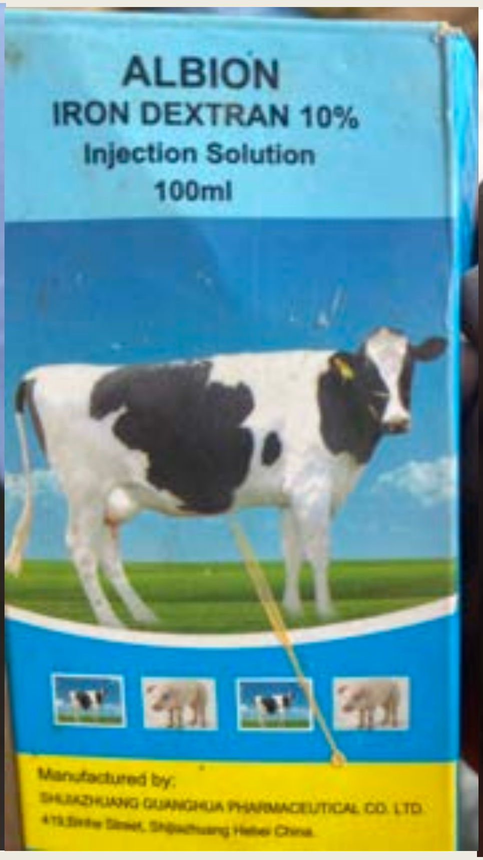

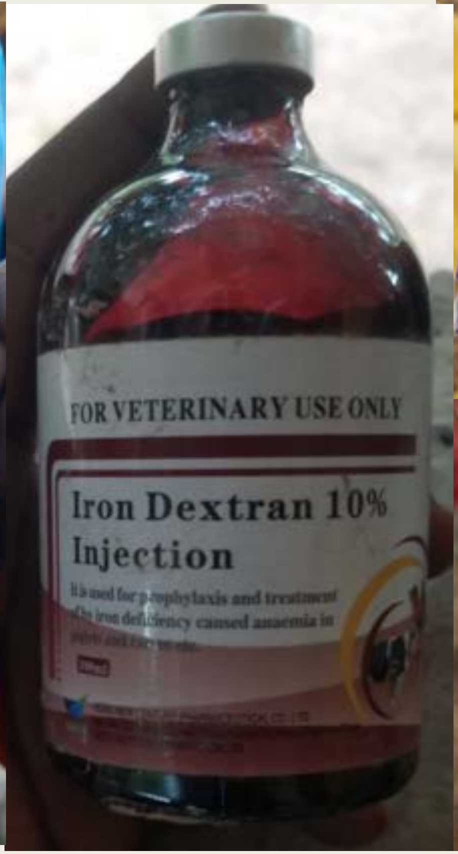

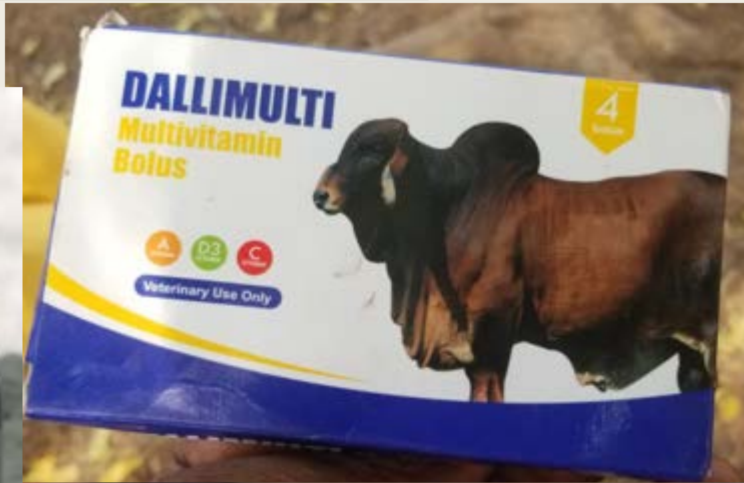

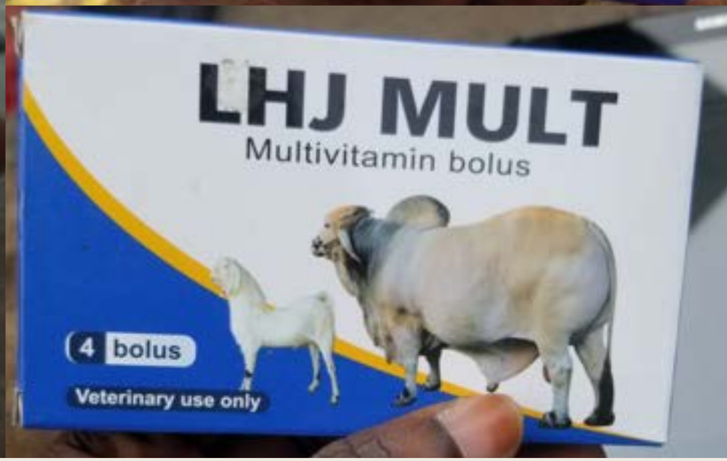

HERBAL/ TRADITIONAL MEDICINES

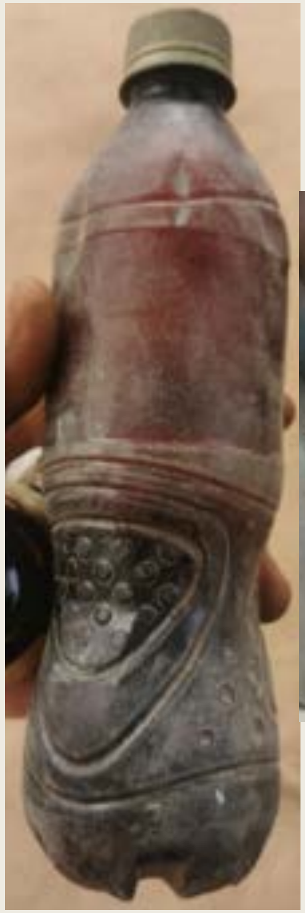

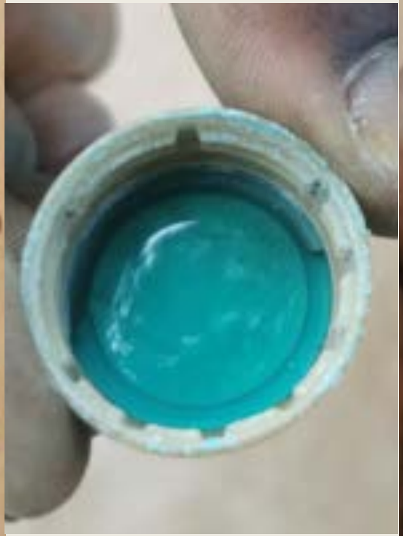

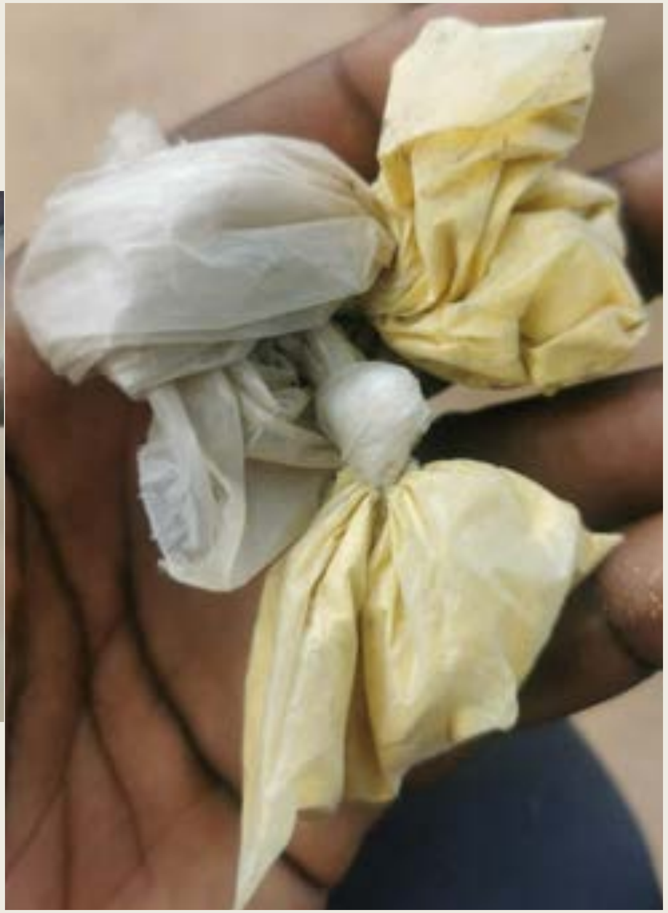

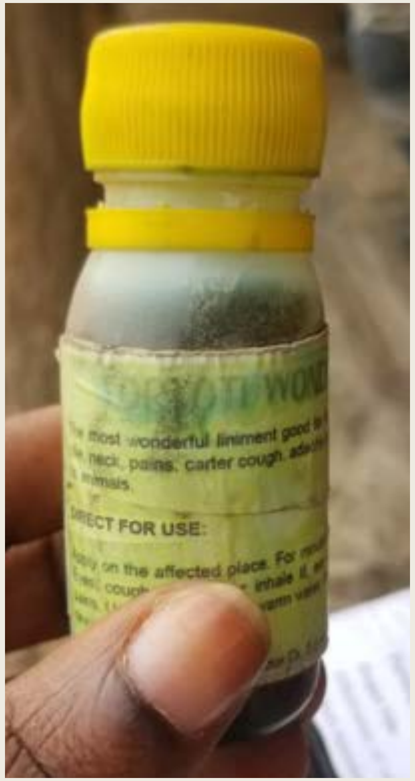

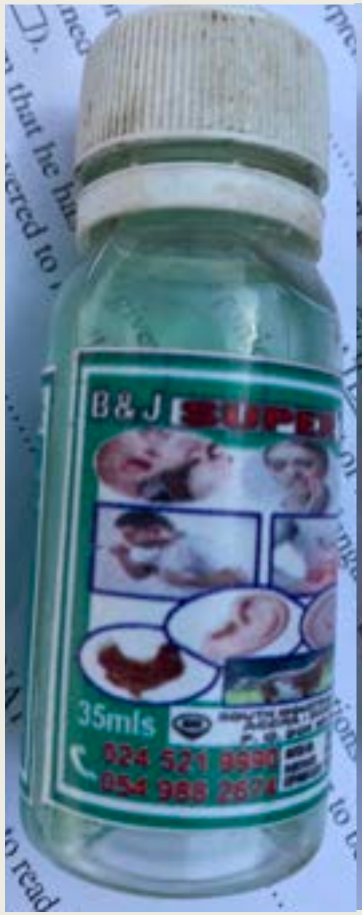

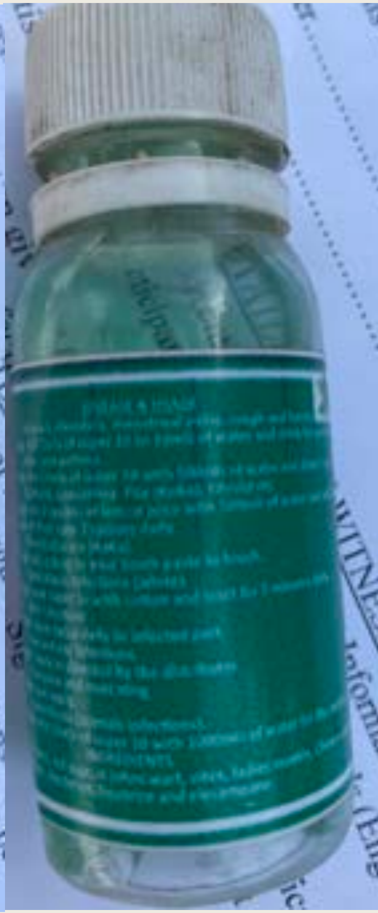

OTHER MEDICINES

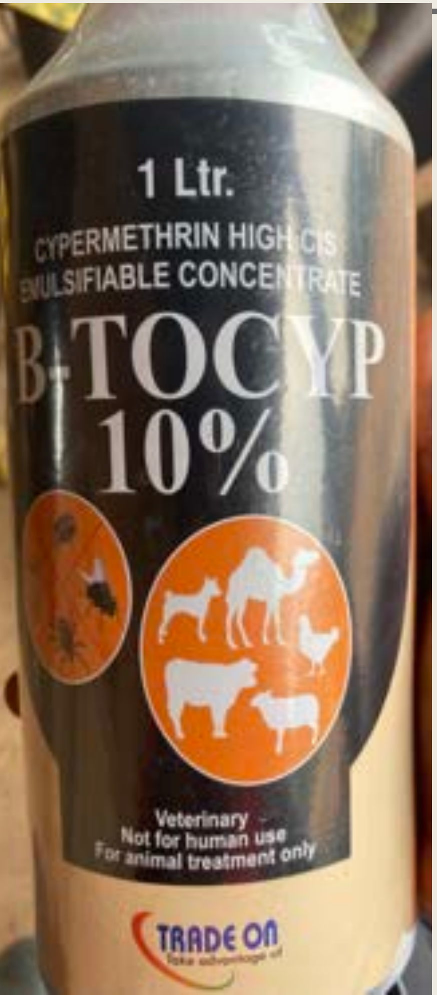

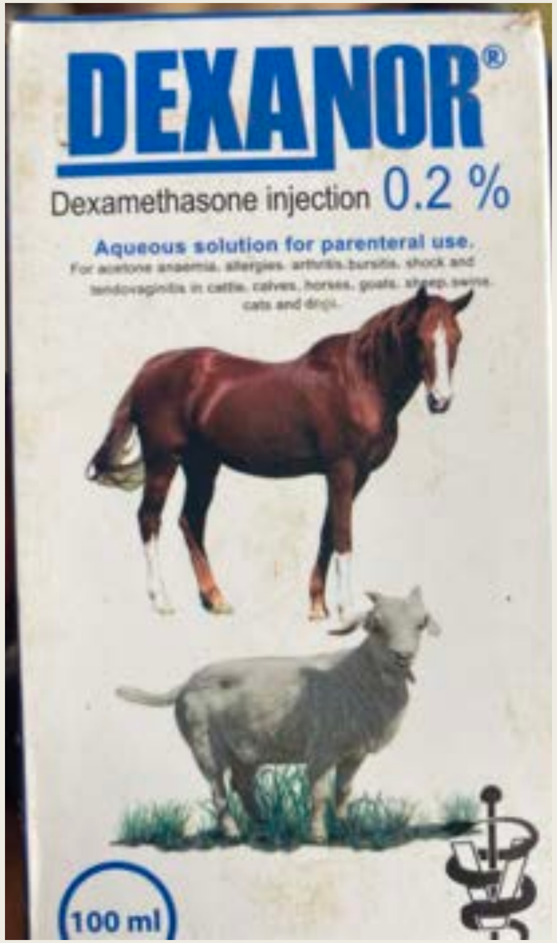

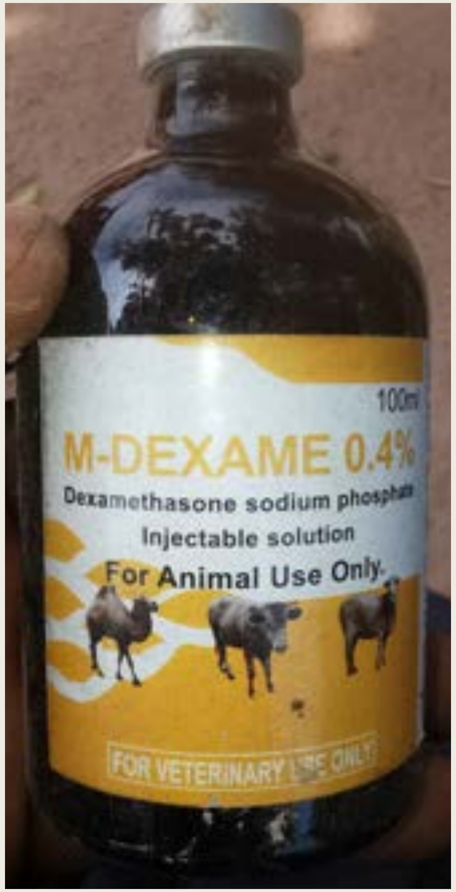

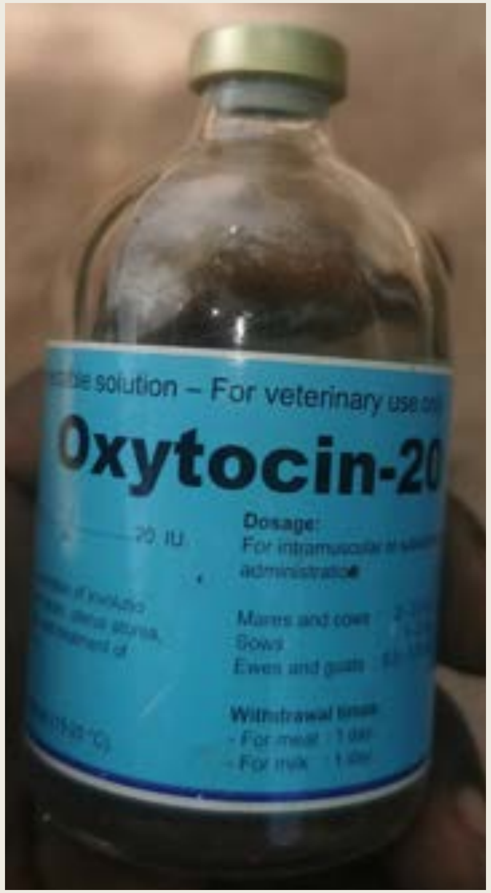

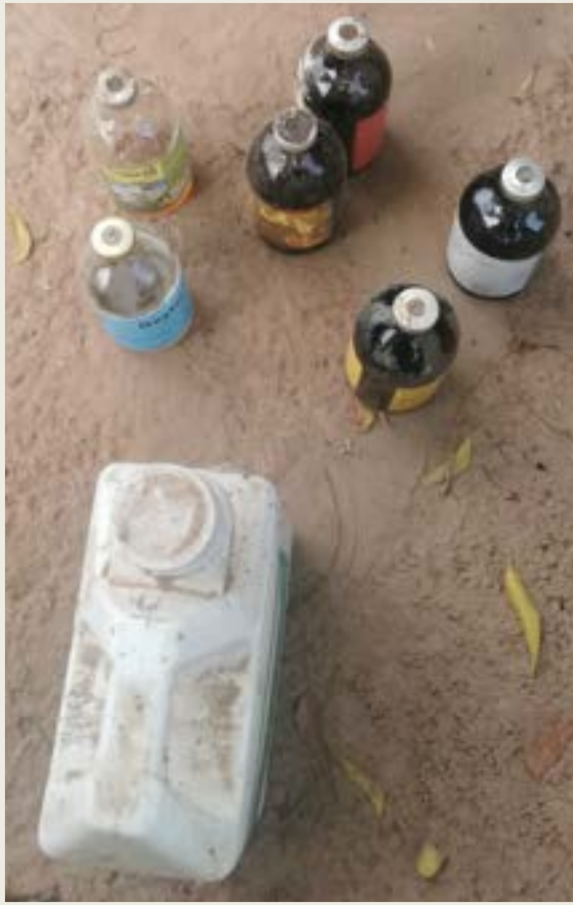

Supplement: Supplementary file 3 — Additional file 3. Samples of different types of medicines used by livestock farmers in Ghana. [file 12917_2023_3793_MOESM3_ESM.pdf]
